# Multi-omics integration identifies key upstream regulators of pathomechanisms in hypertrophic cardiomyopathy due to truncating *MYBPC3* mutations

**DOI:** 10.1186/s13148-021-01043-3

**Published:** 2021-03-23

**Authors:** J. Pei, M. Schuldt, E. Nagyova, Z. Gu, S. el Bouhaddani, L. Yiangou, M. Jansen, J. J. A. Calis, L. M. Dorsch, C. Snijders Blok, N. A. M. van den Dungen, N. Lansu, B. J. Boukens, I. R. Efimov, M. Michels, M. C. Verhaar, R. de Weger, A. Vink, F. G. van Steenbeek, A. F. Baas, R. P. Davis, H. W. Uh, D. W. D. Kuster, C. Cheng, M. Mokry, J. van der Velden, F. W. Asselbergs, M. Harakalova

**Affiliations:** 1 Division Heart and Lungs, Department of Cardiology, University Medical Center Utrecht, University of Utrecht, 3584 CT Utrecht, The Netherlands; 2 Regenerative Medicine Utrecht (RMU), University Medical Center Utrecht, University of Utrecht, 3584 CT Utrecht, The Netherlands; 3 Department of Nephrology and Hypertension, DIG-D, UMC Utrecht, University of Utrecht, Utrecht, The Netherlands; 4 Department of Physiology, Amsterdam UMC, Vrije Universiteit Amsterdam, Amsterdam, The Netherlands; 5 Laboratory of Clinical Chemistry and Hematology, UMC Utrecht, Utrecht, The Netherlands; 6 Department of Biostatistics and Research Support, UMC Utrecht, University of Utrecht, Utrecht, The Netherlands; 7 Department of Anatomy and Embryology, LUMC, Leiden, The Netherlands; 8 Department of Genetics, Division of Laboratories, Pharmacy and Biomedical Genetics, UMC Utrecht, University of Utrecht, Utrecht, The Netherlands; 9 Department of Medical Biology, AMC, Amsterdam, The Netherlands; 10 Department of Biomedical Engineering, GWU, Washington, DC USA; 11 Department of Cardiology, Thoraxcentre, Erasmus Medical Centre, Rotterdam, The Netherlands; 12 Department of Pathology, UMC Utrecht, University of Utrecht, Utrecht, The Netherlands; 13 Department of Clinical Sciences, Faculty of Veterinary Medicine, University of Utrecht, Utrecht, The Netherlands; 14 Division of Paediatrics, UMC Utrecht, University of Utrecht, Utrecht, The Netherlands; 15 Health Data Research UK and Institute of Health Informatics, University College London, London, UK; 16 Institute of Cardiovascular Science, Faculty of Population Health Sciences, University College London, London, UK; 17 Division Heart and Lungs, Department of Cardiology, University Medical Center Utrecht, Room E03.818, P.O. Box 85500, 3508 GA Utrecht, The Netherlands

**Keywords:** HCM, MYBPC3, Histone acetylome, Transcriptome, Proteome, Transcription factors

## Abstract

**Background:**

Hypertrophic cardiomyopathy (HCM) is the most common genetic disease of the cardiac muscle, frequently caused by mutations in *MYBPC3*. However, little is known about the upstream pathways and key regulators causing the disease. Therefore, we employed a multi-omics approach to study the pathomechanisms underlying HCM comparing patient hearts harboring *MYBPC3* mutations to control hearts.

**Results:**

Using H3K27ac ChIP-seq and RNA-seq we obtained 9310 differentially acetylated regions and 2033 differentially expressed genes, respectively, between 13 HCM and 10 control hearts. We obtained 441 differentially expressed proteins between 11 HCM and 8 control hearts using proteomics. By integrating multi-omics datasets, we identified a set of DNA regions and genes that differentiate HCM from control hearts and 53 protein-coding genes as the major contributors. This comprehensive analysis consistently points toward altered extracellular matrix formation, muscle contraction, and metabolism. Therefore, we studied enriched transcription factor (TF) binding motifs and identified 9 motif-encoded TFs, including *KLF15, ETV4*, *AR, CLOCK, ETS2, GATA5, MEIS1, RXRA,* and *ZFX*. Selected candidates were examined in stem cell-derived cardiomyocytes with and without mutated *MYBPC3*. Furthermore, we observed an abundance of acetylation signals and transcripts derived from cardiomyocytes compared to non-myocyte populations.

**Conclusions:**

By integrating histone acetylome, transcriptome, and proteome profiles, we identified major effector genes and protein networks that drive the pathological changes in HCM with mutated *MYBPC3*. Our work identifies 38 highly affected protein-coding genes as potential plasma HCM biomarkers and 9 TFs as potential upstream regulators of these pathomechanisms that may serve as possible therapeutic targets.

**Supplementary information:**

The online version contains supplementary material available at 10.1186/s13148-021-01043-3.

## Introduction

Hypertrophic cardiomyopathy (HCM), characterized by thickening of the myocardium that is not explained by abnormal loading conditions, is the most common inherited cardiac disease [1]. More than 1,500 associated mutations, primarily in genes encoding parts of the sarcomere, such as *MYBPC3*, *MYH7,* and *TNNT2*, have been identified. The remodeled myocardium is characterized by cardiomyocyte hypertrophy and disarray, extensive fibrosis, and reduced capillary density [2]. HCM is a heterogeneous disease as the onset, the disease phenotype, and the severity of the clinical presentations differ greatly among mutation carriers [3]. Additionally, different mutated genes exhibit distinct biological impact on cellular contractility, energy metabolism, and sarcomeric protein expression in HCM hearts [4]. However, the driving pathological mechanisms underlying the heterogenous HCM remain largely unknown.

Next-generation sequencing technologies (NGS) are instrumental in understanding disease etiology, delivering a clinical diagnosis, and discovering new treatment options. Multiple studies employed NGS to reveal the epigenetic modifications in heart failure, including DNA methylation and histone (de-)acetylation, which provided insights and identification of the driving mechanism underlying the disease [5, 6]. We previously showed the influence of histone acetylation changes on QRS complex-related GWAS loci in HCM [7, 8]. Additionally, studies from our group and others have shown that H3K27ac corresponds to the gene expression in human cardiac tissues as well as human cardiomyocytes [9, 10], highlighting it as a promising epigenetic mark for predicting gene expression. Studies also employed RNA sequencing to identify the affected transcription factor-mediated upstream regulatory events and the distinct gene expressions that define heart failure [9, 10]. Furthermore, data-piling studies are now connecting proteomics to NGS to get comprehensive information on the disease biology for precision medicine [11].

In this study, we aim to understand the pathomechanisms driving HCM by employing a multi-omics approach, including chromatin immunoprecipitation sequencing (ChIP-seq), RNA sequencing (RNA-seq), and proteomics, using myocardial tissue obtained from clinically well-phenotyped HCM patients with truncating *MYBPC3* mutations and compared these with non-failing donor hearts. We revealed altered histone acetylome, transcriptome, and proteome profiles in HCM versus control hearts and studied affected biological functions. Besides, we identified key factors that may play a critical role in regulating the pathomechanisms underlying HCM. We also evaluated the contribution of histone acetylation and transcription signals in 11 cell types in the heart. Combined, this multi-omics study gives insight into the underlying disease pathways driving HCM and identifies promising candidates for therapeutic strategies.

## Results

### Pairwise comparison between HCM and control hearts reveals distinct histone acetylome profiles

**Fig. 1:**
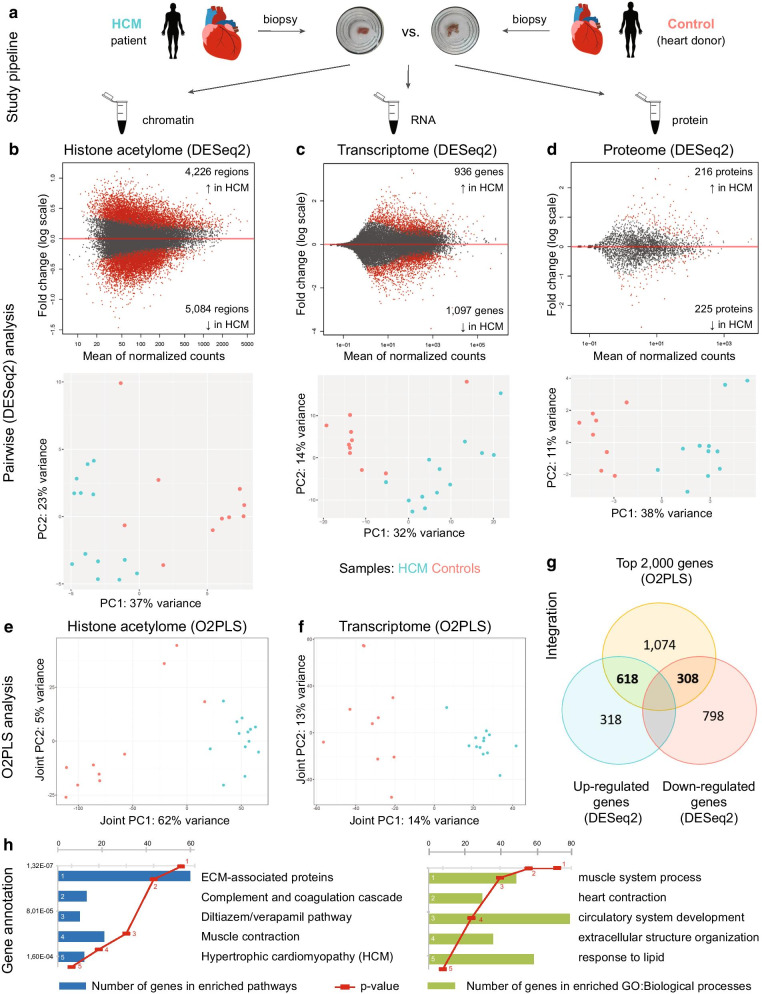
Pairwise analyses using DESeq2 and integrative analyses using unsupervised O2PLS in all samples. **a** An overview of the study design. **b** Upper plot: MA (ratio intensity) plot showing the hyper- and hypoacetylation regions in HCM samples compared to controls using DESeq2; bottom plot: principal component analysis (PCA) plot showing the separation between HCM and control samples based on the top differentially acetylated regions using DESeq2. Mean values of normalized counts in all samples are depicted on the x-axis and fold changes (log2) on the y-axis. **c** Upper plot: PCA plot showing the separation between HCM and control samples based on the top differentially expressed genes using DESeq2; bottom plot: MA plot showing the up-regulated and down-regulated genes in HCM samples compared to controls using DESeq2. **d** Upper plot: PCA plot showing the separation between HCM and control samples based on the top differentially expressed proteins using DESeq2; bottom plot: MA plot showing the up-regulated and down-regulated proteins in HCM samples compared to controls using DESeq2. **e** Score plot of the first joint component of the H3K27ac ChIP-seq data showing the separation of HCM hearts from controls. **f** Score plot of the first joint component of the RNA-seq data discriminating HCM hearts from controls. **g** Venn diagram showing the overlapping targets between the top 2,000 genes obtained using the integrative approach (O2PLS) and differentially expressed genes obtained using the pairwise comparison (DESeq2). **h** Enrichment analyses using the overlapping targets, which included 618 up-regulated genes and 308 down-regulated genes

H3K27ac ChIP-seq was used to capture acetylated DNA regions in each sample and to compare the acetylation levels between 13 HCM (*n* = 13) and 10 control hearts (Fig. 1a and Additional file 9: Table S1) using DESeq2. In total, we identified 4,226 regions presented higher acetylation levels (hyperacetylated regions) and 5,084 regions presented lower acetylation levels (hypoacetylated regions) in HCM versus control hearts (*P*_adj_ < 0.05, Fig. 1b, Additional file 10: Table S2A). Examples of hyper- and hypoacetylated regions are shown in Additional file 1: Figure S1. Region-to-gene annotation using either a 5 kb or 50 kb window from the transcription start site (TSS) revealed genes in the hyperacetylated regions are mostly involved in muscle contraction and extracellular matrix (ECM) development-related processes, whereas genes in the hypoacetylated regions are mostly involved in metabolic processes (Additional file 10: Table S2B-S2E).

### Pairwise comparison between HCM and control hearts reveals distinct transcriptome profiles

Following RNA-seq of the same biopsies, the transcriptional profiles between 13 HCM (*n* = 13) and 10 control hearts were compared using DESeq2. In total, we identified 936 up-regulated genes and 1,097 down-regulated genes in HCM hearts compared to controls (*P*_adj_ < 0.05, Fig. 1c, Additional file 11: Table S3A). The top biological processes enriched by up-regulated genes are involved in the muscle system process and energy production. The top biological processes enriched by down-regulated genes are involved in lipid metabolism and cell adhesion (Additional file 11: Table S3B and S3C).

### Pairwise comparison between HCM and control hearts reveals distinct proteome profiles

We also performed proteomics in 11 HCM samples and compared their protein expression levels to another control group (*n* = 8) using DESeq2. In total, we identified 216 up-regulated proteins and 225 down-regulated proteins in HCM hearts compared to controls (*P* < 0.05, Fig. 1d, Additional file 12: Table S4A). The top enriched biological processes by up-regulated proteins are involved in muscular and ECM development. The top enriched biological processes by down-regulated proteins are involved in metabolism (Additional file 2: Figure S2, Additional file 12: Table S4B, and S4C).

### Integrating histone acetylome, transcriptome, and proteome changes in HCM

We also integrated H3K27ac ChIP-seq and RNA-seq data in an unsupervised manner using O2PLS. Notably, without predefining the patient and the control groups, the first joint component of both ChIP-seq and RNA-seq plots discriminated HCM from control hearts (Fig. 1e, f). DNA regions and genes that contributed to the separation between HCM and control hearts are shown in Additional file 13: Table S5. Next, we aimed to identify key genes that underlie the separation between two groups and show altered expression levels in HCM hearts versus controls. Therefore, we overlapped 2,000 genes that discriminate HCM hearts and controls from the integrative analysis (O2PLS) with the differentially expressed genes from the pairwise comparison with the top and obtained 618 up-regulated genes and 308 down-regulated genes (Fig. 1g and Additional file 14: Table S6A). These overlapping genes are enriched for biological processes in the circulatory system and muscle contraction and pathways involved in the ECM formation and complement system (Fig. 1h, Additional file 14: Table S6B).

Since only 11 out of 13 HCM samples were included in the proteomics experiment and a different set of control samples was used in comparison with the HCM group, we could not apply O2PLS to integrate the proteomic data with either H3K27ac ChIP-seq or RNA-seq data. We, therefore, overlapped differentially expressed genes supported by DESeq2 and O2PLS analyses with proteomic data and identified 36 up-regulated protein-coding genes and 17 down-regulated protein-coding genes in HCM versus control hearts (Table 1). Notably, the protein levels of 38 protein-coding genes are detectable in the plasma and 5 out of them were consistently changed in the same direction in HCM versus control hearts at DNA (5 kb from TSS), RNA, and protein levels, including the up-regulation of ASPN, FMOD, MCAM, and NPPA and the down-regulation of AASS, highlighting them as promising candidates for biomarker discovery in HCM (Table 1).

**Table 1:** Promising candidates showing the same changing direction in HCM versus control hearts at multi-omics levels

Symbol	Known expression level in cardiomyocytes*	Protein level in the plasma/serum^‡^	Rank in the O2PLS analysis	Transcriptome change (log2FC)	Proteome change (log2FC)	Histone acetylome change in the matched direction (50 Kb of TSS)	Transcriptome change in diseased versus control cardiomyocytes^ϯ^ log2(FC)
AASS	Medium	Detected	1046	− 1.279808444	− 0.773247332	Yes	− 0.414920406
ABHD11	Medium	Detected	669	1.137180651	0.640971138	–	–
ACTN2	High	Detected	1597	0.570095367	0.184641682	–	–
ADH1B	Low	Detected	1281	− 1.081956134	− 0.880289941	–	–
ARHGAP1	Medium	Detected	1512	1.763584	0.597291165	Yes	–
ASPN	Not detected	Detected	454	2.204317	1.77725136	Yes	–
ATP2A2	High	Detected	728	− 1.466783433	− 0.353968239	–	–
BGN	Medium	Detected	1012	1.832538	1.533815477	–	0.588280947
C6	Not detected	Detected	130	− 1.776237721	− 0.695516121	–	–
CA3	Not detected	Detected	43	4.518344	1.686864533	–	–
CHCHD6	Medium	Not detected	910	2.003514	0.778880421	–	–
CHDH	Low	Not detected	98	− 2.209806815	− 1.442995937	Yes	–
CLGN	Not detected	Not detected	1751	− 1.088152172	− 0.808838009	Yes	–
DDAH1	Not detected	Detected	452	1.764206	1.115473264	Yes	–
EFHD1	Not detected	Not detected	682	1.865517	1.264459654	Yes	0.318203232
FGF12	No data	Not detected	62	− 2.353022973	− 1.079274106	Yes	− 0.30374396
FHL2	High	Not detected	440	− 1.255001122	− 0.411782639	–	–
FMOD	No data	Detected	395	2.594323	1.414783229	Yes	–
FSCN1	Not detected	Detected	1276	1.759951	0.971207984	Yes	0.456046007
GATM	Not detected	Detected	277	1.545734889	1.002695568	Yes	–
GPD1	Medium	Detected	1708	− 1.687472289	− 1.343895776	Yes	− 0.410181446
GPD1L	No data	Not detected	1342	− 1.066784553	− 0.526757351	–	–
HSPA2	Not detected	Detected	171	3.490234	2.652261545	Yes	–
HSPB6	High	Detected	647	2.253803	0.392977171	–	− 3.170003763
LDHA	Not detected	Detected	974	− 1.219034333	− 0.474108409	Yes	–
LTBP2	Not detected	Detected	829	2.1279	2.317830122	Yes	–
LUM	Not detected	Detected	217	2.135187	0.959297891	–	0.512857369
MAP4	Medium	Detected	717	1.851811	1.003525943	Yes	− 0.223635511
MCAM	Low	Detected	1338	1.653115	1.104716402	Yes	− 0.264831197
MFAP2	Not detected	Not detected	1303	1.84358	0.70531399	Yes	− 0.357062335
MYH6	High	Detected	40	− 2.919038787	− 1.009056575	–	–
MYL12A	Medium	Detected	64	2.419604	0.578322489	–	− 0.14329672
MYLK3	High	Not detected	1904	− 1.007764366	− 0.747812735	–	0.127033902
NES	Medium	Detected	641	1.806419	1.936943877	–	–
NPPA	Medium	Detected	13	1.549472808	0.747781872	Yes	0.542363796
NUDT4	High	Not detected	1531	− 1.093022558	− 0.695436878	–	0.207067638
PDK4	High	Not detected	1724	− 1.07459529	− 0.607585372	Yes	–
RAB24	No data	Not detected	1085	2.211788	1.041018278	–	–
RRAS	High	Detected	1642	1.788982	0.447281909	–	–
S100A6	Not detected	Detected	1486	1.859224	0.794548978	–	–
SAA1	No data	Detected	63	− 1.173021107	− 2.740052623	–	–
SERPINE2	No data	Detected	338	1.002427623	0.652055032	–	0.714236168
SGCG	High	Not detected	1805	0.707480434	0.430185184	Yes	–
SLC25A5	High	Detected	1634	1.811682	0.613247381	–	–
SNCA	Not detected	Detected	274	2.629958	1.02410125	–	–
SORBS2	No data	Detected	1991	1.475585	0.484980733	Yes	− 0.241151423
STMN1	Not detected	Detected	1211	0.77170211	0.807423435	–	–
SYNPO2L	High	Not detected	483	2.306538	1.028059491	Yes	–
TANGO2	Low	Detected	1086	1.981163	0.733156318	–	–
THBS4	Low	Detected	618	2.323482	2.106545221	–	–
TPM3	Medium	Detected	981	2.116673	1.224815654	–	0.36243013
TPPP	Not detected	Not detected	1374	− 0.898226805	− 0.830942802	Yes	–
UCHL1	Not detected	Detected	44	5.052289	0.933127805	–	–

* The known expression level of each candidate in cardiomyocyte is collected from the Human Protein Atlas (https://www.proteinatlas.org/)

^‡^The detectable protein level of each candidate in plasma and serum is collected from the Human Plasma Proteome Project Data Central (http://www.peptideatlas.org/hupo/hppp/)

^ϯ^Differentially expressed genes between programmed cardiomyocytes with and without mutated MYBPC3 from a published study were used with a threshold of *P* value below 0.05 (GSE140965)

*FC* fold change, *TSS* transcription start site, – not matching

### Combined omics analyses identify ECM, muscle contraction, and metabolism as the main mechanisms altered in HCM

**Fig. 2:**
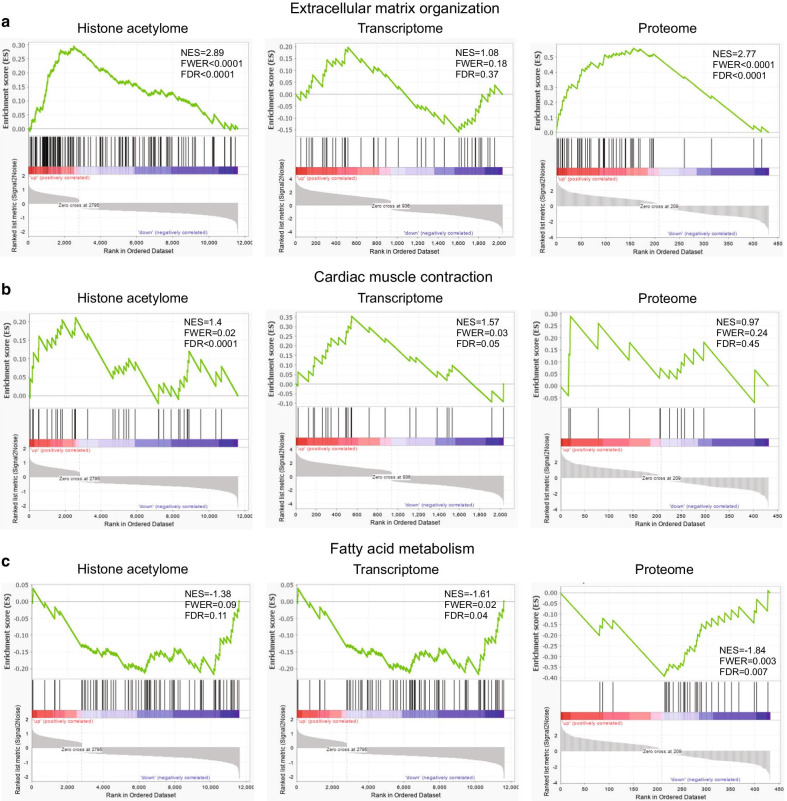
Gene set enrichment analysis showing the correlation of the gene set, which are established in extracellular matrix organization (**a**), cardiac muscle contraction (**b**), and fatty acid metabolism (**c**), in annotated genes from differentially acetylated regions (histone acetylome), differentially expressed genes (transcriptome), and genes encoding differentially expressed proteins (proteome), respectively. Differentially expressed genes were ranked by their fold changes and shown on the x-axis. The running correlation throughout the gene set is shown by the curve (green), and the running enrichment score (ES) is shown on the y-axis. Enrichment score normalized for gene set size (NES), familywise-error rate *P* value (FWER), and the false discovery rate (FDR) are shown per enrichment plot

Genes annotated to differentially acetylated regions in the histone acetylome data, differentially expressed genes in the transcriptome data, and differentially expressed proteins in the proteome data consistently pointed to biological functions involved in enhancement of ECM, enhancement of muscle contraction, and suppression of metabolism in HCM versus control hearts. To further evaluate these biological functions, we collected gene sets that are known to regulate ECM remodeling, muscle contraction, and metabolism and performed GSEA to study whether they are primarily found in the up-regulated or down-regulated data sets in our study or they are randomly distributed. We observed that genes involved in ECM and cardiac muscle contraction were positively correlated with genes annotated to the hyperacetylated regions in the ChIP-seq data, the up-regulated genes in the RNA-seq data, and genes encoding the up-regulated proteins in the proteomics data (Fig. 2a, b). Genes that are related to fatty acid metabolism were significantly correlated with genes annotated to the hypoacetylated regions in the ChIP-seq data, the down-regulated genes in the RNA-seq data, and genes encoding the down-regulated proteins in the proteomics data (Fig. 2c, Additional file 3: Figure S3). Combined, we confirmed that pathways involved in ECM, muscle contraction, and metabolism were affected in HCM.

**Fig. 3:**
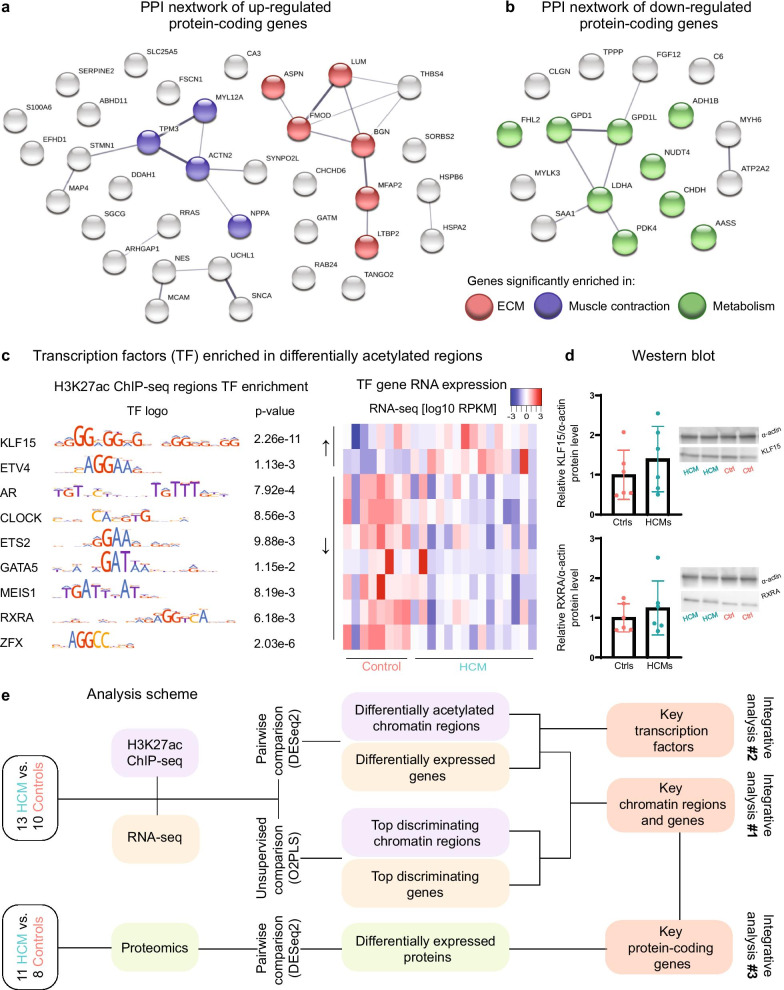
**a** An overview of protein–protein interactions of up-regulated proteins in HCM versus control hearts. Each node represents an individual protein. Nodes highlighted in red are involved in the top enriched pathway in extracellular matrix organization (ECM). Nodes highlighted in blue are involved in the top enriched pathway in muscle contraction. **b** An overview of protein–protein interactions of down-regulated proteins in HCM versus control hearts. Each node represents an individual protein. Nodes highlighted in green are involved in the top enriched pathway in metabolism. **c** Transcription factors enriched in differentially acetylated chromatin regions and their corresponding *P* values (Fisher test) are shown on the left, and their mRNA expression levels among all samples are shown in the heatmap on the right. RPKM: reads per kilobase million. **d** Western blot data showing the protein levels of KLF15 and RXRA were comparable between HCM and control hearts. **e** A schematic overview showing all analysis steps in histone acetylome, transcriptome, and proteome data used in this study

Consistently, protein-coding genes (36 up-regulated and 17 down-regulated ones) were enriched for comparable biological processes and pathways as shown in previous analyses, such as ECM and muscle contraction (Additional file 14: Table S6C). We also studied protein networks among them and observed that ECM and muscle contraction were the most enriched pathways by the up-regulated protein-coding genes and metabolism was the most enriched pathway by the down-regulated protein-coding genes (Fig. 3a, b).

### Enriched transcription factor binding motifs in differentially acetylated regions

To define the actual factors that regulate altered RNA/protein expression in HCM hearts, we studied the putative transcription factors (TFs) encoded by enriched transcription factor binding motifs (TFBMs) in differentially acetylated regions by scanning through the HOCOMOCO motif database of 769 human primary and alternative binding models. We obtained 125 TFBMs in the hyperacetylated regions and 115 TFs were predicted to bind to them. In the hypoacetylated regions, 120 TFBMs were enriched and they encoded for 111 TFs. Out of those, 68 annotated TFs were enriched in both hyper- and hypo-acetylated regions (Additional file 4: Figure S4).

Notably, 2 TFs (*KLF15* and *ETV4*) that were encoded by enriched TFBMs in the hyperacetylated regions showed higher mRNA levels in HCM hearts than controls, and 7 TFs (*AR, CLOCK, ETS2, GATA5, MEIS1, RXRA,* and *ZFX*) that were encoded by enriched TFBMs in the hypoacetylated regions showed lower mRNA levels in HCM hearts than controls (Fig. 3c). These TFs are enriched for biological functions involved in muscle hypertrophy and lipid metabolism (Additional file 15: Table S7), suggesting their potential roles in regulating the upstream signaling in HCM. Next, we examined their expressions in cardiomyocytes using the public single-nuclear RNA-seq data from adult human hearts [12]. We observed that they were all expressed in ventricular cardiomyocytes (Additional file 7: Figure S7). Interestingly, *KLF15*, *AR*, *CLOCK*, *MEIS1*, and *ZFX* were more abundantly expressed than *ETV4*, *ETS2*, *GATA5*, and *RXRA*. We also examined their mRNA expressions in genome-engineered cardiomyocytes with mutated *MYBPC3* compared to the control cardiomyocytes from a published study using RNA-seq (Additional file 18: Table S10), which provided insights into the early biological changes due to mutated *MYBPC3* in cardiomyocytes [13]. Interestingly, in line with our findings obtained from the bulk level at the severe stage, the mRNA level of *ETV4* was significantly increased (*P* value = 9.56E−9) in mutant cardiomyocytes compared to the controls, whereas the mRNA level of *RXRA* was significantly decreased (*P* value = 0.001) in mutant versus control cardiomyocytes. However, they showed comparable expression levels between HCM and control hearts in the proteome data. We further examined their protein expressions using western blot and confirmed that they were not significantly changed in HCM versus control hearts (Fig. 3d). We performed additional immunofluorescence staining and examined the expression of KLF15 in human-induced pluripotent stem cell-derived cardiomyocytes (hiPSC-cardiomyocytes) with and without mutated *MYBPC3*. We observed the expression of KLF15 in hiPSC-cardiomyocytes (Fig. 6a); however, more cell lines derived from different patients and healthy individuals are needed to study whether the KLF15 signal differs between diseased and control cardiomyocytes.

A schematic overview of all analysis steps is shown in Fig. 3e.

### Genes discriminating HCM from controls show consistent changes on various levels

**Fig. 4:**
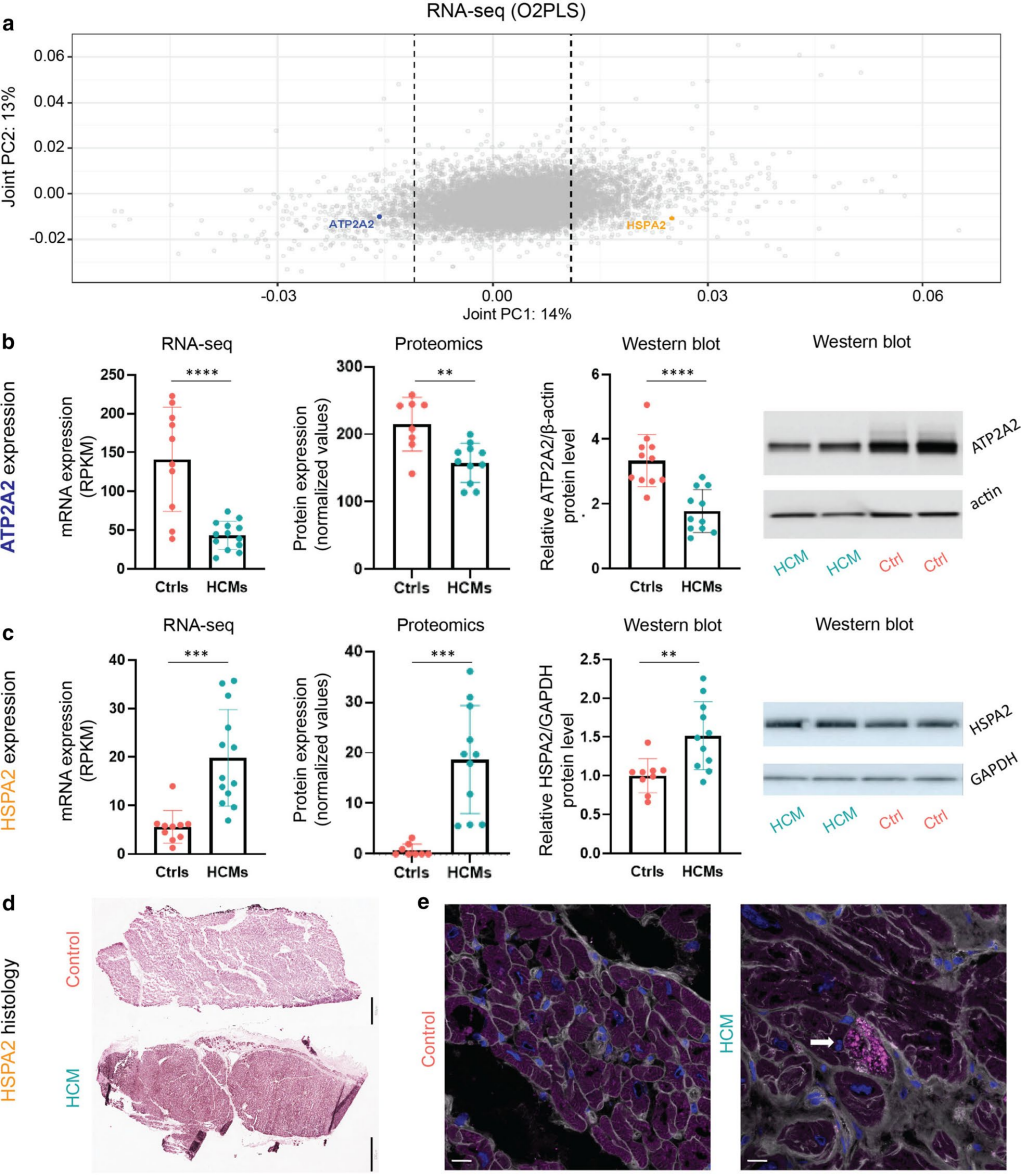
Changes of ATP2A/SERCA2a and HSPA2 at the mRNA and protein levels in HCM versus control hearts. **a** A plot of the joint component loadings of RNA-seq data showed *ATP2A2* and *HSPA2* were two major players in discriminating HCM hearts from controls. The dashed lines on both positive and negative sides indicate the cutoff threshold, with genes with a large contribution to the joint component falling outside of the dash lines. **b** ATP2A2 mRNA and protein levels in HCM and control samples at the mRNA and protein levels. **c** HSPA2 mRNA and protein levels expression in HCM and control samples. **: *P* < 0.01, ***: *P* < 0.001, ****: *P* < 0.0001. **d** Representative immunohistochemistry staining showing higher HSPA2 staining intensity in HCM heart as compared to control. Scale bar = 400 µm (control sample) and 800 µm (HCM sample). **e** Representative immunofluorescence staining showing HSPA2 aggregates in an HCM heart (indicated by the arrow), whereas the control shows diffuse staining of the cytoplasm without aggregates. WGA-AF488 appears in gray to visualize the cell membrane, and DAPI appears in blue to visualize the nuclei. Scale bar = 16 µm

We further selected an example of one down-regulated and one up-regulated gene and examined them in more detail to demonstrate the strength of our integrative omic analysis. *ATP2A2*, one protein-coding gene from the overlapping candidates, encodes the sarcoplasmic reticulum Ca2+ -ATPase pump SERCA2a and plays a critical role in the regulation of calcium handling [14]. We identified *ATP2A2* as one of the major candidates in discriminating HCM hearts from controls in our integrated H3K27ac ChIP-seq and RNA-seq analysis (Fig. 4a). Besides, its mRNA and protein levels were significantly lower in HCM versus control hearts in the pairwise comparison, and the suppressed protein level was also validated using western blot (Fig. 4b).

*HSPA2*, another protein-coding gene from the overlapping candidates, is involved in protein quality control [15]. We demonstrated that HSPA2 acts as another key player in discriminating HCM hearts from controls (Fig. 4a). Its mRNA and protein levels were significantly higher in HCM versus control hearts in the pairwise comparison, and the enhanced protein level was also validated using western blot (Fig. 4c). The upstream regulatory region of *HSPA2* also showed a higher acetylation level in HCM versus control hearts (Table 1). Moreover, we showed a profound HSPA2 intensity in HCM compared to the control heart using the immunohistochemistry staining at low magnification (Fig. 4d). Next, we employed immunofluorescence staining at high magnification and observed occasional HSPA2 aggregates in cardiomyocytes from the HCM heart, whereas no HSPA2 aggregates were shown in the control heart (Fig. 4e).

### Allelic imbalance of *MYBPC3* in HCM hearts is observed at both DNA and RNA levels

To further explore the potential of the produced data, we investigated the contribution of both *MYBPC3* alleles in the sequencing datasets. In the patient cohort, three heterozygous truncating mutations were present in *MYBPC3*, namely c.2373dupG (*n* = 5), c.2827C > T (*n* = 6), and c.927-2A > G (*n* = 2). We observed that the average acetylation ratio of *MYBPC3* with c.2373dupG, c.2827C > T, and c.927-2A > G mutation to wildtype allele was 50%, 25%, and 66.6%, respectively (Additional file 5: Figure S5A). The average mRNA expression ratio of *MYBPC3* with c.2373dupG, c.2827C > T, and c.927-2A > G mutation to wildtype allele was 6.7%, 19.7%, and 43.4%, respectively (Additional file 5: Figure S5B). The acetylation and mRNA levels of three mutations were not observed in control hearts (Additional file 5: Figure S5C and S5D). It has to be noted that it is not possible to effectively distinguish between wildtype and mutant alleles in the proteomics data.

### Cellular fraction sub-analysis of bulk cardiac tissue transcriptome indicates cardiomyocyte enrichment in a cellular-specific response in HCM

**Fig. 5:**
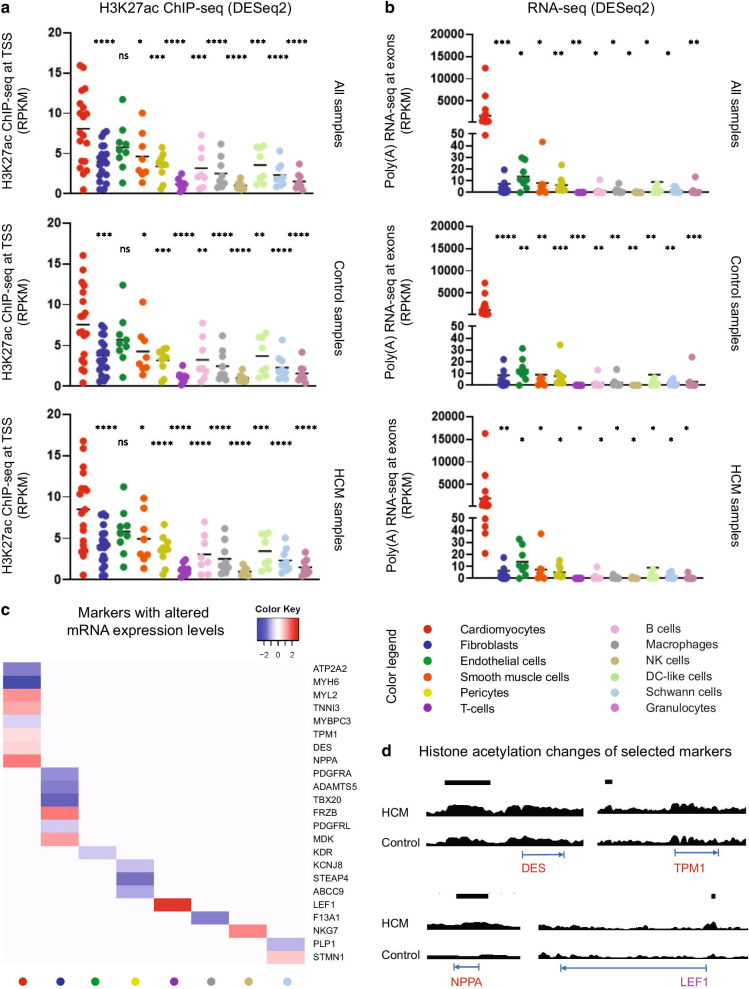
The expression of cell-type-specific markers in 12 cell populations. **a** The histone acetylation levels of 11 cell-type-specific markers. Each dot represents the average acetylation value of each marker for all samples or only control or HCM samples. One-way ANOVA was used to compare the mean of cardiomyocyte-specific markers with the mean of non-myocyte markers separately: ns (not significant), * P < 0.05, **P < 0.01, *** P < 0.001, **** P < 0.0001. **b** The mRNA expression levels of 11 cell-type-specific markers. Each dot represents the average expression value of each marker for all samples or only control or HCM samples. One-way ANOVA was used to compare the mean of cardiomyocyte-specific markers with the mean of non-myocyte markers separately: ns (not significant), *P < 0.05, ** P < 0.01, ***P < 0.001, ****P < 0.0001. **c** Heatmap showing cell-type-specific markers with significantly changed mRNA expression levels in HCM hearts compared to controls. Fold changes of these markers are depicted. Positive fold changes (red) and negative fold changes (blue) represent up-regulation and down-regulation in HCM versus control hearts, respectively. **d** Histone acetylation levels at the upstream (50 kb) of three cardiomyocytes-specific markers (red) and one T cells-specific marker (violet) were significantly changed between HCM and control hearts. Tracks of one HCM and one control heart were scaled in the UCSC genome browser (ln(x + 1): 0–10). Significantly changed upstream regions are indicated by the black bar above. The transcription start and end site of each marker are indicated by the blue arrow below

Since there are multiple cell types present in the heart samples, we collected cell-type-specific markers for cardiomyocytes and 11 non-myocyte cell types as revealed by recent single-cell studies in the heart [16, 17], ranging from 8 to 19 markers per cell type (Additional file 17: Table S9), and we examined their expression levels in our bulk sequencing data. Cardiomyocyte-specific markers showed higher histone acetylation and mRNA levels than markers of 11 non-myocyte cell types (Fig. 5a, b), regardless of health and disease. The mRNA expression of several cell-type-specific markers was significantly different between HCM and control hearts (Fig. 5c). The upstream regulatory region of three cardiomyocyte-specific markers and one T-cell-specific marker also showed significantly higher acetylation activities in HCM hearts versus controls (Fig. 5d).

#### Identified candidates supported by multi-omics data in human-induced pluripotent stem cells-derived cardiomyocytes (hiPSC-cardiomyocytes)

**Fig. 6:**
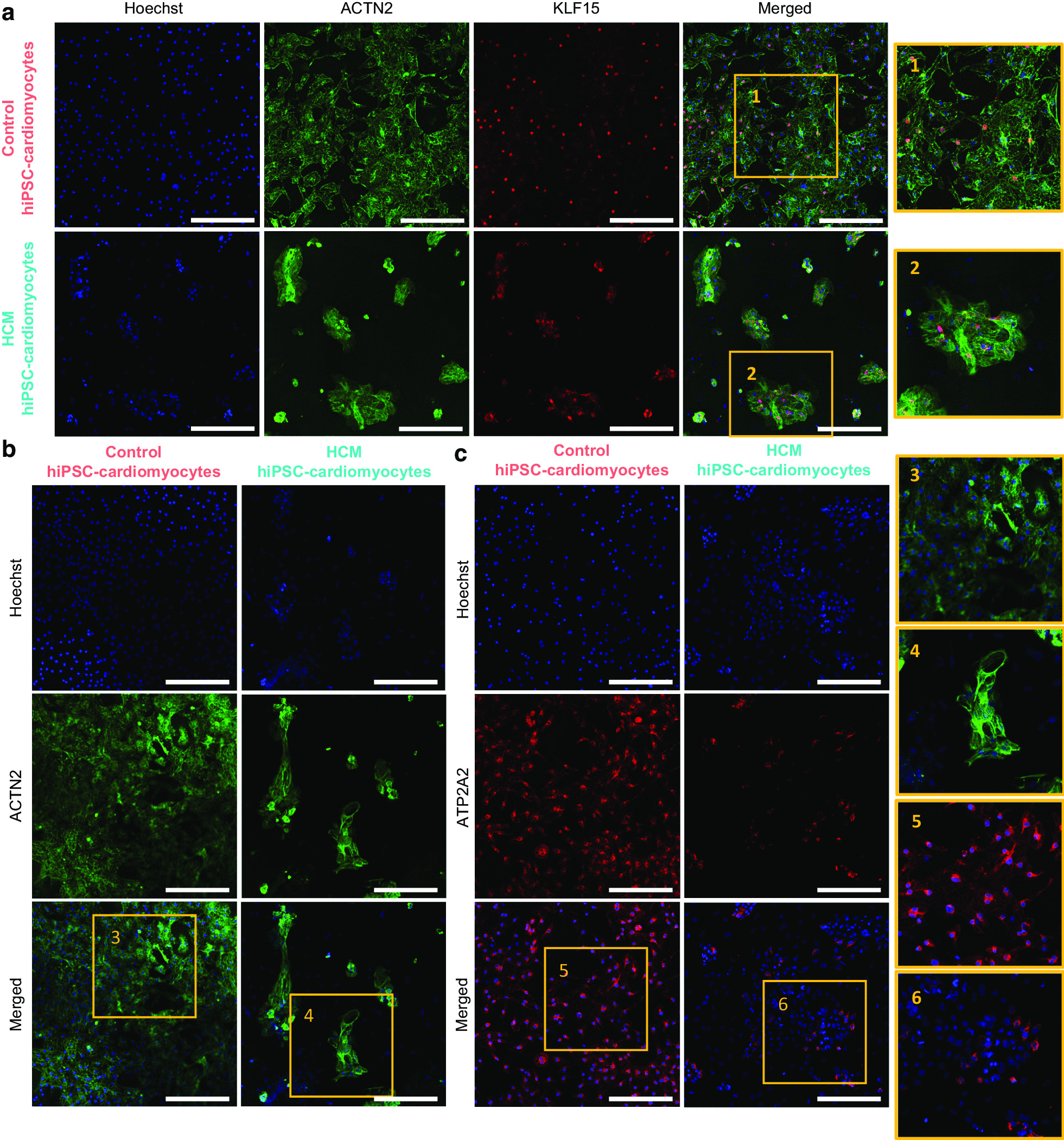
Immunofluorescence (IF) staining of selected candidates in control and HCM hiPSC-cardiomyocytes. The same amount of control and HCM hiPSC-cardiomyocytes (20,000 cells per well) were seeded to the plate and cultured for 27 days prior to the staining. **a** Representative IF staining showing the expression of KLF15 in HCM and control cardiomyocytes (KLF15: red; ACTN2: green; blue: cell nuclei). **b** Representative IF staining showing disrupted ACTN2 in HCM cardiomyocytes compared to controls (ACTN2: green; blue: cell nuclei). **c** Representative IF staining showing suppressed ATP2A2 in HCM cardiomyocytes compared to controls (ATP2A2: red; blue: cell nuclei). Nuclei were stained by Hoechst. All overview images were taken at 20 × magnification, scale bar = 100 µm. All zoomed regions were taken under Zoom Factor = 1.5

We further evaluated the presence of obtained candidates, which were supported by the acetylome, transcriptome, and/or proteome data (Table 1), using hiPSC-cardiomyocytes with and without mutated *MYBPC3*. We first employed the transcriptome profile comparing the genome-engineered cardiomyocytes harboring mutated *MYBPC3* with the controls (Additional file 18: Table S10) from a published study [13]. Out of 53 candidates, 18 protein-coding genes (34%) from our bulk data also showed significantly affected mRNA levels between diseased and control cardiomyocytes (Table 1). Next, we examined the expression levels of several candidates between hiPSC-cardiomyocytes with and without mutated MYBPC3 using immunofluorescence staining, including ACTN2 and ATP2A2. Consistent with the suppressed mRNA and protein levels of ACTN2 in HCM versus control hearts, we observed that unlike the well-aligned cytoskeleton structure in control cardiomyocytes, HCM cardiomyocytes seemed to have a disrupted cytoskeleton as revealed by ACTN2 staining (Fig. 6b). Besides, we again observed the inhibited expression of ATP2A2/SERCA2a in HCM cardiomyocytes when compared with the controls (Fig. 6c). Combined, we showed that candidates screened by multi-omics approaches from the cardiac tissues also expressed in cardiomyocytes and showed similar changing direction in HCM versus control cardiomyocytes.

## Discussion

In the present study, we studied changes in histone acetylome, transcriptome, and proteome between HCM and control hearts. Integrating these multi-omics data, we present for the first time a set of DNA regions and genes that differentiate HCM from control hearts and identified 53 protein-coding genes as the major contributors. Since these comprehensive datasets consistently indicated altered biological functions involved in ECM, muscle contraction, and metabolism, we further studied and identified the upstream TFs that could play a critical regulatory role. We also observed an abundance of acetylation signals and transcripts signals from cardiomyocyte-specific markers compared to markers of 11 non-myocyte populations in the heart.

By integrating ChIP-seq, RNA-seq, and proteomics data we identified a list of deciding elements in discriminating HCM hearts from controls. Among these, *ATP2A2*/SERCA2a, the cardiomyocyte-specific marker, transports cytosolic calcium into the sarcoplasmic reticulum and subsequently regulates the contraction and relaxation of cardiomyocytes [18]. Suppressed *ATP2A2*/SERCA2a expression is associated with impaired relaxation in cardiomyocytes and contributes to diastolic dysfunction in HCM [14, 19]. Consistent with previous findings, we also showed lower mRNA and protein levels in HCM hearts compared to controls. Additionally, we employed hiPSC-derived cardiomyocytes and confirmed the suppressed ATP2A2/SERCA2a in HCM versus control cardiomyocytes.

Another element HSPA2 (heat shock-related 70 kDa protein 2), which protects cardiac integrity by correcting misfolded proteins upon stress [20], showed higher upstream acetylation level, mRNA level, and protein level in HCM hearts versus controls. Up-regulated HSPA2 protein level has been observed at both the initial stage of diseased cardiomyocytes and at the end stage of diseased heart with diastolic dysfunction [21, 22]. We previously also showed elevated HSPA2 protein level in HCM with mutations in different sarcomeric proteins when compared to controls, and it was negatively correlated with MYBPC3 peptide counts [15]. Here we further demonstrated a cardiomyocyte-specific elevation of HSPA2 accompanied by the occasional HSPA2 aggregates in the HCM heart, which may contribute to the disease progression by hampering the protein quality control system.

Interestingly, both ATP2A2/Serca2a- and HSPA2*-*mediated activities are ATP-dependent, highlighting the strength of our sequencing data that align with previous observations showing energy deficiency as a key pathomechanism in HCM development. A reduced myocardial external efficiency, the ratio between cardiac work and oxygen consumption, was observed in asymptomatic *MYBPC3* mutation carriers compared to the control hearts [23]. We also provided a review summarizing the metabolic changes in HCM hearts [24]. Isolated cardiomyocytes from HCM hearts with *MYBPC3* mutations showed a reduced super-relax state, which may result in an increased sarcomere energy utilization [25]. Likewise, hiPSC-derived cardiomyocytes with mutated *MYH7*, another secomeric gene that is often mutated in HCM patients, also showed a reduced super-relax state, an increased contractility, and a greater basal oxygen consumption rate when compared to the controls, suggesting a higher ATP demand [26]. Taken together, previous studies show mutation-mediated contractile dysfunction with associated disturbed energetics. Here, besides the enriched metabolism using the ChIP-seq, RNA-seq, and proteomic data, we also identified several TFs as potential key upstream factors in regulating metabolism in HCM hearts, such as KLF15, *AR*, and *RXRA* [27–29]. Furthermore, we confirmed the expression of KLF15, a key regulator in cardiac metabolism [30], in hiPSC-cardiomyocytes using immunofluorescence staining, highlighting the potential involvement of KLF15 in diseased cardiomyocytes. Drugs targeting energy metabolism, such as trimetazidine, have been investigated in the clinical trial in HCM patients [31]. Combined, identified TFs could be potential therapeutic targets in restoring metabolic homeostasis in HCM patients and future studies in hiPSC-cardiomyocyte models are warranted to define the mutation-mediated sequence in mitochondrial function and cellular metabolism.

Although those identified TFs showed the same changing direction at the acetylation and mRNA levels in HCM versus control hearts, their protein levels were comparable between HCM and control hearts. Similarly, some of those identified key protein-coding genes, which changed in the same direction at the mRNA and protein levels, showed comparable acetylation levels between HCM and control hearts. These findings indicate that the central dogma of cellular information transition from DNA, RNA, to protein is oversimplified [32]. Numerous factors, such as the post-translational modifications on gene expression and TF activity, mRNA degradation, and protein degradation [33–36], could lead to a poor correlation between DNA, mRNA, and protein expressions. Additionally, both H3K27ac ChIP-seq and RNA-seq data were generated from the same 13 HCM and 10 control samples, whereas only 11 out of 13 HCM samples were included in the proteomics analysis in comparison with 8 different control samples. Thus, different donor samples and group sizes might obscure the integrative analysis of the multi-omics data. Therefore, it is important to find an optimal way to integrate multi-omics data rather than a simple correlative. The discovery of new types of machinery beyond the central dogma is also needed in future studies.

Previous studies from our group and others showed around a 30% ratio of mutant to wildtype *MYH7*- and *MYBPC3-*alleles in HCM patients at the mRNA and the protein levels, respectively [37, 38]. In this study, we present the imbalanced mRNA expression of three truncating mutations in *MYBPC3*. Notably, we showed for the first time the presence of such an imbalance at the DNA level by evaluating allele-specific histone acetylation signals. The mutant/wildtype expression ratio differs between truncating mutations and between DNA and mRNA levels, suggesting mutation-specific imbalance and additional machinery between DNA and mRNA. It is important to point out that H3K27ac ChIP-seq is sensitive in capturing upstream regulatory regions of a gene and is restored in covering the core gene body [39], and the allelic expression based on the histone acetylation level could be limited by the nature of the technique. Nevertheless, this finding highlights another utilization of omics data in detecting allelic imbalance for genetic diseases.

As the cell composition changes in diseased hearts, we investigated cell-type-specific signals from cardiomyocytes and 11 non-myocyte cell types and observed more ChIP-seq signals and transcripts in cardiomyocyte-specific markers than all non-myocyte markers, suggesting a considerable portion of cardiomyocytes-derived signals in the bulk sequencing data. Gene set enrichment analysis showed enriched pathways in ECM, which is in line with previous reports indicating fibrosis as a hallmark of HCM [40]. Apart from fibroblasts, cardiomyocytes also express ECM-related genes, including two fibroblast-specific markers (*DKK3* and *ABI3BP*) [41]. Because cell-type-specific markers might also be expressed by other cell types and the limitation of capturing pure cell populations from snap-frozen tissues without damaging the DNA and RNA integrity, we can only speculate that the majority of the signals were likely obtained from cardiomyocytes and it remains a challenge to separate them in bulk sequencing. Additionally, we used cell-type-specific markers obtained from the murine models [16, 17], which may not translate one-to-one across species. Therefore, future studies are required to validate the cellular-specific response in HCM by isolating single-cell populations from human cardiac tissues.

In conclusion, our study presents detailed information in HCM hearts with truncating *MYBPC3* mutations. These data showed altered ECM, muscle contraction, and metabolism in HCM. Integrative analyses further identified a subset of protein-coding genes and upstream TFs that could drive these pathophysiological mechanisms and serve as promising diagnostic and/or therapeutic targets. We also showed a considerable amount of cardiomyocyte-derived signals compared to non-myocyte cell types, providing cardiomyocyte-specific insights to better understand HCM in future studies.

## Materials and methods

### Human cardiac samples

The study protocol was approved by the local medical ethics review committees, including the Biobank Research Ethics Committee of University Medical Center Utrecht (protocol number 12/387), the Local Ethics Committee of the Erasmus MC (2010-409), the Washington University School of Medicine Ethics Committee (Institutional Review Board), and the Sydney Heart Bank (HREC Univ. Sydney 2012/030). Since septal thickening is a typical feature of HCM, septal myectomy is commonly performed to relieve left ventricular outflow tract obstruction in HCM patients [2, 42, 43]. Therefore, we collected cardiac tissue of the interventricular septum of 13 HCM patients. Genetic analyses of all patients revealed three truncating pathogenic heterozygous mutations in *MYBPC3*, namely c.2373dupG in 5 HCM patients, c.2827C > T in 6 patients, and c927-2A > G in 2 HCM patients. Control tissues of 18 non-failing donors were obtained from the Biobank of University Medical Center Utrecht, the Washington University School of Medicine, and the Sydney Heart Bank. Informed consent was obtained from each patient prior to surgery or was waived by the ethics committee when acquiring informed consent was not possible due to the death of the donor. Samples were collected and snap-frozen in liquid nitrogen and stored at − 80 ˚C up until analysis. Detailed clinical characteristics are shown in Additional file 9: Table S1, and an overview of samples included in the following experiments is shown in Fig. 1a.

### H3K27ac chromatin immunoprecipitation and sequencing (ChIP-seq)

We performed chromatin immunoprecipitation and sequencing (ChIP-seq) using the H3K27ac mark to study the differences of the histone acetylome between patient and control samples as previously described [10]. To study the differences of the histone acetylome between patient and control samples, we performed chromatin immunoprecipitation and sequencing (ChIP-seq) using the H3K27ac mark. Briefly, all cardiac samples were sectioned at a thickness of 10 µm, and chromatin was isolated using the MAGnify™ Chromatin Immunoprecipitation System kit (Life Technologies) according to the manufacturer’s instructions. The anti-histone H3K27ac antibody (ab4729, Abcam) was used for immunoprecipitation. Captured DNA was purified using the ChIP DNA Clean & Concentrator kit (Zymo Research). Libraries were prepared using the NEXTflex™ Rapid DNA Sequencing Kit (Bioo Scientific). Samples were PCR amplified, checked for the proper size range and absence of adaptor dimers on a 2% agarose gel, and barcoded libraries were sequenced 75-bp single end on an Illumina NextSeq500 sequencer. Sequencing reads were mapped against the reference genome (hg19 assembly, NCBI37) using the BWA package (mem –t 7 –c 100 –M –R)1. Multiple reads mapping to the same location and strand were collapsed to a single read, and only uniquely placed reads were used for peak calling. Peaks/regions were called using Cisgenome 2.02 (–e 150 -maxgap 200 –minlen 200). Region coordinates from all samples were stretched to at least 2000 base pairs and collapsed into a single common list. Overlapping regions were merged based on their coordinates. Only regions supported by at least 2 independent datasets were further analyzed. Autosomal sequencing reads from each ChIP-seq library were overlapped back with the common region list to set the H3K27ac occupancy for every region-sample pair. Differentially acetylated regions between HCM and control hearts were identified using DESeq2 under the default setting in the Galaxy environment [44].

### Annotating genes in the vicinity of differentially acetylated regions

Region-to-gene annotation was performed to study potentially affected genes in the vicinity of DNA regions with altered acetylation levels in HCM hearts when compared with controls. Differentially acetylated regions located within either ± 5 kb or ± 50 kb window from the transcription start site (TSS) of all genes were obtained, and the nearest genes of these regions were collected.

### Predicting transcription factor binding motifs in differentially acetylated regions

To study the putative upstream signaling, we studied the enriched transcription factor binding motifs (TFBMs) by differentially acetylated regions and motifs-encoded transcription factors (TFs). DNAse I hypersensitivity regions in human cardiac samples, which play a key role in transcription factor footprinting [45], were collected from the ENCODE project and overlapped with differentially acetylated regions in this study [46]. Overlapping DNA sequences between differentially acetylated regions and DNAse I hypersensitivity regions were used to studying the enriched transcription factor binding motifs and motifs-encoded TFs using MEME Suite AME tool (HOCOMOCO Human v11 Full, average odds scoring method, and Fisher’s exact test) [47].

### RNA sequencing

We also performed RNA sequencing (RNA-seq) and obtained the transcriptome landscapes in all samples. Briefly, RNA was isolated using the RNeasy Micro Kit (Qiagen) or ISOLATE II RNA Mini Kit (Bioline) according to the manufacturer's instructions. Sample quality was assessed using the 2100 Bioanalyzer with an RNA 6000 Pico Kit (Agilent), and sample quantity was measured using Qubit Fluorometer with an HS RNA Assay (Thermo Fisher). Afterward, libraries were prepared using the NEXTflex™ Rapid RNA-seq Kit (Bioo Scientific) and sequenced by the Nextseq500 platform (Illumina). Sequenced reads were aligned to the human reference genome GRCh37 using STAR v2.4.2a [48]. Reads per kilobase million reads sequenced (RPKMs) were calculated with edgeR’s RPKM function [49]. To identify a list of differentially expressed genes between HCM and control hearts at *P*_adj_ < 0.05, we employed d DESeq2 to process all the raw counts per sample per group in the Galaxy environment [44].

### Allele-specific expression of *MYBPC3* in ChIP-seq and RNA-seq data

RNA-seq and ChIP-seq reads were processed with TrimGalore (version 0.6.5) to detect and clip off adaptor sequences, reads with a remaining length of 20 or more nucleotides, and an average read quality *q* > 20 were selected. The reads were aligned to the human genome (GRCh37) with transcript annotation from Ensembl (version 74) using the STAR aligner (version 2.7.1a). To facilitate the equal alignment of reads from wildtype and mutant alleles, alignments were made without clipping off end sequences (STAR option alignEndsType set to EndToEnd), and only best scoring alignments were selected (STAR option outSAMprimaryFlag set to AllBestScore). Alignments were selected to remove duplicated reads by an in-house HTSeq based python (version 2.7.10) script [50]. Reads that aligned to the same genomic interval and that aligned with identical bases to the two non-indel *MYBPC3* SNPs (rs397516082 and rs387907267) of interest, were considered duplicates. ChIP-seq reads matching to one of the three SNPs of interest were assigned to the wildtype or mutant allele based on the following rules: for the single-base polymorphic SNPs (rs397516082 and rs387907267), based on having the wildtype or mutant nucleotide aligned to the SNP position; for the indel SNP (rs397515963) based on having the exact wildtype or mutant sequence of the SNP with 10 surrounding bases in a read aligned to the SNP. In addition, ChIP-seq reads with wildtype or mutant assignment were required to have > 90% of the bases aligned to the genome. For RNA-seq reads, the assignment to be wildtype or mutant allele derived was complicated by changed splice patterns for two of the three SNPs. For rs387907267 (c.2827C > T) that has no changed splice patterns, reads were classified based on having the wildtype or mutant nucleotide aligned to the SNP position. For rs397515963 (c.2373dupG) that disrupts the correct splicing of intron 23, reads covering the splice junction with the correct intron spliced out were counted as wildtype reads, whereas reads using either the correct intron donor or acceptor site, but not both, were counted as mutant reads. For rs397516082 (c.927-2A > G) that disrupts the correct splicing of intron 11, reads covering the splice junction with the correct intron spliced out were counted as wildtype reads, whereas reads that were running through the intron 11–exon 12 splice site and into the SNP position were counted as wildtype or mutant based on having the wildtype or mutant nucleotide aligned to the SNP position.

### Integrating ChIP-seq and RNA-seq data with Two-way Orthogonal Partial Least Squares

To find common parts between RNA-seq and ChIP-seq data simultaneously across all genes and regions, a data integration approach is considered using two-way orthogonal partial least squares (O2PLS) [51]. O2PLS decomposes both RNA-seq and ChIP-seq datasets into joint, omic-specific, and residual parts. The joint subspaces contain variations that are correlated to one another. The joint principal components (JPCs) that span the joint subspaces are obtained by finding linear combinations of genes and regions that maximize the covariation. Omic-specific subspaces capture variation unrelated to another omics dataset, enabling JPCs to better estimate the underlying system. Here, the rows of the ChIP-seq and RNA-seq data should represent the same samples. Note that O2PLS uses all genes and regions in the datasets and does not rely on prior information about the position or function of these features. Furthermore, O2PLS is unsupervised, and its algorithm is implemented in the OmicsPLS R package and freely available from CRAN [52]. Prior to the analysis, genes with expression lower than 10 counts in at least 22 samples were removed. Samples in both datasets were matched, and 23 overlapping samples were retained. The expression data are normalized. Both datasets were log-transformed and quantile normalized across samples. The dimensionality of the preprocessed datasets is 23 by 15,882 (RNA-seq) and 23 by 33,642 (ChIP-seq).

### Proteomics

We performed proteomics using cardiac samples from the same patients (*n* = 11) and 8 non-failing donor samples from the Sydney Heart Bank (Additional file 9: Table S1). Briefly, proteins were loaded to a 4–12% NuPAGE Novex Bis–Tris 1.5 mm mini gel (Invitrogen) for separation, followed by fixation (50% ethanol and 3% phosphoric acid) and staining using 0.1% Coomassie brilliant blue G-250 solution. In-gel digestion was performed, and the samples were concentrated in a vacuum centrifuge as described previously [53]. Nano-LC–MS/MS was performed as described previously [54]. Briefly, separated peptides were separated using an Ultimate 3000 Nano LC–MS/MS system (Dionex LC-Packings, Amsterdam, The Netherlands) and trapped. Eluting peptides were ionized into a Q Exactive mass spectrometer (Thermo Fisher, Bremen, Germany), and intact masses were measured in the orbitrap. Among all, the top 10 peptide signals were selected and analyzed using the MS/MS. MS/MS spectra were searched against a Uniprot human reference proteome FASTA file (Swissprot_2017_03_human_canonical_and_isoform.fasta, 42,161 entries) using MaxQuant version 1.5.4.1. Differentially expressed proteins between HCM and control hearts at *P* < 0.05 were identified using DESeq2 [44].

### Functional enrichment analysis

*Gene ontology (GO) enrichment analysis*: To study the enriched biological functions, GO enrichment analysis was performed using the ToppFun ToppGene Suite under the default settings (FDR correction, *P* value cutoff of 0.05, and gene limits between 1 and 2,000) [55].

*Gene set enrichment analysis (GSEA)*: Established gene sets involved in the most enriched biological functions in HCM versus control hearts were collected from Molecular Signature Database v7.1 (Additional file 16: Table S8) [56, 57]. Each gene set per biological function was studied for its positive or negative correlation with genes annotated from the altered acetylated levels in the ChIP-seq data, genes with altered mRNA levels in the RNA-seq data, and protein-coding genes with altered protein levels in the proteomic data in this study under the following setting: Number of permutations: 1,000; Phenotype labels: up_versus_down; Collapses dataset to gene symbols: false; Permutation type: gene_set; Enrichment statistics: weighted; Metric for ranking genes: Signal2Noise; Gene list sorting mode: real; Gene list ordering mode: descending; Max size: 500; Min size: 1.

*Protein–protein interaction (PPI) networks*: Protein networks were performed using the STRING Version 11.0 under the following settings: the meaning of network edges: confident, minimum required interaction score: medium confidence (0.400) [58].

#### Human-induced pluripotent stem cells-derived cardiomyocytes (hiPSC-cardiomyocytes)

Clones from one control hiPSC line and one hiPSC line derived from an HCM patient with mutated *MYBPC3* were differentiated to cardiomyocytes as previously described [59]. The contractile function of differentiated control and HCM cardiomyocytes was examined as previously described [60]. Cells were seeded at the density of 20,000 cells per well to the 96-well plate. After culturing for 27 days, cardiomyocytes were fixed and stained for interested proteins.

#### Western blot

Western blot was performed as described previously [15]. Primary antibodies, including mouse anti-HSPA2 (heat shock-related 70 kDa protein 2, 66291-1, Proteintech Group), mouse anti-GAPDH (10R-G109a, Fitzgerald Industries International), anti-ATP2A2 (sarcoplasmic reticulum Ca2 + -ATPase 2, also known as SERCA2), anti-KLF15 (AV32587, Sigma-Aldrich), anti-RXRA (ab125001, Abcam), and anti-β-actin, were used.

#### Immunohistochemistry and immunofluorescence (IF) staining

Tissue sections were thawed and left at RT for 20 min inside a closed box. Then the sections were treated with peroxidase blocking solution (3% H2O2 in MeOH) and blocked with 1% BSA. HSPA2 (Anti-HSPA2 rabbit-Polyclonal antibody Prestige Antibodies HPA000798) primary antibodies were incubated for 1 h at RT (1:100 for IF and 1:200 for IHC). Sections were washed and incubated with the secondary antibodies for 30 min. Vector Vectastain Universal Elite ABC Kit (PK-6200) was used to enhance the brightfield staining (IHC) and WGA (for cell membranes) and DAPI (nuclei) were added to counterstain fluorescence staining. The brightfield slides were washed, treated with DAB, counterstained with Mayers Hematoxylin, dehydrated and mounted with DPX. Fluorescence staining was mounted with Mowiol. Images were acquired with the Vectra Polaris Scanner (brightfield/IHC) or Confocal Nikon A1 (IF). The analysis was performed with QuPath, Fiji, and NIS Nikon software.

HiPSC-cardiomyocytes were fixed using 4% paraformaldehyde solution for 10 min at room temperature and incubated overnight with the primary antibodies, including ACTN2 (A7811, Sigma-Aldrich) and ATP2A2 (MA3-910, Thermo Fisher Scientific). Afterward, cells were washed three times with PBS and incubated for 1 h with Hoechst 33,342 and the secondary antibodies (Invitrogen), including Alexa Fluor 488 and Alexa Fluor 568. Images were taken by Leica confocal microscope at 20× magnification.

## Supplementary information

**Additional file 1: Figure S1** Examples of differentially acetylated regions between HCM and control hearts.

**Additional file 2: Figure S2** (**A**) Protein-protein interactions between up-regulated proteins in HCM versus control hearts. (**B**) Protein-protein interactions between down-regulated proteins in HCM versus control hearts

**Additional file 3: Figure S3** (**A**) Gene set enrichment report showing the correlation between extracellular matrix (EC)-related genes and genes annotated to differentially acetylated regions. (**B**) Gene set enrichment report showing the correlation between ECM-related genes and differentially expressed genes. (**C**) Gene set enrichment report showing the correlation between ECM-related genes and genes encoding differentially expressed proteins. (**D**) Gene set enrichment report showing the correlation between cardiac muscle contraction-related genes and genes annotated to differentially acetylated regions. (**E**) Gene set enrichment report showing the correlation between cardiac muscle contraction-related genes and differentially expressed genes. (**F**) Gene set enrichment report showing the correlation between cardiac muscle contraction-related genes and genes encoding differentially expressed proteins. (**G**) Gene set enrichment report showing the correlation between fatty acid metabolism-related genes and genes annotated to differentially acetylated regions. (**H**) Gene set enrichment report showing the correlation between fatty acid metabolism-related genes and differentially expressed genes. (**I**) Gene set enrichment report showing the correlation between fatty acid metabolism-related genes and genes encoding differentially expressed proteins

**Additional file 4: Figure S4** (**A**) Enriched transcription factor binding motifs in the hyperacetylated regions in HCM versus control hearts. (**B**) Enriched transcription factor binding motifs in the hypoacetylated regions in HCM versus control hearts

**Additional file 5: Figure S5** The acetylation and mRNA levels of the wildtype and mutant *MYBPC3* alleles in all samples

**Additional file 6: Figure S6** Principal component analysis (PCA) plot showing the clustering between control samples and HCM with different truncating mutations in the *MYBPC3* gene

**Additional file 7: Figure S7** The expression levels of obtained transcription factors in ventricular cardiomyocytes using published single-cell sequencing data

**Additional file 8: Figure S8** The expression levels of cardiomyocyte-specific markers in ventricular cardiomyocytes using published single-cell sequencing data

**Additional file 9: Table S1** An overview of the clinical characteristics in all cardiac samples

**Additional file 10: Table S2** (**A**) Differentially acetylated regions between HCM and control hearts from the H3K27ac ChIP-seq data. (**B**) Genes annotated to differentially acetylated regions using the 5 kb window of the transcription start site. (**C**) Genes annotated to differentially acetylated regions using the 50 kb window of the transcription start site. (**D**) Enriched GO terms and pathways by genes annotated to the hyperacetylated regions (50 kb window). (**E**) Enriched GO terms and pathways by genes annotated to the hypoacetylated regions (50 kb window)

**Additional file 11: Table S3** (**A**) Differentially expressed genes between HCM and control hearts from the RNA-seq data. (**B**) Enriched GO terms and pathways by up-regulated genes in HCM versus control hearts. (**C**) Enriched GO terms and pathways by down-regulated genes in HCM versus control hearts

**Additional file 12: Table S4** (**A**) Differentially expressed proteins between HCM and control hearts from the proteomics data. (**B**) Enriched GO terms and pathways by up-regulated proteins in HCM versus control hearts. (**C**) Enriched GO terms and pathways by down-regulated proteins in HCM versus control hearts

**Additional file 13: Table S5** (**A**) Unsupervised analysis (O2PLS) ranking DNA regions that contributed to the separation between HCM and control hearts. (**B**) Unsupervised analysis (O2PLS) ranking genes that contributed to the separation between HCM and control hearts

**Additional file 14: Table S6** (**A**) The overlapping genes between differentially expressed genes using DESeq2 and the top 2,000 genes that discriminate HCM from control hearts using O2PLS. (**B**) Enriched GO terms and pathways by the overlapping genes. (**C**) Enriched GO terms and pathways by a subset of the overlapping genes, which showed the same changing direction at their protein levels in HCM hearts when compared with controls

**Additional file 15: Table S7** Enrichment results of identified transcription factors that were supported by both ChIP-seq and RNA-seq data in HCM versus control hearts

**Additional file 16: Table S8** Gene sets collected from the Molecular Signature Databases

**Additional file 17: Table S9** The acetylation and mRNA expression levels of included cell-type-specific markers

**Additional file 18: Table S10** A published transcriptome data comparing genome-engineered cardiomyocytes with and without mutated *MYBPC3* using RNA sequencing

## Data Availability

All relevant data are available within the article and the supplementary files. Because of the sensitive nature of the data collected for this study (13 patient samples and 10 control samples), requests to access the raw sequencing dataset from qualified researchers trained in human subject confidentiality protocols may be sent to the corresponding authors. It is important to note that processed RNA-seq and H3K27ac ChIP-seq data of 11 patient samples and 7 control samples are published in a GWAS study [7] and available in Additional file 11: Table S3 and Additional file 13: Table S5, respectively. Raw proteomics data within the article can be found at the ProteomeXchange Consortium via the PRIDE partner repository with the dataset identifier PXD012467 [61].

## Abbreviations

HCM: hypertrophic cardiomyopathy
NGS: next-generation sequencing
ChIP-seq: chromatin immunoprecipitation and sequencing
RNA-seq: RNA sequencing
DARs: differentially acetylated regions
TSS: transcription start site
TF: transcription factor
TFBMs: transcription factor binding motifs
RPKMs: reads per kilobase million reads sequenced
O2PLS: two-way orthogonal partial least squares
JPCs: joint principal components
GO: gene ontology
GSEA: gene set enrichment analysis
PPI: protein–protein interaction
IF: immunofluorescence
IHC: immunohistochemistry
ECM: extracellular matrix
