# Supplementary figures and images for: Multi-omics integration identifies key upstream regulators of pathomechanisms in hypertrophic cardiomyopathy due to truncating MYBPC3 mutations
Source: Clin Epigenetics. 2021 Mar 23;13:61. doi: 10.1186/s13148-021-01043-3 (PMC7989210; doi:10.1186/s13148-021-01043-3)

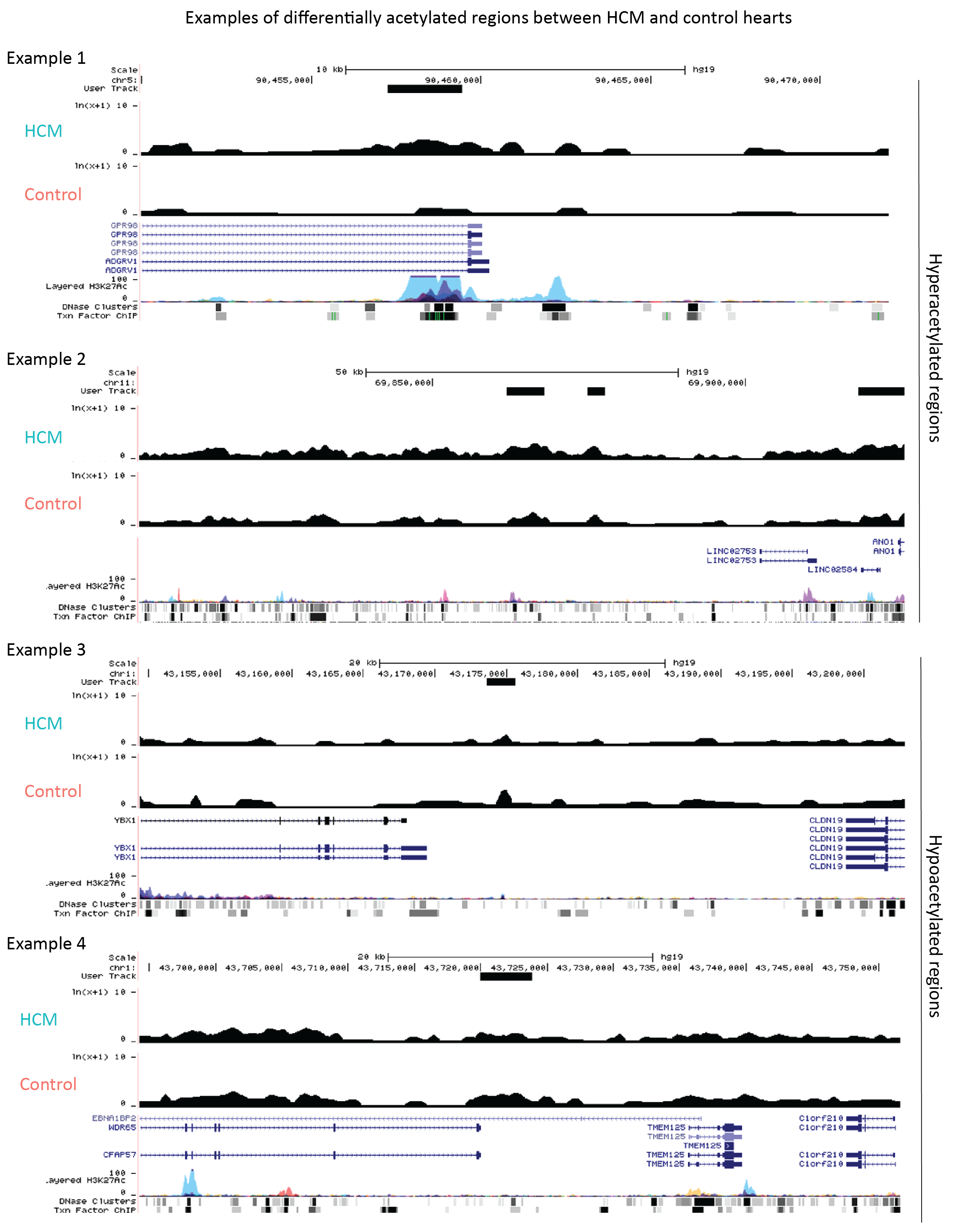

Supplement: Supplementary file 1 — Additional file 1: Figure S1 Examples of differentially acetylated regions between HCM and control hearts. [file 13148_2021_1043_MOESM1_ESM.png]

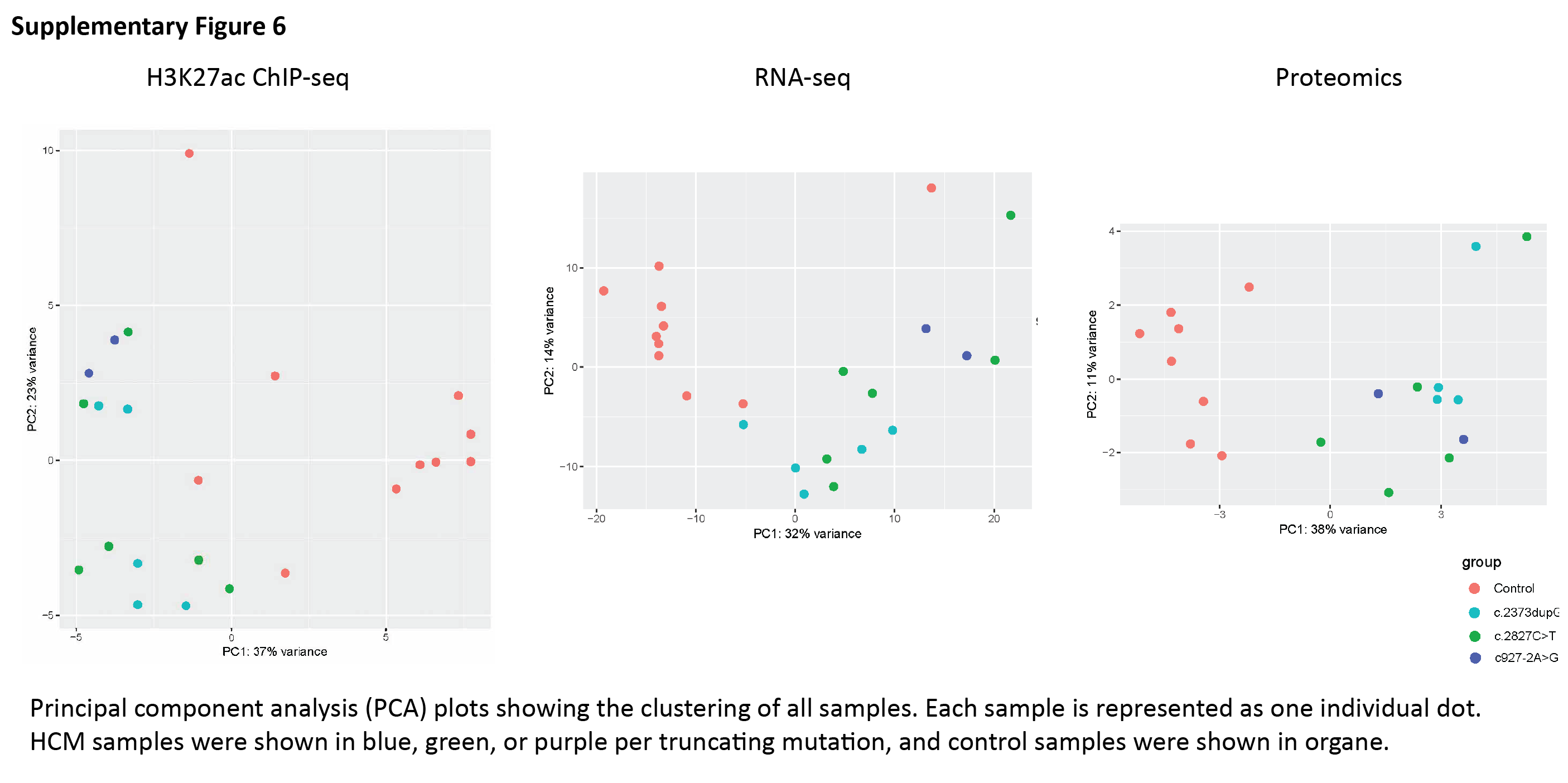

Supplement: Supplementary file 6 — Additional file 6: Figure S6 Principal component analysis (PCA) plot showing the clustering between control samples and HCM with different truncating mutations in the MYBPC3 gene [file 13148_2021_1043_MOESM6_ESM.png]

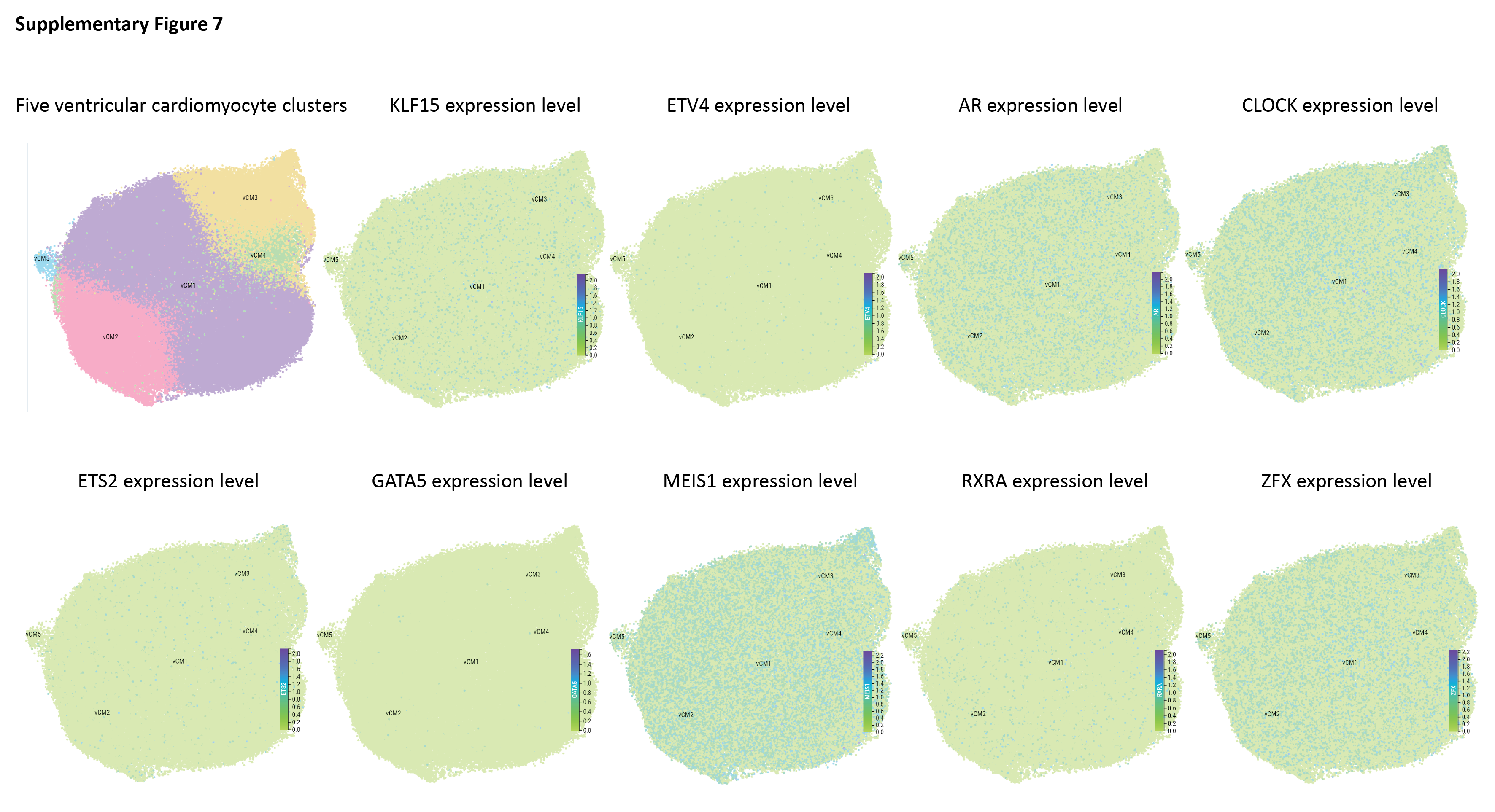

Supplement: Supplementary file 7 — Additional file 7: Figure S7 The expression levels of obtained transcription factors in ventricular cardiomyocytes using published single-cell sequencing data [file 13148_2021_1043_MOESM7_ESM.png]

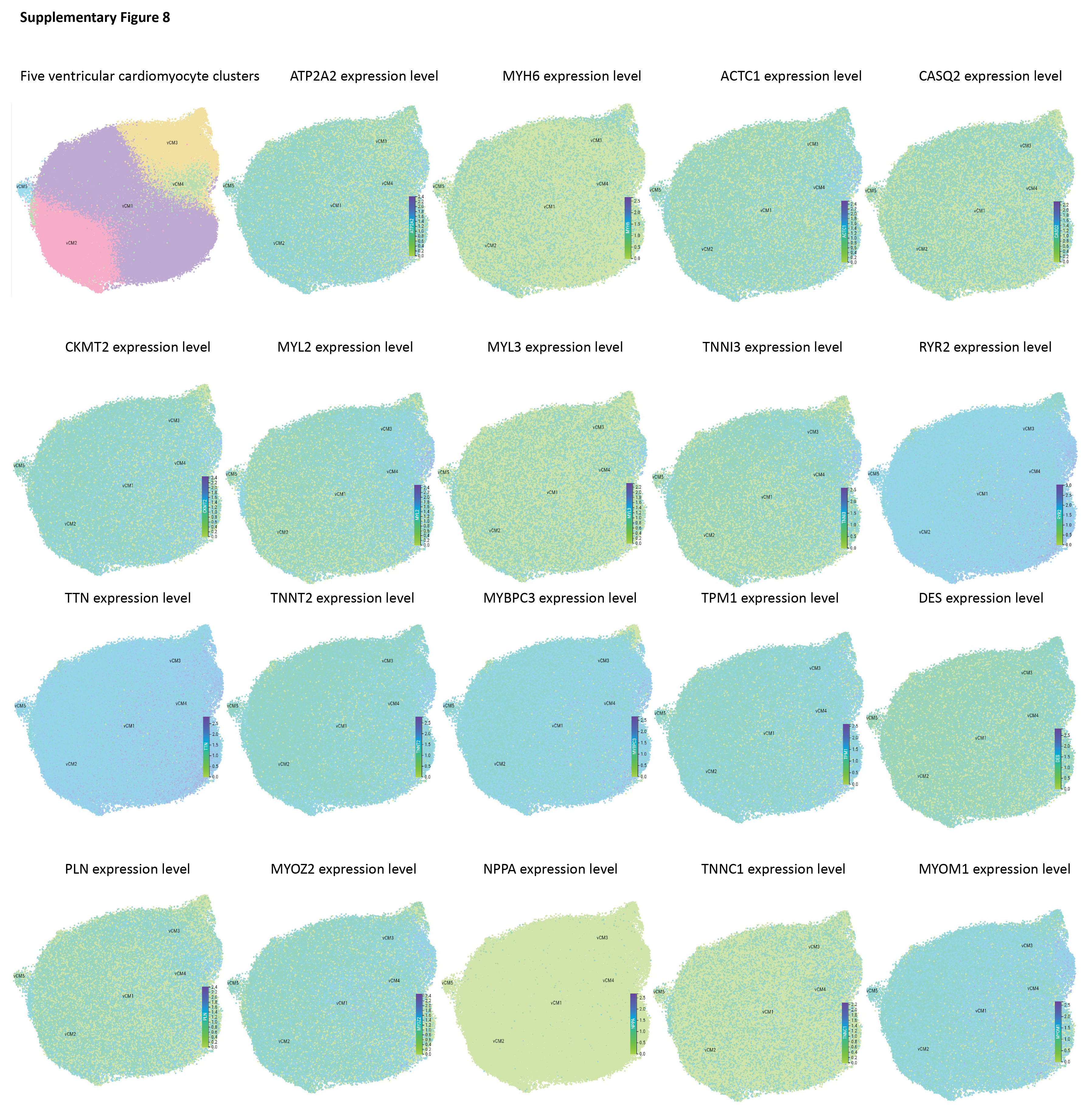

Supplement: Supplementary file 8 — Additional file 8: Figure S8 The expression levels of cardiomyocyte-specific markers in ventricular cardiomyocytes using published single-cell sequencing data [file 13148_2021_1043_MOESM8_ESM.jpg]
