# Supplementary material for: Multi-omics integration identifies key upstream regulators of pathomechanisms in hypertrophic cardiomyopathy due to truncating MYBPC3 mutations
Source: Clin Epigenetics. 2021 Mar 23;13:61. doi: 10.1186/s13148-021-01043-3 (PMC7989210; doi:10.1186/s13148-021-01043-3)
Supplement: Supplementary file 2 — Additional file 2: Figure S2 (A) Protein-protein interactions between up-regulated proteins in HCM versus control hearts. (B) Protein-protein interactions between down-regulated proteins in HCM versus control hearts [file 13148_2021_1043_MOESM2_ESM.pdf]

Supplementary Figure 1A

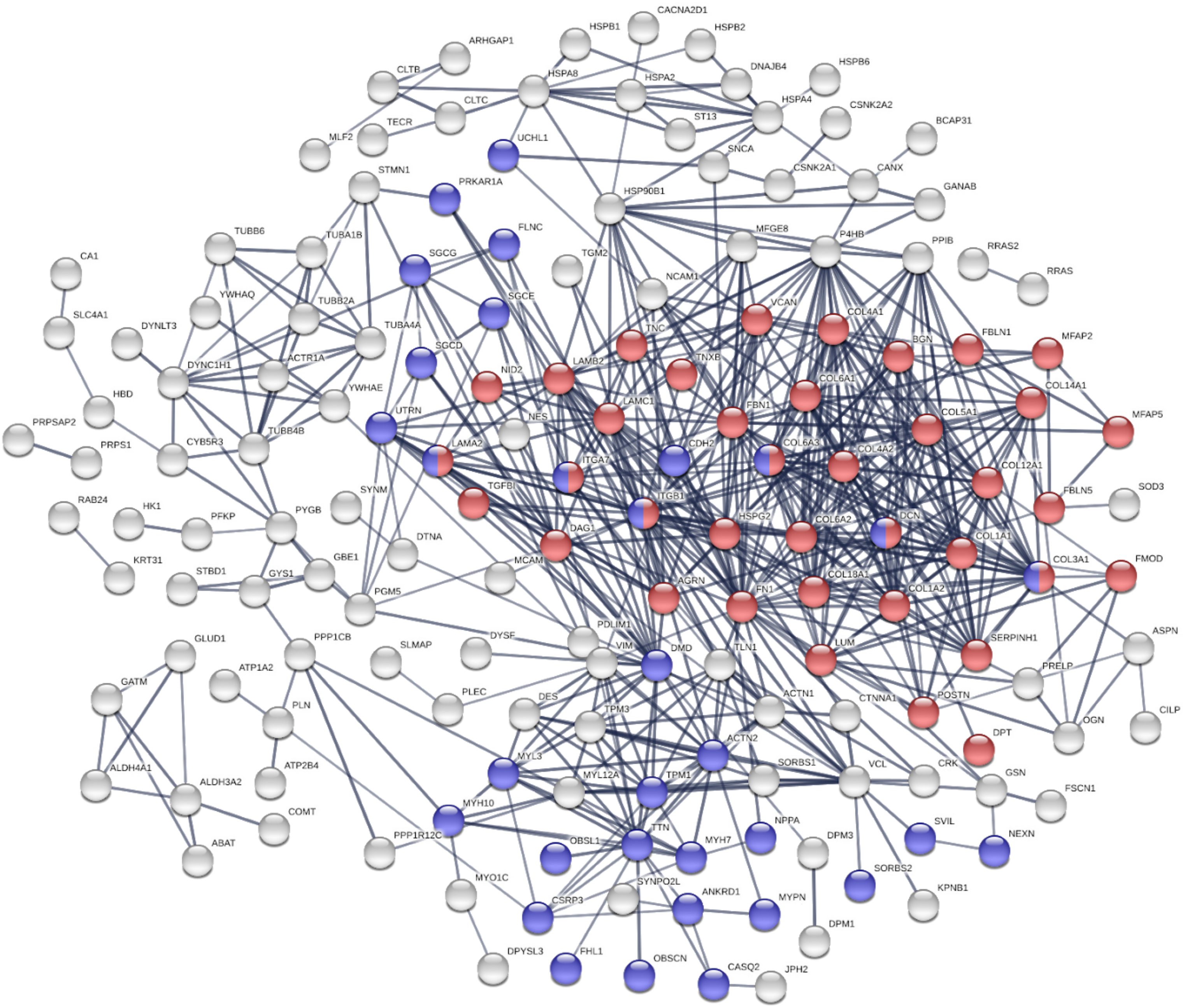

Protein-protein interactions of the up-regulated proteins in HCM versus control hearts using the proteomics data. Each node represents one protein, proteins involved in enriched extracellular matrix organization are shown in red nodes and proteins involved in enriched muscle structure development are shown in the blue nodes.

Supplementary Figure 1B

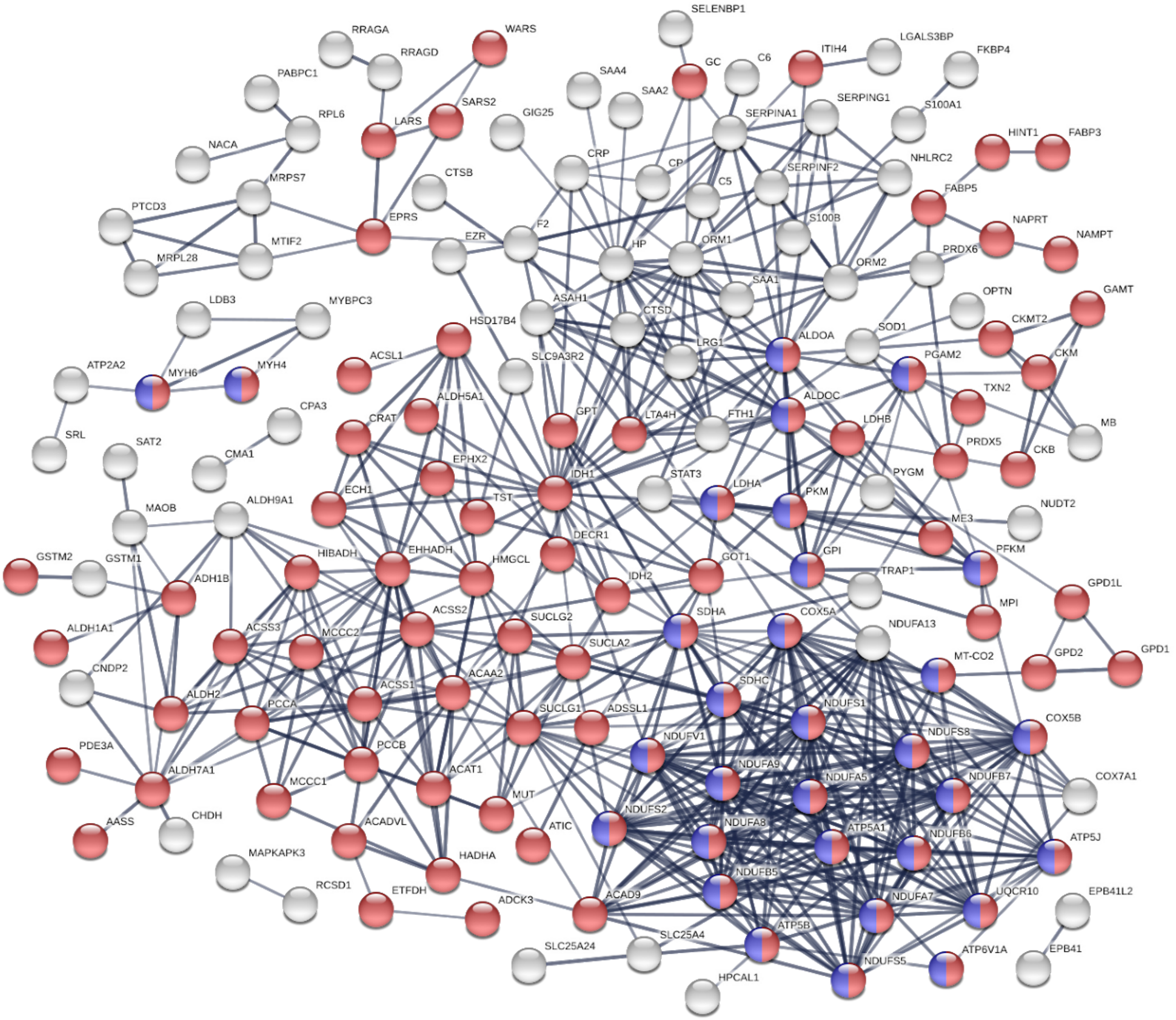

Protein-protein interactions of the down-regulated proteins in HCM versus control hearts using the proteomics data. Each node represents one protein, proteins involved in enriched small molecular metabolism are shown in red nodes and proteins involved in enriched ATP metabolic process are shown in the blue nodes.
