# Supplementary material for: Multi-omics integration identifies key upstream regulators of pathomechanisms in hypertrophic cardiomyopathy due to truncating MYBPC3 mutations
Source: Clin Epigenetics. 2021 Mar 23;13:61. doi: 10.1186/s13148-021-01043-3 (PMC7989210; doi:10.1186/s13148-021-01043-3)
Supplement: Supplementary file 3 — Additional file 3: Figure S3 (A) Gene set enrichment report showing the correlation between extracellular matrix (EC)-related genes and genes annotated to differentially acetylated regions. (B) Gene set enrichment report showing the correlation between ECM-related genes and differentially expressed genes. (C) Gene set enrichment report showing the correlation between ECM-related genes and genes encoding differentially expressed proteins. (D) Gene set enrichment report showing the correlation between cardiac muscle contraction-related genes and genes annotated to differentially acetylated regions. (E) Gene set enrichment report showing the correlation between cardiac muscle contraction-related genes and differentially expressed genes. (F) Gene set enrichment report showing the correlation between cardiac muscle contraction-related genes and genes encoding differentially expressed proteins. (G) Gene set enrichment report showing the correlation between fatty acid metabolism-related genes and genes annotated to differentially acetylated regions. (H) Gene set enrichment report showing the correlation between fatty acid metabolism-related genes and differentially expressed genes. (I) Gene set enrichment report showing the correlation between fatty acid metabolism-related genes and genes encoding differentially expressed proteins [file 13148_2021_1043_MOESM3_ESM.pdf]

GSEA Report for Dataset ChIPseq_genes_50Kb

Enrichment in phenotype: up (1 samples)

- 1 / 1 gene sets are upregulated in phenotype **up**
- 1 gene sets are significant at FDR < 25%
- 1 gene sets are significantly enriched at nominal pvalue < 1%
- 1 gene sets are significantly enriched at nominal pvalue < 5%
- Snapshot of enrichment results
- Detailed enrichment results in html format
- Detailed enrichment results in excel format (tab delimited text)
- Guide to interpret results

Enrichment in phenotype: down (1 samples)

- None of the gene sets are enriched in phenotype **down**
- Guide to interpret results

Dataset details

- The dataset has 11614 features (genes)
- No probe set => gene symbol collapsing was requested, so all 11614 features were used

Gene set details

- Gene set size filters (min=1, max=500) resulted in filtering out 0 / 1 gene sets
- The remaining 1 gene sets were used in the analysis
- List of gene sets used and their sizes (restricted to features in the specified dataset)

Gene markers for the up *versus* down comparison

- The dataset has 11614 features (genes)
- # of markers for phenotype **up**: 2795 (24.1%) with correlation area 23.9%
- # of markers for phenotype **down**: 8819 (75.9%) with correlation area 76.1%
- Detailed rank ordered gene list for all features in the dataset
- Heat map and gene list correlation profile for all features in the dataset

Global statistics and plots

- Plot of p-values *vs.* NES
- Global ES histogram

Other

- Parameters used for this analysis

Comments

- There were duplicate row identifiers in the specified dataset. One id was arbitarilly choosen. Details are below Generally, this is OK, but if you want to avoid this automagic, edit your dataset so that all row ids are unique # of

row ids in original dataset: 12672 # of row UNIQUE ids in original dataset: 11614 # The duplicates were TBC1D16 CCDC40 MXRA7 JMJD6 METTL23 SRSF2 MIR636 MFSD11 PACSIN2 TMEM230 PCNA PCNA-AS1 CDS2 NA ARHGAP22 WDFY4 ARAP1 MIR4692 CROCC MICALL2 INTS1 TNNC1 NISCH STAB1 CUEDC1 PODN GRHPR SYMPK FOXA3 RNF183 WDR31 BSPRY DDX56 TMED4 OGDH CH25H LIPA ASB13 SMC3 ZDHHC24 ACTN3 CTSF CCDC87 CCS APEH MST1 RNF123 AMIGO3 GMPPB RNF208 CYSRT1 RNF224 SLC34A3 FBXL12 UBL5 PIN1 TNFRSF19 FAM220A FAM186B PRPF40B MALAT1 SCYL1 EPM2AIP1 MLH1 ACOT11 TMF1 MIR3136 CKB TRMT61A BAG5 APOPT1 FLNB NRP1 GATD1 LOC171391 CEND1 SLC25A22 PANO1 PIDD1 RPLP2 SNORA52 PNPLA2 CRACR2B CD151 NFKBIA COX4I2 ABR GCH1 MIR4308 TFEB PGC GUCD1 SNRPD3 TRAK1 ZNF3 COPS6 MCM7 MIR25 MIR93 MIR106B AP4M1 TAF6 CNPY4 MBLAC1 LAMTOR4 TUBB4B FAM166A STPG3-AS1 STPG3 NELFB NAPA ZNF541 VPS37C VSIG10 FRMD1 SHB RPIA DYRK1A SIT1 RMRP CCDC107 ARHGEF39 CA9 TPM2 MIR6852 CYB5R3 ATP5MGL TCF7 HKR1 NPHP4 KCNAB2 MIR4251 TARBP1 DNAH12 PDE12 LOC101929679 MIR1276 TOR4A LOC100289511 SRSF5 DLG5 DLG5-AS1 ILF3-DT ILF3 KCNA5 TMEM189-UBE2V1 TMEM189 LINC01273 CEBPB-AS1 CEBPB EEFSEC NXNL1 SLC27A1 PGLS FAM129C LIPC-AS1 PTGIS ARL10 MIR1271 NOP16 HIGD2A CLTB FAF2 SNAPIN ILF2 INTS3 VPS53 FAM57A GEMIN4 DBIL5P GLOD4 MRM3 RNU6-2 PPP2CA MIR3661 CRAT PTPA ACP2 NR1H3 MADD LOC101928943 ADAMTSL4 MIR4257 LOC100131496 PSMD5 CUTALP PHF19 AP5Z1 MIR4656 LINC02525 SSBP3 HDAC9 MIR181C MIR181D NANOS3 PODNL1 DCAF15 SPDEF NFIA KIAA1217 SNUPN IMP3 SNX33 LINC02453 TMPO-AS1 TMPO GATD3A LOC100507346 PPFIBP1 FANCE RPL10A MIR7111 ULK1 PUS1 ARMC5 TGFB1I1 MED19 TMX2 TMX2-CTNND1 QRFP TPM1 GALE HMGCL ATP5MF-PTCD1 ATP5MF ZNF789 ZNF394 ZKSCAN5 C2orf81 WDR54 KRBA2 RPL26 RNF222 PBX1 ATP5MG NFIX DCST2 DCST1 DCST1-AS1 ADAM15 CEP85 SH3BGRL3 UBXN11 ZNF586 TGFBR2 DCTN1 DCTN1-AS1 MIR6881 RGMA CD80 SLC4A5 DHRS3 MIR6730 MIR4660 LINC01011 ABL1 CYGB PRCD SNHG16 SNORD1C SNORD1B SNORD1A ST6GALNAC2 INTS11 MIR6727 CPTP TAS1R3 LAD1 TNNI1 ABLIM2 SH3TC1 EIF3C EIF3CL MIR6862-1 MIR6862-2 SNORD89 PPP1R12B ASB3 ERLEC1 UBE4A SELENON MTFR1L LOC646471 IL18BP LOC100128494 MAP3K14-AS1 SPATA32 ZBTB21 ZNF295-AS1 TGFB1 PUS10 PEX13 KIAA1841 SLC5A6 ATRAID CAD CNBP NATD1 MAP2K3 EIF2S2 CSDC2 PMM1 DAPK2 CIAO2A SNX1 MIR7107 SELENOP CTBS LINC01461 LINC01555 DIPK1C CNDP2 SOST CDKN3 FAM20A ZCWPW1 MEPCE PPP1R35 C7orf61 TSC22D4 NYAP1 EML2 MIR330 EML2-AS1 POLDIP3 RNU12 ITGB1 SYNE2 MIR8077 CNFN LOC101930071 LIPE-AS1 NOC2L KLHL17 PLEKHN1 PERM1 CD14 TMCO6 P4HA2 MIR6830 PDLIM4 TMEM128 LYAR ZBTB49 SLC25A51 ZMIZ2 MIR3689A MIR3689C MIR3689D1 MIR3689B MIR3689D2 MIR3689E MIR3689F RPAP1 TYRO3 SLC48A1 HDAC7 KIAA0319L NCDN TFAP2E DNAL4 PLEKHB1 RNASEH1 RNASEH1-AS1 RPS7 NDEL1 GNMT PEX6 PPP2R5D MEA1 KLHDC3 RRP36 PICK1 MYOG DUSP3 CFAP97D1 AURKAIP1 CCNL2 MRPL20-AS1 MRPL20 CD7 SECTM1 TEX19 UTS2R EIF4ENIF1 SFI1 PPP1CC DVL1 MXRA8 MTHFSD FLJ30679 FOXC2-AS1 FOXC2 FOXL1 ADGRD1 ADGRD1-AS1 RPLP0 PXN-AS1 PXN SPON2 LOC100130872 CTBP1-AS WWP2 CFAP20 SAPCD1 SAPCD1-AS1 VWA7 CHD4 SCARNA11 LPAR5 ACRBP MIR4487 SLC39A13 THG1L HNRNPA3 MIR4444-1 MIR4444-2 TIMM44 NOL4L LOC101929698 DOK7 LRPAP1 PLLP CENPP LOC101929719 CYSTM1 TNNT2 PLA2G6 MAFF TMEM184B SLC35F6 CENPA ADD3-AS1 ADD3 GMFB CGRRF1 MIR8079 TUBA1C CBX5 MIR3198-2 HNRNPA1 HNRNPA1P10 NFE2 GPR162 P3H3 GNB3 CDCA3 USP5 TPI1 SPSB2 RPL13P5 DSTNP2 C16orf46 GCSH DNAJB4 GIPC2 PPM1L EIF2B2 MIR4269 MIR2467 C19orf25 PCSK4 REEP6 ADAMTSL5 PLK5 ARID5B MIR6762 SLC8B1 PLBD2 PLEKHA4 PPP1R15A MICAL3 MIR648 LINC01634 LINC01711 STX16 STX16-NPEPL1 TMEM222 ACTG1P20 SYTL1 RNF103-CHMP3 RMND5A CNOT11 LINC01968 TBC1D10A SF3A1 CCDC157 ABTB1 LINC01104 EXOSC10 MTOR-AS1 FGFBP3 BTAF1 LINC02593 SAMD11 GMCL1 SHROOM1 GDF9 UQCRQ LEAP2 GNGT2 PHOSPHO1 FLJ40194 MIR6129 CCSER2 MBOAT4 TUBB6 TPST2 MIR548J KIAA1191 SLC8A2 KPTN NAPA-AS1 TNXB ATF6B FKBPL ACSS1 RERE SF3B6 FAM228B TP53I3 PFN4 OSBP2 ESS2 TSSK2 GSC2 LINC01311 TUBA8 MIR6837 PGAM2 ICK FBXO9 PSMA5 AMIGO1 CEP55 TSPY26P PLAGL2 POFUT1 MIR1825 CYP17A1 XPC LSM3 EHD4-AS1 SLC2A11 MIF CYREN TMEM140 PMVK PBXIP1 PYGO2 SHC1 CKS1B MIR4258 FLAD1 LENEP ZBTB7B PIK3R2 IFI30 MPV17L2 RAB3A TBRG4 RAMP3 CDC14B KLHL3 TIMP3 BTBD16 SLC2A1 SLC2A1-AS1 PTPRM MIR3179-4 MIR3179-2 MIR3179-3 MIR3179-1 ASAP1 IL11RA CCL27 CCL19 MDC1 TUBB FLOT1 IER3 AFAP1 LOC389199 ZNF70 VPREB3 C22orf15 CHCHD10 MMP11 SMARCB1 APOC3 APOA1 APOA1-AS TTC39C SH2D3C TALDO1 ANKFY1 LOC103021295 PRPF19 TMEM109 CD164L2 GPR3 PPFIA4 CXCR5 BCL9L MIR4492 UPK2 ZNRD1ASP ZNRD1 PPP1R11 RNF39 TRIM31 TRIM31-AS1 ADAM11 PDE8A KCNQ1OT1 SMCR2 RAI1 TRIR ASNA1 BEST2 HOOK2 SMAD7 SERTAD4-AS1 SERTAD4 CELF2 PRRC2B CD81-AS1 CD81 TSSC4 TRPM5 R3HCC1 LOC100507156 PRRX2 FBXL18 LOC221946 ACTB MIR3909 MIR6069 HMOX1 NSMCE4A TACC2 MIR3137 XXYLT1-AS2 SLC30A3 DNAJC5G TRIM54 MAP3K14 PTPN11 CHD9 NDRG4 STARD10 ATG16L2 SRPRA FOXRED1 TIRAP DCPS LINC01132 MIR3183 ZNF774 IQGAP1 COL16A1 ZCCHC3 NRSN2-AS1 SOX12 SRF KLHL26 CRTC1 ACTN4 TNRC6C-AS1 TMC6 TMC8 C17orf99 SLC25A1 TGM7 RFC5 WSB2 ATE1 ATE1-AS1 MIR3177 G0S2 RNF157 UBALD2 CYP8B1 ZNF662 CENPT THAP11 NUTF2 MIR198 CALR3 C19orf44 KCNC3 NAPSB NAPSA NR1H2 UBAP2 MIR6888 TSC22D2 MAN1C1 RASA2 CLIC5 WDCP FKBP1B FLNC SPHK1 HERPUD1 BCAT2 HSD17B14 HEG1 MIR3960 MIR2861 CDK9 FPGS CLIP1-AS1 GALNT10 SH3RF1 CRYZL2P FCN1 NPR2 CIZ1 DNM1 MEX3D RBM17 TMEM11 PDCD2L TRIO C22orf23 POLR2F EFNA1 ANXA4 PLEKHA8P1 RNY5 ANO6 LOC283214 DGAT2 CELF1 PTPMT1 TRPV2 LRRC75A-AS1 SNORD49B SNORD49A SNORD65 LRRC75A TACC1 KLF13 VPS29 RAD9B WISP2 KCNK15 POLR3H ZP1 LOC105274304 NRARP PSPH CCT6A SNORA15 SUMF2 TUBB2B PSMG4 OGFOD3 HEXD MIR6764 SLC25A29 MIR345 SLC25A47 MRPS14 MIR3679 MEIS2 ANAPC1 MERTK SERPINB1 MIR4645 LIMA1 ATP2C1 MIR7161 MVB12A TMEM221 ACYP2 COL7A1 UQCRC1 TMEM89 SLC26A6 MIR6824 CELSR3 MIR4793 LINC02585 MIR1226 CWC25 MIR4727 C17orf98 KIRREL1 GRAMD2A PKM CRLF1 TMEM59L MRPL23-AS1 LINC01219 HOTS MIR675 HDDC2 TTYH2 DNAI2 HIST3H2A

HIST3H2BB MIR4666A RNF187 MFSD9 TMEM182 SNORD62B SNORD62A POMT1 ARHGEF19 CPLANE2 MARK3 CTBP1 CTBP1-DT SPIRE1 PSMG2 GGT5 POM121L9P SPECC1L SPECC1L-ADORA2A PFKFB3 MIR3155A MIR3155B RAI1-AS1 SMCR5 SREBF1 MIR6777 MIR33B ARL5C CACNB1 RPL19 FAM87B LINC00115 LINC01128 DYNC1LI2 SLC9A8 LINC00235 CAPN15 KCNE1 VDAC2 COMTD1 ITGAD COX6A2 ZNF843 LINC00211 DPF1 PPP1R14A C19orf33 EZR EZR-AS1 OSTCP1 MSMB NCOA4 MIR544B MIR1302-4 MIR5003 SH2D7 KIAA1324 SYDE2 CCT6B ZNF830 LIG3 SENP6 LOC101926963 MKRN2OS MKRN2 MIR598 BICRA LINC00327 LATS2 GATM SPATA5L1 IER5L SMG6 PSMA1 PARVB HTRA4 TJAP1 LRRC73 YIPF3 POLR1C MIR1234 EMP2 SELENOH BTBD18 CTNND1 IL22RA1 CDCA5 ZFPL1 TMEM262 VPS51 TM7SF2 ZNHIT2 FAU MRPL49 SYVN1 MIR6751 HERC3 ZNF496 FAM200A STK32C LRRC27 KLF11 DYSF E2F6 RBMS1 CPNE9 REEP1 LOC100996693 GMPPA MIR3132 OBSL1 INHA SH3BP2 ADD1 POLM MIR6838 KCNE2 SMIM11A RBP7 UBE4B ZSWIM4 FOSB CCNY SH3BP5-AS1 AEBP1 MIR4649 POLD2 MYL7 GCK TRIM63 PDIK1L MIR151B MIR342 PGF TUSC8 XXYLT1 SP2-AS1 PNPO PRR15L CDK5RAP3 PSMD8 GGN SPRED3 FAM98C RASGRP4 RYR1 IQCD TPCN1 EFHD1 MYLK NEK9 TMED10 PLEKHG5 PLCL2 MIR3714 IFT46 ARCN1 DYNLRB1 MAP1LC3A GLB1L STK16 TUBA4A MIR3654 EPC2 MRPL33 GTSE1-DT GTSE1 LDB1 PPRC1 NOLC1 MIR6808 CCL17 CIAPIN1 COQ9 WDR38 RPL35 ARPC5L FAM83D DHX35 CCIN CLTA MIR152 IFRD1 RWDD4 TRAPPC11 ZNF678 LMF1 LMF1-AS1 SOX8 DDAH2 CLIC1 MSH5 MSH5-SAPCD1 PDP2 TADA1 LTBP1 MIR6820 RDH13 EPS8L1 NAA20 TMEM231 GABARAPL2 MFN2 MIIP MIR6729 TNFRSF8 LMOD1 TIMM17A RNPEP UIMC1 ZNF346 DCAKD NMT1 FSTL1 MYO18A HTRA3 ADAMTSL4-AS1 MCL1 LOC101927040 RHCE MACO1 TGFBR3 SMU1 SPECC1 COL8A2 POGLUT1 TIMMDC1 MRPL42P5 HSF2BP MIR6070 RRP1B CBX2 CBX8 BCL2 KDSR SHISAL1 SLC30A5 ALDOB CCDC112 PSD3 NAALADL1 QRICH2 KCNK3 DNM1P46 ARHGEF7 ARHGEF7-AS1 LOC101060553 CYS1 GYG1 USP36 JAK1 LINC01359 UROS MIR4484 BCCIP SLC45A4

- Timestamp used as random seed: 1593880466903

---

xtools.gsea.Gsea [Sat, Jul 4, '20 6 PM 34] Report: ECM_ChIP_2.Gsea.1593880466851.rpt by user: Jiayi
Website: www.gsea-msigdb.org/gsea Questions & Suggestions: Contact page

GSEA Report for Dataset RNAseq_genes

Enrichment in phenotype: up (1 samples)

- 1 / 1 gene sets are upregulated in phenotype **up**
- 0 gene sets are significant at FDR < 25%
- 0 gene sets are significantly enriched at nominal pvalue < 1%
- 0 gene sets are significantly enriched at nominal pvalue < 5%
- Snapshot of enrichment results
- Detailed enrichment results in html format
- Detailed enrichment results in excel format (tab delimited text)
- Guide to interpret results

Enrichment in phenotype: down (1 samples)

- None of the gene sets are enriched in phenotype **down**
- Guide to interpret results

Dataset details

- The dataset has 2033 features (genes)
- No probe set => gene symbol collapsing was requested, so all 2033 features were used

Gene set details

- Gene set size filters (min=1, max=500) resulted in filtering out 0 / 1 gene sets
- The remaining 1 gene sets were used in the analysis
- List of gene sets used and their sizes (restricted to features in the specified dataset)

Gene markers for the up *versus* down comparison

- The dataset has 2033 features (genes)
- # of markers for phenotype **up**: 936 (46.0%) with correlation area 47.0%
- # of markers for phenotype **down**: 1097 (54.0%) with correlation area 53.0%
- Detailed rank ordered gene list for all features in the dataset
- Heat map and gene list correlation profile for all features in the dataset

Global statistics and plots

- Plot of p-values *vs.* NES
- Global ES histogram

Other

- Parameters used for this analysis

Comments

- There were duplicate row identifiers in the specified dataset. One id was arbitarilly choosen. Details are below Generally, this is OK, but if you want to avoid this automagic, edit your dataset so that all row ids are unique # of

row ids in original dataset: 2035 # of row UNIQUE ids in original dataset: 2033 # The duplicates were ZC3H11A TSTD3

- Timestamp used as random seed: 1593880443314

---

xtools.gsea.Gsea [Sat, Jul 4, '20 6 PM 34] Report: ECM_RNA_2.Gsea.1593880443298.rpt by user: Jiayi
Website: www.gsea-msigdb.org/gsea Questions & Suggestions: Contact page

GSEA Report for Dataset Proteome

Enrichment in phenotype: up (1 samples)

- 1 / 1 gene sets are upregulated in phenotype **up**
- 1 gene sets are significant at FDR < 25%
- 1 gene sets are significantly enriched at nominal pvalue < 1%
- 1 gene sets are significantly enriched at nominal pvalue < 5%
- Snapshot of enrichment results
- Detailed enrichment results in html format
- Detailed enrichment results in excel format (tab delimited text)
- Guide to interpret results

Enrichment in phenotype: down (1 samples)

- None of the gene sets are enriched in phenotype **down**
- Guide to interpret results

Dataset details

- The dataset has 432 features (genes)
- No probe set => gene symbol collapsing was requested, so all 432 features were used

Gene set details

- Gene set size filters (min=1, max=500) resulted in filtering out 0 / 1 gene sets
- The remaining 1 gene sets were used in the analysis
- List of gene sets used and their sizes (restricted to features in the specified dataset)

Gene markers for the up *versus* down comparison

- The dataset has 432 features (genes)
- # of markers for phenotype **up**: 209 (48.4%) with correlation area 50.7%
- # of markers for phenotype **down**: 223 (51.6%) with correlation area 49.3%
- Detailed rank ordered gene list for all features in the dataset
- Heat map and gene list correlation profile for all features in the dataset

Global statistics and plots

- Plot of p-values *vs.* NES
- Global ES histogram

Other

- Parameters used for this analysis

Comments

- There were duplicate row identifiers in the specified dataset. One id was arbitarilly choosen. Details are below Generally, this is OK, but if you want to avoid this automagic, edit your dataset so that all row ids are unique # of

row ids in original dataset: 441 # of row UNIQUE ids in original dataset: 432 # The duplicates were POSTN POSTN MAP4 POSTN VCAN PDLIM5 FBLN1 PKM PALLD

- Timestamp used as random seed: 1593880409450

---

xtools.gsea.Gsea [Sat, Jul 4, '20 6 PM 33]	Report: ECM_protein_2.Gsea.1593880409428.rpt by user: Jiayi<br>Website: www.gsea-msigdb.org/gsea Questions & Suggestions: Contact page

GSEA Report for Dataset ChIPseq_genes_50Kb

Enrichment in phenotype: up (1 samples)

- 1 / 1 gene sets are upregulated in phenotype **up**
- 1 gene sets are significant at FDR < 25%
- 0 gene sets are significantly enriched at nominal pvalue < 1%
- 0 gene sets are significantly enriched at nominal pvalue < 5%
- Snapshot of enrichment results
- Detailed enrichment results in html format
- Detailed enrichment results in excel format (tab delimited text)
- Guide to interpret results

Enrichment in phenotype: down (1 samples)

- None of the gene sets are enriched in phenotype **down**
- Guide to interpret results

Dataset details

- The dataset has 11614 features (genes)
- No probe set => gene symbol collapsing was requested, so all 11614 features were used

Gene set details

- Gene set size filters (min=1, max=500) resulted in filtering out 0 / 1 gene sets
- The remaining 1 gene sets were used in the analysis
- List of gene sets used and their sizes (restricted to features in the specified dataset)

Gene markers for the up *versus* down comparison

- The dataset has 11614 features (genes)
- # of markers for phenotype **up**: 2795 (24.1%) with correlation area 23.9%
- # of markers for phenotype **down**: 8819 (75.9%) with correlation area 76.1%
- Detailed rank ordered gene list for all features in the dataset
- Heat map and gene list correlation profile for all features in the dataset

Global statistics and plots

- Plot of p-values *vs.* NES
- Global ES histogram

Other

- Parameters used for this analysis

Comments

- There were duplicate row identifiers in the specified dataset. One id was arbitarilly choosen. Details are below Generally, this is OK, but if you want to avoid this automagic, edit your dataset so that all row ids are unique # of

row ids in original dataset: 12672 # of row UNIQUE ids in original dataset: 11614 # The duplicates were TBC1D16 CCDC40 MXRA7 JMJD6 METTL23 SRSF2 MIR636 MFSD11 PACSIN2 TMEM230 PCNA PCNA-AS1 CDS2 NA ARHGAP22 WDFY4 ARAP1 MIR4692 CROCC MICALL2 INTS1 TNNC1 NISCH STAB1 CUEDC1 PODN GRHPR SYMPK FOXA3 RNF183 WDR31 BSPRY DDX56 TMED4 OGDH CH25H LIPA ASB13 SMC3 ZDHHC24 ACTN3 CTSF CCDC87 CCS APEH MST1 RNF123 AMIGO3 GMPPB RNF208 CYSRT1 RNF224 SLC34A3 FBXL12 UBL5 PIN1 TNFRSF19 FAM220A FAM186B PRPF40B MALAT1 SCYL1 EPM2AIP1 MLH1 ACOT11 TMF1 MIR3136 CKB TRMT61A BAG5 APOPT1 FLNB NRP1 GATD1 LOC171391 CEND1 SLC25A22 PANO1 PIDD1 RPLP2 SNORA52 PNPLA2 CRACR2B CD151 NFKBIA COX4I2 ABR GCH1 MIR4308 TFEB PGC GUCD1 SNRPD3 TRAK1 ZNF3 COPS6 MCM7 MIR25 MIR93 MIR106B AP4M1 TAF6 CNPY4 MBLAC1 LAMTOR4 TUBB4B FAM166A STPG3-AS1 STPG3 NELFB NAPA ZNF541 VPS37C VSIG10 FRMD1 SHB RPIA DYRK1A SIT1 RMRP CCDC107 ARHGEF39 CA9 TPM2 MIR6852 CYB5R3 ATP5MGL TCF7 HKR1 NPHP4 KCNAB2 MIR4251 TARBP1 DNAH12 PDE12 LOC101929679 MIR1276 TOR4A LOC100289511 SRSF5 DLG5 DLG5-AS1 ILF3-DT ILF3 KCNA5 TMEM189-UBE2V1 TMEM189 LINC01273 CEBPB-AS1 CEBPB EEFSEC NXNL1 SLC27A1 PGLS FAM129C LIPC-AS1 PTGIS ARL10 MIR1271 NOP16 HIGD2A CLTB FAF2 SNAPIN ILF2 INTS3 VPS53 FAM57A GEMIN4 DBIL5P GLOD4 MRM3 RNU6-2 PPP2CA MIR3661 CRAT PTPA ACP2 NR1H3 MADD LOC101928943 ADAMTSL4 MIR4257 LOC100131496 PSMD5 CUTALP PHF19 AP5Z1 MIR4656 LINC02525 SSBP3 HDAC9 MIR181C MIR181D NANOS3 PODNL1 DCAF15 SPDEF NFIA KIAA1217 SNUPN IMP3 SNX33 LINC02453 TMPO-AS1 TMPO GATD3A LOC100507346 PPFIBP1 FANCE RPL10A MIR7111 ULK1 PUS1 ARMC5 TGFB1I1 MED19 TMX2 TMX2-CTNND1 QRFP TPM1 GALE HMGCL ATP5MF-PTCD1 ATP5MF ZNF789 ZNF394 ZKSCAN5 C2orf81 WDR54 KRBA2 RPL26 RNF222 PBX1 ATP5MG NFIX DCST2 DCST1 DCST1-AS1 ADAM15 CEP85 SH3BGRL3 UBXN11 ZNF586 TGFBR2 DCTN1 DCTN1-AS1 MIR6881 RGMA CD80 SLC4A5 DHRS3 MIR6730 MIR4660 LINC01011 ABL1 CYGB PRCD SNHG16 SNORD1C SNORD1B SNORD1A ST6GALNAC2 INTS11 MIR6727 CPTP TAS1R3 LAD1 TNNI1 ABLIM2 SH3TC1 EIF3C EIF3CL MIR6862-1 MIR6862-2 SNORD89 PPP1R12B ASB3 ERLEC1 UBE4A SELENON MTFR1L LOC646471 IL18BP LOC100128494 MAP3K14-AS1 SPATA32 ZBTB21 ZNF295-AS1 TGFB1 PUS10 PEX13 KIAA1841 SLC5A6 ATRAID CAD CNBP NATD1 MAP2K3 EIF2S2 CSDC2 PMM1 DAPK2 CIAO2A SNX1 MIR7107 SELENOP CTBS LINC01461 LINC01555 DIPK1C CNDP2 SOST CDKN3 FAM20A ZCWPW1 MEPCE PPP1R35 C7orf61 TSC22D4 NYAP1 EML2 MIR330 EML2-AS1 POLDIP3 RNU12 ITGB1 SYNE2 MIR8077 CNFN LOC101930071 LIPE-AS1 NOC2L KLHL17 PLEKHN1 PERM1 CD14 TMCO6 P4HA2 MIR6830 PDLIM4 TMEM128 LYAR ZBTB49 SLC25A51 ZMIZ2 MIR3689A MIR3689C MIR3689D1 MIR3689B MIR3689D2 MIR3689E MIR3689F RPAP1 TYRO3 SLC48A1 HDAC7 KIAA0319L NCDN TFAP2E DNAL4 PLEKHB1 RNASEH1 RNASEH1-AS1 RPS7 NDEL1 GNMT PEX6 PPP2R5D MEA1 KLHDC3 RRP36 PICK1 MYOG DUSP3 CFAP97D1 AURKAIP1 CCNL2 MRPL20-AS1 MRPL20 CD7 SECTM1 TEX19 UTS2R EIF4ENIF1 SFI1 PPP1CC DVL1 MXRA8 MTHFSD FLJ30679 FOXC2-AS1 FOXC2 FOXL1 ADGRD1 ADGRD1-AS1 RPLP0 PXN-AS1 PXN SPON2 LOC100130872 CTBP1-AS WWP2 CFAP20 SAPCD1 SAPCD1-AS1 VWA7 CHD4 SCARNA11 LPAR5 ACRBP MIR4487 SLC39A13 THG1L HNRNPA3 MIR4444-1 MIR4444-2 TIMM44 NOL4L LOC101929698 DOK7 LRPAP1 PLLP CENPP LOC101929719 CYSTM1 TNNT2 PLA2G6 MAFF TMEM184B SLC35F6 CENPA ADD3-AS1 ADD3 GMFB CGRRF1 MIR8079 TUBA1C CBX5 MIR3198-2 HNRNPA1 HNRNPA1P10 NFE2 GPR162 P3H3 GNB3 CDCA3 USP5 TPI1 SPSB2 RPL13P5 DSTNP2 C16orf46 GCSH DNAJB4 GIPC2 PPM1L EIF2B2 MIR4269 MIR2467 C19orf25 PCSK4 REEP6 ADAMTSL5 PLK5 ARID5B MIR6762 SLC8B1 PLBD2 PLEKHA4 PPP1R15A MICAL3 MIR648 LINC01634 LINC01711 STX16 STX16-NPEPL1 TMEM222 ACTG1P20 SYTL1 RNF103-CHMP3 RMND5A CNOT11 LINC01968 TBC1D10A SF3A1 CCDC157 ABTB1 LINC01104 EXOSC10 MTOR-AS1 FGFBP3 BTAF1 LINC02593 SAMD11 GMCL1 SHROOM1 GDF9 UQCRQ LEAP2 GNGT2 PHOSPHO1 FLJ40194 MIR6129 CCSER2 MBOAT4 TUBB6 TPST2 MIR548J KIAA1191 SLC8A2 KPTN NAPA-AS1 TNXB ATF6B FKBPL ACSS1 RERE SF3B6 FAM228B TP53I3 PFN4 OSBP2 ESS2 TSSK2 GSC2 LINC01311 TUBA8 MIR6837 PGAM2 ICK FBXO9 PSMA5 AMIGO1 CEP55 TSPY26P PLAGL2 POFUT1 MIR1825 CYP17A1 XPC LSM3 EHD4-AS1 SLC2A11 MIF CYREN TMEM140 PMVK PBXIP1 PYGO2 SHC1 CKS1B MIR4258 FLAD1 LENEP ZBTB7B PIK3R2 IFI30 MPV17L2 RAB3A TBRG4 RAMP3 CDC14B KLHL3 TIMP3 BTBD16 SLC2A1 SLC2A1-AS1 PTPRM MIR3179-4 MIR3179-2 MIR3179-3 MIR3179-1 ASAP1 IL11RA CCL27 CCL19 MDC1 TUBB FLOT1 IER3 AFAP1 LOC389199 ZNF70 VPREB3 C22orf15 CHCHD10 MMP11 SMARCB1 APOC3 APOA1 APOA1-AS TTC39C SH2D3C TALDO1 ANKFY1 LOC103021295 PRPF19 TMEM109 CD164L2 GPR3 PPFIA4 CXCR5 BCL9L MIR4492 UPK2 ZNRD1ASP ZNRD1 PPP1R11 RNF39 TRIM31 TRIM31-AS1 ADAM11 PDE8A KCNQ1OT1 SMCR2 RAI1 TRIR ASNA1 BEST2 HOOK2 SMAD7 SERTAD4-AS1 SERTAD4 CELF2 PRRC2B CD81-AS1 CD81 TSSC4 TRPM5 R3HCC1 LOC100507156 PRRX2 FBXL18 LOC221946 ACTB MIR3909 MIR6069 HMOX1 NSMCE4A TACC2 MIR3137 XXYLT1-AS2 SLC30A3 DNAJC5G TRIM54 MAP3K14 PTPN11 CHD9 NDRG4 STARD10 ATG16L2 SRPRA FOXRED1 TIRAP DCPS LINC01132 MIR3183 ZNF774 IQGAP1 COL16A1 ZCCHC3 NRSN2-AS1 SOX12 SRF KLHL26 CRTC1 ACTN4 TNRC6C-AS1 TMC6 TMC8 C17orf99 SLC25A1 TGM7 RFC5 WSB2 ATE1 ATE1-AS1 MIR3177 G0S2 RNF157 UBALD2 CYP8B1 ZNF662 CENPT THAP11 NUTF2 MIR198 CALR3 C19orf44 KCNC3 NAPSB NAPSA NR1H2 UBAP2 MIR6888 TSC22D2 MAN1C1 RASA2 CLIC5 WDCP FKBP1B FLNC SPHK1 HERPUD1 BCAT2 HSD17B14 HEG1 MIR3960 MIR2861 CDK9 FPGS CLIP1-AS1 GALNT10 SH3RF1 CRYZL2P FCN1 NPR2 CIZ1 DNM1 MEX3D RBM17 TMEM11 PDCD2L TRIO C22orf23 POLR2F EFNA1 ANXA4 PLEKHA8P1 RNY5 ANO6 LOC283214 DGAT2 CELF1 PTPMT1 TRPV2 LRRC75A-AS1 SNORD49B SNORD49A SNORD65 LRRC75A TACC1 KLF13 VPS29 RAD9B WISP2 KCNK15 POLR3H ZP1 LOC105274304 NRARP PSPH CCT6A SNORA15 SUMF2 TUBB2B PSMG4 OGFOD3 HEXD MIR6764 SLC25A29 MIR345 SLC25A47 MRPS14 MIR3679 MEIS2 ANAPC1 MERTK SERPINB1 MIR4645 LIMA1 ATP2C1 MIR7161 MVB12A TMEM221 ACYP2 COL7A1 UQCRC1 TMEM89 SLC26A6 MIR6824 CELSR3 MIR4793 LINC02585 MIR1226 CWC25 MIR4727 C17orf98 KIRREL1 GRAMD2A PKM CRLF1 TMEM59L MRPL23-AS1 LINC01219 HOTS MIR675 HDDC2 TTYH2 DNAI2 HIST3H2A

HIST3H2BB MIR4666A RNF187 MFSD9 TMEM182 SNORD62B SNORD62A POMT1 ARHGEF19 CPLANE2 MARK3 CTBP1 CTBP1-DT SPIRE1 PSMG2 GGT5 POM121L9P SPECC1L SPECC1L-ADORA2A PFKFB3 MIR3155A MIR3155B RAI1-AS1 SMCR5 SREBF1 MIR6777 MIR33B ARL5C CACNB1 RPL19 FAM87B LINC00115 LINC01128 DYNC1LI2 SLC9A8 LINC00235 CAPN15 KCNE1 VDAC2 COMTD1 ITGAD COX6A2 ZNF843 LINC00211 DPF1 PPP1R14A C19orf33 EZR EZR-AS1 OSTCP1 MSMB NCOA4 MIR544B MIR1302-4 MIR5003 SH2D7 KIAA1324 SYDE2 CCT6B ZNF830 LIG3 SENP6 LOC101926963 MKRN2OS MKRN2 MIR598 BICRA LINC00327 LATS2 GATM SPATA5L1 IER5L SMG6 PSMA1 PARVB HTRA4 TJAP1 LRRC73 YIPF3 POLR1C MIR1234 EMP2 SELENOH BTBD18 CTNND1 IL22RA1 CDCA5 ZFPL1 TMEM262 VPS51 TM7SF2 ZNHIT2 FAU MRPL49 SYVN1 MIR6751 HERC3 ZNF496 FAM200A STK32C LRRC27 KLF11 DYSF E2F6 RBMS1 CPNE9 REEP1 LOC100996693 GMPPA MIR3132 OBSL1 INHA SH3BP2 ADD1 POLM MIR6838 KCNE2 SMIM11A RBP7 UBE4B ZSWIM4 FOSB CCNY SH3BP5-AS1 AEBP1 MIR4649 POLD2 MYL7 GCK TRIM63 PDIK1L MIR151B MIR342 PGF TUSC8 XXYLT1 SP2-AS1 PNPO PRR15L CDK5RAP3 PSMD8 GGN SPRED3 FAM98C RASGRP4 RYR1 IQCD TPCN1 EFHD1 MYLK NEK9 TMED10 PLEKHG5 PLCL2 MIR3714 IFT46 ARCN1 DYNLRB1 MAP1LC3A GLB1L STK16 TUBA4A MIR3654 EPC2 MRPL33 GTSE1-DT GTSE1 LDB1 PPRC1 NOLC1 MIR6808 CCL17 CIAPIN1 COQ9 WDR38 RPL35 ARPC5L FAM83D DHX35 CCIN CLTA MIR152 IFRD1 RWDD4 TRAPPC11 ZNF678 LMF1 LMF1-AS1 SOX8 DDAH2 CLIC1 MSH5 MSH5-SAPCD1 PDP2 TADA1 LTBP1 MIR6820 RDH13 EPS8L1 NAA20 TMEM231 GABARAPL2 MFN2 MIIP MIR6729 TNFRSF8 LMOD1 TIMM17A RNPEP UIMC1 ZNF346 DCAKD NMT1 FSTL1 MYO18A HTRA3 ADAMTSL4-AS1 MCL1 LOC101927040 RHCE MACO1 TGFBR3 SMU1 SPECC1 COL8A2 POGLUT1 TIMMDC1 MRPL42P5 HSF2BP MIR6070 RRP1B CBX2 CBX8 BCL2 KDSR SHISAL1 SLC30A5 ALDOB CCDC112 PSD3 NAALADL1 QRICH2 KCNK3 DNM1P46 ARHGEF7 ARHGEF7-AS1 LOC101060553 CYS1 GYG1 USP36 JAK1 LINC01359 UROS MIR4484 BCCIP SLC45A4

- Timestamp used as random seed: 1593880105619

---

xtools.gsea.Gsea [Sat, Jul 4, '20 6 PM 28] Report: Muscle_ChIP_cardiac.Gsea.1593880105581.rpt by user: Jiayi
Website: www.gsea-msigdb.org/gsea Questions & Suggestions: Contact page

GSEA Report for Dataset RNAseq_genes

Enrichment in phenotype: up (1 samples)

- 1 / 1 gene sets are upregulated in phenotype **up**
- 1 gene sets are significant at FDR < 25%
- 0 gene sets are significantly enriched at nominal pvalue < 1%
- 0 gene sets are significantly enriched at nominal pvalue < 5%
- Snapshot of enrichment results
- Detailed enrichment results in html format
- Detailed enrichment results in excel format (tab delimited text)
- Guide to interpret results

Enrichment in phenotype: down (1 samples)

- None of the gene sets are enriched in phenotype **down**
- Guide to interpret results

Dataset details

- The dataset has 2033 features (genes)
- No probe set => gene symbol collapsing was requested, so all 2033 features were used

Gene set details

- Gene set size filters (min=1, max=500) resulted in filtering out 0 / 1 gene sets
- The remaining 1 gene sets were used in the analysis
- List of gene sets used and their sizes (restricted to features in the specified dataset)

Gene markers for the up *versus* down comparison

- The dataset has 2033 features (genes)
- # of markers for phenotype **up**: 936 (46.0%) with correlation area 47.0%
- # of markers for phenotype **down**: 1097 (54.0%) with correlation area 53.0%
- Detailed rank ordered gene list for all features in the dataset
- Heat map and gene list correlation profile for all features in the dataset

Global statistics and plots

- Plot of p-values *vs.* NES
- Global ES histogram

Other

- Parameters used for this analysis

Comments

- There were duplicate row identifiers in the specified dataset. One id was arbitarilly choosen. Details are below Generally, this is OK, but if you want to avoid this automagic, edit your dataset so that all row ids are unique # of

row ids in original dataset: 2035 # of row UNIQUE ids in original dataset: 2033 # The duplicates were ZC3H11A TSTD3

- Timestamp used as random seed: 1593880076262

---

xtools.gsea.Gsea [Sat, Jul 4, '20 6 PM 27] Report: Muscle_RNA_cardiac.Gsea.1593880076246.rpt by user: Jiayi
Website: www.gsea-msigdb.org/gsea Questions & Suggestions: Contact page

GSEA Report for Dataset Proteome

Enrichment in phenotype: up (1 samples)

- 1 / 1 gene sets are upregulated in phenotype **up**
- 0 gene sets are significant at FDR < 25%
- 0 gene sets are significantly enriched at nominal pvalue < 1%
- 0 gene sets are significantly enriched at nominal pvalue < 5%
- Snapshot of enrichment results
- Detailed enrichment results in html format
- Detailed enrichment results in excel format (tab delimited text)
- Guide to interpret results

Enrichment in phenotype: down (1 samples)

- None of the gene sets are enriched in phenotype **down**
- Guide to interpret results

Dataset details

- The dataset has 432 features (genes)
- No probe set => gene symbol collapsing was requested, so all 432 features were used

Gene set details

- Gene set size filters (min=1, max=500) resulted in filtering out 0 / 1 gene sets
- The remaining 1 gene sets were used in the analysis
- List of gene sets used and their sizes (restricted to features in the specified dataset)

Gene markers for the up *versus* down comparison

- The dataset has 432 features (genes)
- # of markers for phenotype **up**: 209 (48.4%) with correlation area 50.7%
- # of markers for phenotype **down**: 223 (51.6%) with correlation area 49.3%
- Detailed rank ordered gene list for all features in the dataset
- Heat map and gene list correlation profile for all features in the dataset

Global statistics and plots

- Plot of p-values *vs.* NES
- Global ES histogram

Other

- Parameters used for this analysis

Comments

- There were duplicate row identifiers in the specified dataset. One id was arbitarilly choosen. Details are below Generally, this is OK, but if you want to avoid this automagic, edit your dataset so that all row ids are unique # of

row ids in original dataset: 441 # of row UNIQUE ids in original dataset: 432 # The duplicates were POSTN POSTN MAP4 POSTN VCAN PDLIM5 FBLN1 PKM PALLD

- Timestamp used as random seed: 1593880042015

---

xtools.gsea.Gsea [Sat, Jul 4, '20 6 PM 27] Report: Muscle_protein_cardiac.Gsea.1593880041991.rpt by user: Jiayi
Website: www.gsea-msigdb.org/gsea Questions & Suggestions: Contact page

GSEA Report for Dataset ChIPseq_genes_50Kb

Enrichment in phenotype: up (1 samples)

- None of the gene sets are enriched in phenotype **up**
- Guide to interpret results

Enrichment in phenotype: down (1 samples)

- 1 / 1 gene sets are upregulated in phenotype **down**
- 1 gene sets are significantly enriched at FDR < 25%
- 0 gene sets are significantly enriched at nominal pvalue < 1%
- 0 gene sets are significantly enriched at nominal pvalue < 5%
- Snapshot of enrichment results
- Detailed enrichment results in html format
- Detailed enrichment results in excel format (tab delimited text)
- Guide to interpret results

Dataset details

- The dataset has 11614 features (genes)
- No probe set => gene symbol collapsing was requested, so all 11614 features were used

Gene set details

- Gene set size filters (min=1, max=500) resulted in filtering out 0 / 1 gene sets
- The remaining 1 gene sets were used in the analysis
- List of gene sets used and their sizes (restricted to features in the specified dataset)

Gene markers for the up *versus* down comparison

- The dataset has 11614 features (genes)
- # of markers for phenotype **up**: 2795 (24.1%) with correlation area 23.9%
- # of markers for phenotype **down**: 8819 (75.9%) with correlation area 76.1%
- Detailed rank ordered gene list for all features in the dataset
- Heat map and gene list correlation profile for all features in the dataset

Global statistics and plots

- Plot of p-values *vs.* NES
- Global ES histogram

Other

- Parameters used for this analysis

Comments

- There were duplicate row identifiers in the specified dataset. One id was arbitarilly choosen. Details are below Generally, this is OK, but if you want to avoid this automagic, edit your dataset so that all row ids are unique # of

row ids in original dataset: 12672 # of row UNIQUE ids in original dataset: 11614 # The duplicates were TBC1D16 CCDC40 MXRA7 JMJD6 METTL23 SRSF2 MIR636 MFSD11 PACSIN2 TMEM230 PCNA PCNA-AS1 CDS2 NA ARHGAP22 WDFY4 ARAP1 MIR4692 CROCC MICALL2 INTS1 TNNC1 NISCH STAB1 CUEDC1 PODN GRHPR SYMPK FOXA3 RNF183 WDR31 BSPRY DDX56 TMED4 OGDH CH25H LIPA ASB13 SMC3 ZDHHC24 ACTN3 CTSF CCDC87 CCS APEH MST1 RNF123 AMIGO3 GMPPB RNF208 CYSRT1 RNF224 SLC34A3 FBXL12 UBL5 PIN1 TNFRSF19 FAM220A FAM186B PRPF40B MALAT1 SCYL1 EPM2AIP1 MLH1 ACOT11 TMF1 MIR3136 CKB TRMT61A BAG5 APOPT1 FLNB NRP1 GATD1 LOC171391 CEND1 SLC25A22 PANO1 PIDD1 RPLP2 SNORA52 PNPLA2 CRACR2B CD151 NFKBIA COX4I2 ABR GCH1 MIR4308 TFEB PGC GUCD1 SNRPD3 TRAK1 ZNF3 COPS6 MCM7 MIR25 MIR93 MIR106B AP4M1 TAF6 CNPY4 MBLAC1 LAMTOR4 TUBB4B FAM166A STPG3-AS1 STPG3 NELFB NAPA ZNF541 VPS37C VSIG10 FRMD1 SHB RPIA DYRK1A SIT1 RMRP CCDC107 ARHGEF39 CA9 TPM2 MIR6852 CYB5R3 ATP5MGL TCF7 HKR1 NPHP4 KCNAB2 MIR4251 TARBP1 DNAH12 PDE12 LOC101929679 MIR1276 TOR4A LOC100289511 SRSF5 DLG5 DLG5-AS1 ILF3-DT ILF3 KCNA5 TMEM189-UBE2V1 TMEM189 LINC01273 CEBPB-AS1 CEBPB EEFSEC NXNL1 SLC27A1 PGLS FAM129C LIPC-AS1 PTGIS ARL10 MIR1271 NOP16 HIGD2A CLTB FAF2 SNAPIN ILF2 INTS3 VPS53 FAM57A GEMIN4 DBIL5P GLOD4 MRM3 RNU6-2 PPP2CA MIR3661 CRAT PTPA ACP2 NR1H3 MADD LOC101928943 ADAMTSL4 MIR4257 LOC100131496 PSMD5 CUTALP PHF19 AP5Z1 MIR4656 LINC02525 SSBP3 HDAC9 MIR181C MIR181D NANOS3 PODNL1 DCAF15 SPDEF NFIA KIAA1217 SNUPN IMP3 SNX33 LINC02453 TMPO-AS1 TMPO GATD3A LOC100507346 PPFIBP1 FANCE RPL10A MIR7111 ULK1 PUS1 ARMC5 TGFB1I1 MED19 TMX2 TMX2-CTNND1 QRFP TPM1 GALE HMGCL ATP5MF-PTCD1 ATP5MF ZNF789 ZNF394 ZKSCAN5 C2orf81 WDR54 KRBA2 RPL26 RNF222 PBX1 ATP5MG NFIX DCST2 DCST1 DCST1-AS1 ADAM15 CEP85 SH3BGRL3 UBXN11 ZNF586 TGFBR2 DCTN1 DCTN1-AS1 MIR6881 RGMA CD80 SLC4A5 DHRS3 MIR6730 MIR4660 LINC01011 ABL1 CYGB PRCD SNHG16 SNORD1C SNORD1B SNORD1A ST6GALNAC2 INTS11 MIR6727 CPTP TAS1R3 LAD1 TNNI1 ABLIM2 SH3TC1 EIF3C EIF3CL MIR6862-1 MIR6862-2 SNORD89 PPP1R12B ASB3 ERLEC1 UBE4A SELENON MTFR1L LOC646471 IL18BP LOC100128494 MAP3K14-AS1 SPATA32 ZBTB21 ZNF295-AS1 TGFB1 PUS10 PEX13 KIAA1841 SLC5A6 ATRAID CAD CNBP NATD1 MAP2K3 EIF2S2 CSDC2 PMM1 DAPK2 CIAO2A SNX1 MIR7107 SELENOP CTBS LINC01461 LINC01555 DIPK1C CNDP2 SOST CDKN3 FAM20A ZCWPW1 MEPCE PPP1R35 C7orf61 TSC22D4 NYAP1 EML2 MIR330 EML2-AS1 POLDIP3 RNU12 ITGB1 SYNE2 MIR8077 CNFN LOC101930071 LIPE-AS1 NOC2L KLHL17 PLEKHN1 PERM1 CD14 TMCO6 P4HA2 MIR6830 PDLIM4 TMEM128 LYAR ZBTB49 SLC25A51 ZMIZ2 MIR3689A MIR3689C MIR3689D1 MIR3689B MIR3689D2 MIR3689E MIR3689F RPAP1 TYRO3 SLC48A1 HDAC7 KIAA0319L NCDN TFAP2E DNAL4 PLEKHB1 RNASEH1 RNASEH1-AS1 RPS7 NDEL1 GNMT PEX6 PPP2R5D MEA1 KLHDC3 RRP36 PICK1 MYOG DUSP3 CFAP97D1 AURKAIP1 CCNL2 MRPL20-AS1 MRPL20 CD7 SECTM1 TEX19 UTS2R EIF4ENIF1 SFI1 PPP1CC DVL1 MXRA8 MTHFSD FLJ30679 FOXC2-AS1 FOXC2 FOXL1 ADGRD1 ADGRD1-AS1 RPLP0 PXN-AS1 PXN SPON2 LOC100130872 CTBP1-AS WWP2 CFAP20 SAPCD1 SAPCD1-AS1 VWA7 CHD4 SCARNA11 LPAR5 ACRBP MIR4487 SLC39A13 THG1L HNRNPA3 MIR4444-1 MIR4444-2 TIMM44 NOL4L LOC101929698 DOK7 LRPAP1 PLLP CENPP LOC101929719 CYSTM1 TNNT2 PLA2G6 MAFF TMEM184B SLC35F6 CENPA ADD3-AS1 ADD3 GMFB CGRRF1 MIR8079 TUBA1C CBX5 MIR3198-2 HNRNPA1 HNRNPA1P10 NFE2 GPR162 P3H3 GNB3 CDCA3 USP5 TPI1 SPSB2 RPL13P5 DSTNP2 C16orf46 GCSH DNAJB4 GIPC2 PPM1L EIF2B2 MIR4269 MIR2467 C19orf25 PCSK4 REEP6 ADAMTSL5 PLK5 ARID5B MIR6762 SLC8B1 PLBD2 PLEKHA4 PPP1R15A MICAL3 MIR648 LINC01634 LINC01711 STX16 STX16-NPEPL1 TMEM222 ACTG1P20 SYTL1 RNF103-CHMP3 RMND5A CNOT11 LINC01968 TBC1D10A SF3A1 CCDC157 ABTB1 LINC01104 EXOSC10 MTOR-AS1 FGFBP3 BTAF1 LINC02593 SAMD11 GMCL1 SHROOM1 GDF9 UQCRQ LEAP2 GNGT2 PHOSPHO1 FLJ40194 MIR6129 CCSER2 MBOAT4 TUBB6 TPST2 MIR548J KIAA1191 SLC8A2 KPTN NAPA-AS1 TNXB ATF6B FKBPL ACSS1 RERE SF3B6 FAM228B TP53I3 PFN4 OSBP2 ESS2 TSSK2 GSC2 LINC01311 TUBA8 MIR6837 PGAM2 ICK FBXO9 PSMA5 AMIGO1 CEP55 TSPY26P PLAGL2 POFUT1 MIR1825 CYP17A1 XPC LSM3 EHD4-AS1 SLC2A11 MIF CYREN TMEM140 PMVK PBXIP1 PYGO2 SHC1 CKS1B MIR4258 FLAD1 LENEP ZBTB7B PIK3R2 IFI30 MPV17L2 RAB3A TBRG4 RAMP3 CDC14B KLHL3 TIMP3 BTBD16 SLC2A1 SLC2A1-AS1 PTPRM MIR3179-4 MIR3179-2 MIR3179-3 MIR3179-1 ASAP1 IL11RA CCL27 CCL19 MDC1 TUBB FLOT1 IER3 AFAP1 LOC389199 ZNF70 VPREB3 C22orf15 CHCHD10 MMP11 SMARCB1 APOC3 APOA1 APOA1-AS TTC39C SH2D3C TALDO1 ANKFY1 LOC103021295 PRPF19 TMEM109 CD164L2 GPR3 PPFIA4 CXCR5 BCL9L MIR4492 UPK2 ZNRD1ASP ZNRD1 PPP1R11 RNF39 TRIM31 TRIM31-AS1 ADAM11 PDE8A KCNQ1OT1 SMCR2 RAI1 TRIR ASNA1 BEST2 HOOK2 SMAD7 SERTAD4-AS1 SERTAD4 CELF2 PRRC2B CD81-AS1 CD81 TSSC4 TRPM5 R3HCC1 LOC100507156 PRRX2 FBXL18 LOC221946 ACTB MIR3909 MIR6069 HMOX1 NSMCE4A TACC2 MIR3137 XXYLT1-AS2 SLC30A3 DNAJC5G TRIM54 MAP3K14 PTPN11 CHD9 NDRG4 STARD10 ATG16L2 SRPRA FOXRED1 TIRAP DCPS LINC01132 MIR3183 ZNF774 IQGAP1 COL16A1 ZCCHC3 NRSN2-AS1 SOX12 SRF KLHL26 CRTC1 ACTN4 TNRC6C-AS1 TMC6 TMC8 C17orf99 SLC25A1 TGM7 RFC5 WSB2 ATE1 ATE1-AS1 MIR3177 G0S2 RNF157 UBALD2 CYP8B1 ZNF662 CENPT THAP11 NUTF2 MIR198 CALR3 C19orf44 KCNC3 NAPSB NAPSA NR1H2 UBAP2 MIR6888 TSC22D2 MAN1C1 RASA2 CLIC5 WDCP FKBP1B FLNC SPHK1 HERPUD1 BCAT2 HSD17B14 HEG1 MIR3960 MIR2861 CDK9 FPGS CLIP1-AS1 GALNT10 SH3RF1 CRYZL2P FCN1 NPR2 CIZ1 DNM1 MEX3D RBM17 TMEM11 PDCD2L TRIO C22orf23 POLR2F EFNA1 ANXA4 PLEKHA8P1 RNY5 ANO6 LOC283214 DGAT2 CELF1 PTPMT1 TRPV2 LRRC75A-AS1 SNORD49B SNORD49A SNORD65 LRRC75A TACC1 KLF13 VPS29 RAD9B WISP2 KCNK15 POLR3H ZP1 LOC105274304 NRARP PSPH CCT6A SNORA15 SUMF2 TUBB2B PSMG4 OGFOD3 HEXD MIR6764 SLC25A29 MIR345 SLC25A47 MRPS14 MIR3679 MEIS2 ANAPC1 MERTK SERPINB1 MIR4645 LIMA1 ATP2C1 MIR7161 MVB12A TMEM221 ACYP2 COL7A1 UQCRC1 TMEM89 SLC26A6 MIR6824 CELSR3 MIR4793 LINC02585 MIR1226 CWC25 MIR4727 C17orf98 KIRREL1 GRAMD2A PKM CRLF1 TMEM59L MRPL23-AS1 LINC01219 HOTS MIR675 HDDC2 TTYH2 DNAI2 HIST3H2A

HIST3H2BB MIR4666A RNF187 MFSD9 TMEM182 SNORD62B SNORD62A POMT1 ARHGEF19 CPLANE2 MARK3 CTBP1 CTBP1-DT SPIRE1 PSMG2 GGT5 POM121L9P SPECC1L SPECC1L-ADORA2A PFKFB3 MIR3155A MIR3155B RAI1-AS1 SMCR5 SREBF1 MIR6777 MIR33B ARL5C CACNB1 RPL19 FAM87B LINC00115 LINC01128 DYNC1LI2 SLC9A8 LINC00235 CAPN15 KCNE1 VDAC2 COMTD1 ITGAD COX6A2 ZNF843 LINC00211 DPF1 PPP1R14A C19orf33 EZR EZR-AS1 OSTCP1 MSMB NCOA4 MIR544B MIR1302-4 MIR5003 SH2D7 KIAA1324 SYDE2 CCT6B ZNF830 LIG3 SENP6 LOC101926963 MKRN2OS MKRN2 MIR598 BICRA LINC00327 LATS2 GATM SPATA5L1 IER5L SMG6 PSMA1 PARVB HTRA4 TJAP1 LRRC73 YIPF3 POLR1C MIR1234 EMP2 SELENOH BTBD18 CTNND1 IL22RA1 CDCA5 ZFPL1 TMEM262 VPS51 TM7SF2 ZNHIT2 FAU MRPL49 SYVN1 MIR6751 HERC3 ZNF496 FAM200A STK32C LRRC27 KLF11 DYSF E2F6 RBMS1 CPNE9 REEP1 LOC100996693 GMPPA MIR3132 OBSL1 INHA SH3BP2 ADD1 POLM MIR6838 KCNE2 SMIM11A RBP7 UBE4B ZSWIM4 FOSB CCNY SH3BP5-AS1 AEBP1 MIR4649 POLD2 MYL7 GCK TRIM63 PDIK1L MIR151B MIR342 PGF TUSC8 XXYLT1 SP2-AS1 PNPO PRR15L CDK5RAP3 PSMD8 GGN SPRED3 FAM98C RASGRP4 RYR1 IQCD TPCN1 EFHD1 MYLK NEK9 TMED10 PLEKHG5 PLCL2 MIR3714 IFT46 ARCN1 DYNLRB1 MAP1LC3A GLB1L STK16 TUBA4A MIR3654 EPC2 MRPL33 GTSE1-DT GTSE1 LDB1 PPRC1 NOLC1 MIR6808 CCL17 CIAPIN1 COQ9 WDR38 RPL35 ARPC5L FAM83D DHX35 CCIN CLTA MIR152 IFRD1 RWDD4 TRAPPC11 ZNF678 LMF1 LMF1-AS1 SOX8 DDAH2 CLIC1 MSH5 MSH5-SAPCD1 PDP2 TADA1 LTBP1 MIR6820 RDH13 EPS8L1 NAA20 TMEM231 GABARAPL2 MFN2 MIIP MIR6729 TNFRSF8 LMOD1 TIMM17A RNPEP UIMC1 ZNF346 DCAKD NMT1 FSTL1 MYO18A HTRA3 ADAMTSL4-AS1 MCL1 LOC101927040 RHCE MACO1 TGFBR3 SMU1 SPECC1 COL8A2 POGLUT1 TIMMDC1 MRPL42P5 HSF2BP MIR6070 RRP1B CBX2 CBX8 BCL2 KDSR SHISAL1 SLC30A5 ALDOB CCDC112 PSD3 NAALADL1 QRICH2 KCNK3 DNM1P46 ARHGEF7 ARHGEF7-AS1 LOC101060553 CYS1 GYG1 USP36 JAK1 LINC01359 UROS MIR4484 BCCIP SLC45A4
- Timestamp used as random seed: 1593882878379

GSEA Report for Dataset RNAseq_genes

Enrichment in phenotype: up (1 samples)

- None of the gene sets are enriched in phenotype **up**
- Guide to interpret results

Enrichment in phenotype: down (1 samples)

- 1 / 1 gene sets are upregulated in phenotype **down**
- 1 gene sets are significantly enriched at FDR < 25%
- 0 gene sets are significantly enriched at nominal pvalue < 1%
- 1 gene sets are significantly enriched at nominal pvalue < 5%
- Snapshot of enrichment results
- Detailed enrichment results in html format
- Detailed enrichment results in excel format (tab delimited text)
- Guide to interpret results

Dataset details

- The dataset has 2033 features (genes)
- No probe set => gene symbol collapsing was requested, so all 2033 features were used

Gene set details

- Gene set size filters (min=1, max=500) resulted in filtering out 0 / 1 gene sets
- The remaining 1 gene sets were used in the analysis
- List of gene sets used and their sizes (restricted to features in the specified dataset)

Gene markers for the up *versus* down comparison

- The dataset has 2033 features (genes)
- # of markers for phenotype **up**: 936 (46.0%) with correlation area 47.0%
- # of markers for phenotype **down**: 1097 (54.0%) with correlation area 53.0%
- Detailed rank ordered gene list for all features in the dataset
- Heat map and gene list correlation profile for all features in the dataset

Global statistics and plots

- Plot of p-values *vs.* NES
- Global ES histogram

Other

- Parameters used for this analysis

Comments

- There were duplicate row identifiers in the specified dataset. One id was arbitarilly choosen. Details are below Generally, this is OK, but if you want to avoid this automagic, edit your dataset so that all row ids are unique # of

row ids in original dataset: 2035 # of row UNIQUE ids in original dataset: 2033 # The duplicates were ZC3H11A TSTD3
- Timestamp used as random seed: 1593882900857

---

xtools.gsea.Gsea [Sat, Jul 4, '20 7 PM 15] Report: Metabolism_RNA_hallmark.Gsea.1593882900841.rpt by user: Jiayi
Website: www.gsea-msigdb.org/gsea Questions & Suggestions: Contact page

GSEA Report for Dataset Proteome

Enrichment in phenotype: up (1 samples)

- None of the gene sets are enriched in phenotype **up**
- Guide to interpret results

Enrichment in phenotype: down (1 samples)

- 1 / 1 gene sets are upregulated in phenotype **down**
- 1 gene sets are significantly enriched at FDR < 25%
- 1 gene sets are significantly enriched at nominal pvalue < 1%
- 1 gene sets are significantly enriched at nominal pvalue < 5%
- Snapshot of enrichment results
- Detailed enrichment results in html format
- Detailed enrichment results in excel format (tab delimited text)
- Guide to interpret results

Dataset details

- The dataset has 432 features (genes)
- No probe set => gene symbol collapsing was requested, so all 432 features were used

Gene set details

- Gene set size filters (min=1, max=500) resulted in filtering out 0 / 1 gene sets
- The remaining 1 gene sets were used in the analysis
- List of gene sets used and their sizes (restricted to features in the specified dataset)

Gene markers for the up *versus* down comparison

- The dataset has 432 features (genes)
- # of markers for phenotype **up**: 209 (48.4%) with correlation area 50.7%
- # of markers for phenotype **down**: 223 (51.6%) with correlation area 49.3%
- Detailed rank ordered gene list for all features in the dataset
- Heat map and gene list correlation profile for all features in the dataset

Global statistics and plots

- Plot of p-values *vs.* NES
- Global ES histogram

Other

- Parameters used for this analysis

Comments

- There were duplicate row identifiers in the specified dataset. One id was arbitarilly choosen. Details are below Generally, this is OK, but if you want to avoid this automagic, edit your dataset so that all row ids are unique # of

row ids in original dataset: 441 # of row UNIQUE ids in original dataset: 432 # The duplicates were POSTN POSTN MAP4 POSTN VCAN PDLIM5 FBLN1 PKM PALLD

- Timestamp used as random seed: 1593882854273

---

xtools.gsea.Gsea [Sat, Jul 4, '20 7 PM 14] Report: Metabolism_protein_hallmark.Gsea.1593882854266.rpt by user: Jiayi
Website: www.gsea-msigdb.org/gsea Questions & Suggestions: Contact page
