# Supplementary material for: Multi-omics integration identifies key upstream regulators of pathomechanisms in hypertrophic cardiomyopathy due to truncating MYBPC3 mutations
Source: Clin Epigenetics. 2021 Mar 23;13:61. doi: 10.1186/s13148-021-01043-3 (PMC7989210; doi:10.1186/s13148-021-01043-3)
Supplement: Supplementary file 4 — Additional file 4: Figure S4 (A) Enriched transcription factor binding motifs in the hyperacetylated regions in HCM versus control hearts. (B) Enriched transcription factor binding motifs in the hypoacetylated regions in HCM versus control hearts [file 13148_2021_1043_MOESM4_ESM.pdf]

Supplementary Figure 4A

AME

Analysis of Motif Enrichment

For further information on how to interpret these results please access http://meme-suite.org//doc/ame-output-format.html.
To get a copy of the MEME software please access http://meme-suite.org.

If you use AME in your research, please cite the following paper:
Robert McLeay and Timothy L. Bailey, "Motif Enrichment Analysis: A unified framework and method evaluation", *BMC Bioinformatics*, **11**:165, 2010, doi:10.1186/1471-2105-11-165. [full text]

Enriched Motifs | Input Files | Program information | Results in TSV Format ?? NEW | Positive sequences for each motif ? NEW | Positive sequences for each motif ? NEW

Enriched Motifs

Sequence motif score: avg_odds
Background model source: letter frequencies in (primary) sequences file (Galaxy195-bedtools_GetFastaBed_Merged_and_Narrowed_Hyper_regions.fasta)
Background model frequencies: 0.206554,0.293446,0.293446,0.206554
Total pseudocount added to a motif column: 0.1

Statistical test: Fisher's exact test (optimized over motif scores)
Labeling positives: all 107 primary sequences are labeled as 'positive'; all 1070 control sequences are labeled as 'negative'
Classifying positives: sequences with the motif scores ≥ 'TP Thresh' are classified as positive

E-value threshold for reporting results: 10
Sequence motif score: avg_odds
Background model source: letter frequencies in (primary) sequences file (Galaxy195-bedtools_GetFastaBed_Merged_and_Narrowed_Hyper_regions.fasta)
Background model frequencies: 0.206554,0.293446,0.293446,0.206554
Total pseudocount added to a motif column: 0.1

Statistical test: Fisher's exact test (optimized over motif scores)
Labeling positives: all 107 primary sequences are labeled as 'positive'; all 1070 control sequences are labeled as 'negative'
Classifying positives: sequences with the motif scores ≥ 'TP Thresh' are classified as positive

E-value threshold for reporting results: 10

Logo	Database ?	ID ?	Alt ID ?	*p*-value ?	*E*-value ?	TP Thresh ?	TP (%)

Logo	Database	ID	Alt ID	*p*-value	*E*-value	TP Thresh	TP (%)
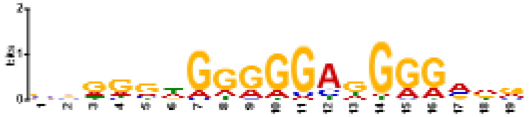	HOCOMOCOv11 full HUMAN mono meme format	ZBT17_HUMAN.H11MO.0.A		1.64e-13	1.26e-10	7.95	55 (51.4%
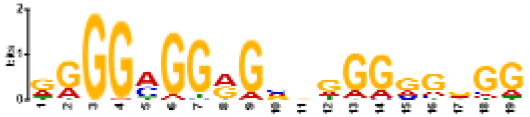	HOCOMOCOv11 full HUMAN mono meme format	KLF15_HUMAN.H11MO.0.A		2.26e-11	1.74e-8	13.14	42 (39.3%
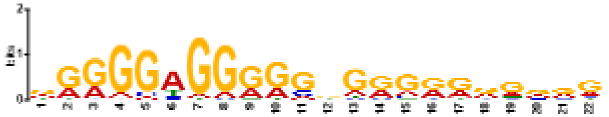	HOCOMOCOv11 full HUMAN mono meme format	MAZ_HUMAN.H11MO.0.A		6.47e-11	4.97e-8	31.26	41 (38.3%
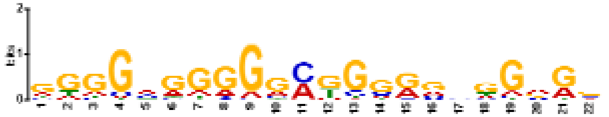	HOCOMOCOv11 full HUMAN mono meme format	PATZ1_HUMAN.H11MO.0.C		1.12e-10	8.59e-8	5.67	55 (51.4%
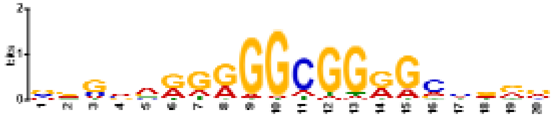	HOCOMOCOv11 full HUMAN mono meme format	SP4_HUMAN.H11MO.0.A		1.23e-10	9.48e-8	3.19	55 (51.4%
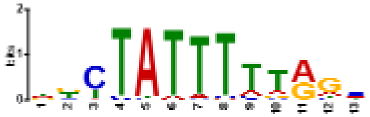	HOCOMOCOv11 full HUMAN mono meme format	MEF2C_HUMAN.H11MO.0.A		1.98e-10	1.53e-7	19.97	16 (15.0%
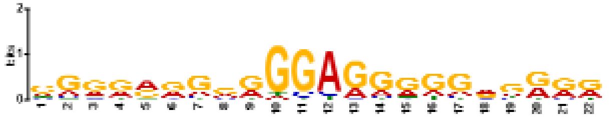	HOCOMOCOv11 full HUMAN mono meme format	VEZF1_HUMAN.H11MO.0.C		2.02e-10	1.55e-7	7.08	59 (55.1%
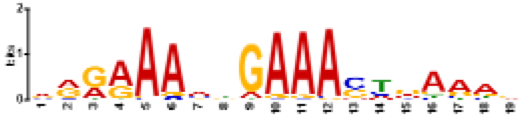	HOCOMOCOv11 full HUMAN mono meme format	STAT1_HUMAN.H11MO.1.A		6.28e-10	4.83e-7	2.27	51 (47.7%
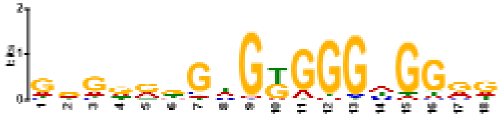	HOCOMOCOv11 full HUMAN mono meme format	EGR2_HUMAN.H11MO.0.A		1.10e-9	8.47e-7	7.36	46 (43.0%
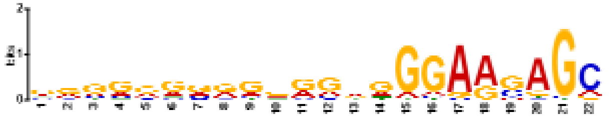	HOCOMOCOv11 full HUMAN mono meme format	ZN341_HUMAN.H11MO.0.C		2.54e-9	1.95e-6	6.71	50 (46.7%

Logo	Database	ID	Alt ID	*p*-value	*E*-value	TP Thresh	TP (%)
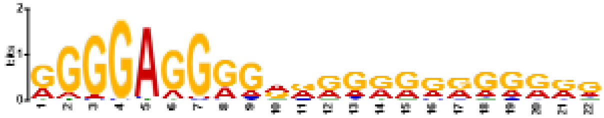	HOCOMOCOv11 full HUMAN mono meme format	ZN467_HUMAN.H11MO.0.C		3.44e-9	2.64e-6	4.21	56 (52.3%
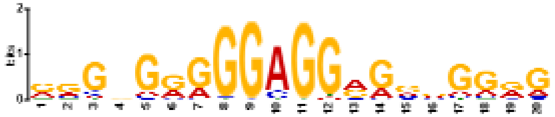	HOCOMOCOv11 full HUMAN mono meme format	WT1_HUMAN.H11MO.0.C		3.83e-9	2.94e-6	4.81	50 (46.7%
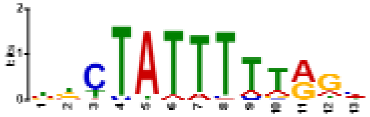	HOCOMOCOv11 full HUMAN mono meme format	MEF2A_HUMAN.H11MO.0.A		4.74e-9	3.65e-6	16.25	17 (15.9%
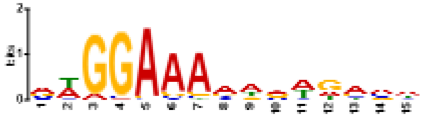	HOCOMOCOv11 full HUMAN mono meme format	NFAC1_HUMAN.H11MO.0.B		5.95e-9	4.57e-6	2.22	65 (60.7%
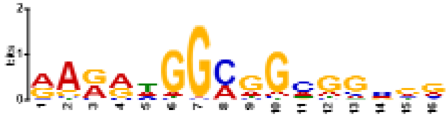	HOCOMOCOv11 full HUMAN mono meme format	TAF1_HUMAN.H11MO.0.A		8.36e-9	6.43e-6	2.11	51 (47.7%
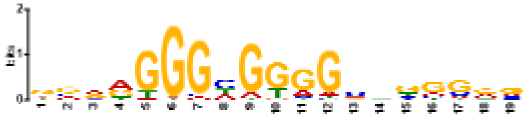	HOCOMOCOv11 full HUMAN mono meme format	KLF6_HUMAN.H11MO.0.A		1.09e-8	8.38e-6	4.00	51 (47.7%
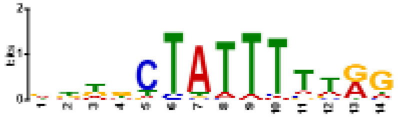	HOCOMOCOv11 full HUMAN mono meme format	MEF2B_HUMAN.H11MO.0.A		1.32e-8	1.02e-5	4.68	31 (29.0%
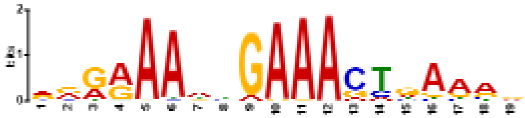	HOCOMOCOv11 full HUMAN mono meme format	STAT2_HUMAN.H11MO.0.A		2.52e-8	1.94e-5	2.42	34 (31.8%
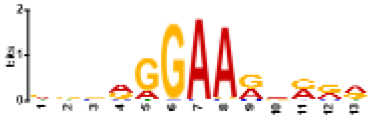	HOCOMOCOv11 full HUMAN mono meme format	ETS2_HUMAN.H11MO.0.B		4.72e-8	3.63e-5	2.95	69 (64.5%
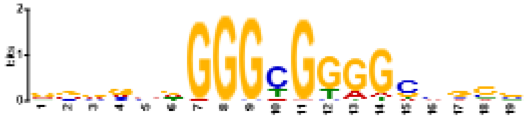	HOCOMOCOv11 full HUMAN mono meme format	KLF3_HUMAN.H11MO.0.B		6.44e-8	4.96e-5	7.68	29 (27.1%

Logo	Database	ID	Alt ID	*p*-value	*E*-value	TP Thresh	TP (%)
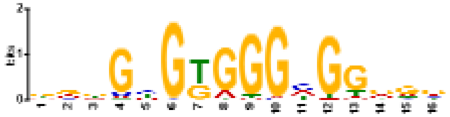	HOCOMOCOv11 full HUMAN mono meme format	EGR2_HUMAN.H11MO.1.A		9.68e-8	7.45e-5	10.60	28 (26.2%
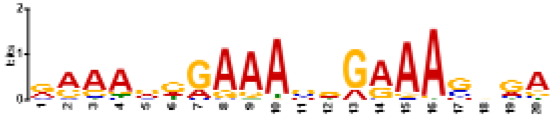	HOCOMOCOv11 full HUMAN mono meme format	IRF3_HUMAN.H11MO.0.B		1.06e-7	8.16e-5	1.35	63 (58.9%
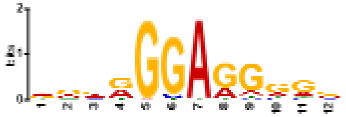	HOCOMOCOv11 full HUMAN mono meme format	VEZF1_HUMAN.H11MO.1.C		1.21e-7	9.27e-5	7.03	40 (37.4%
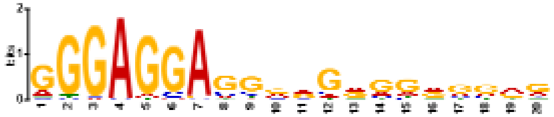	HOCOMOCOv11 full HUMAN mono meme format	ZN263_HUMAN.H11MO.0.A		1.30e-7	9.96e-5	3.29	53 (49.5%
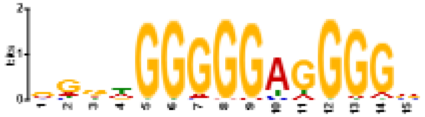	HOCOMOCOv11 full HUMAN mono meme format	ZN281_HUMAN.H11MO.0.A		1.34e-7	1.03e-4	33.95	26 (24.3%
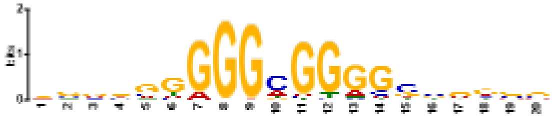	HOCOMOCOv11 full HUMAN mono meme format	SP3_HUMAN.H11MO.0.B		1.97e-7	1.52e-4	8.94	33 (30.8%
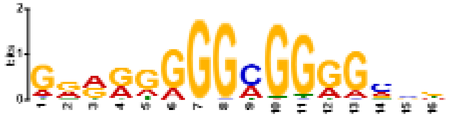	HOCOMOCOv11 full HUMAN mono meme format	SP4_HUMAN.H11MO.1.A		2.81e-7	2.16e-4	4.47	41 (38.3%
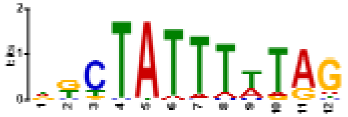	HOCOMOCOv11 full HUMAN mono meme format	MEF2D_HUMAN.H11MO.0.A		6.42e-7	4.94e-4	2.24	25 (23.4%
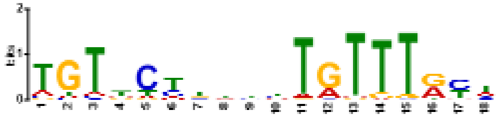	HOCOMOCOv11 full HUMAN mono meme format	ANDR_HUMAN.H11MO.0.A		9.90e-7	7.61e-4	17.86	18 (16.8%
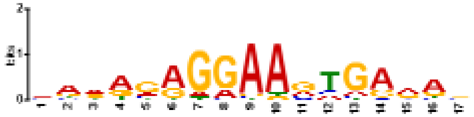	HOCOMOCOv11 full HUMAN mono meme format	BC11A_HUMAN.H11MO.0.A		1.05e-6	8.04e-4	8.60	36 (33.6%

Logo	Database	ID	Alt ID	*p*-value	*E*-value	TP Thresh	TP (%)
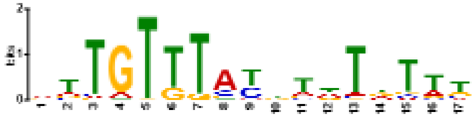	HOCOMOCOv11 full HUMAN mono meme format	FOXG1_HUMAN.H11MO.0.D		1.45e-6	1.11e-3	7.05	26 (24.3%
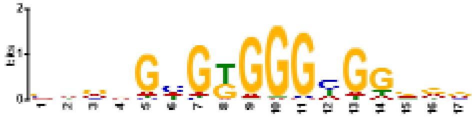	HOCOMOCOv11 full HUMAN mono meme format	EGR1_HUMAN.H11MO.0.A		1.68e-6	1.29e-3	3.97	37 (34.6%
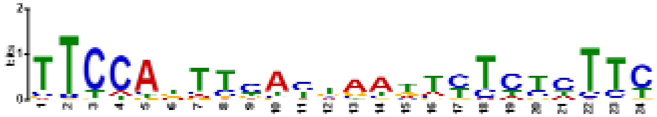	HOCOMOCOv11 full HUMAN mono meme format	ZFP82_HUMAN.H11MO.0.C		1.89e-6	1.45e-3	2.69	35 (32.7%
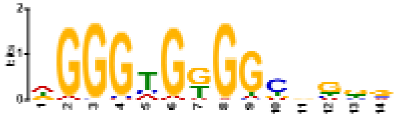	HOCOMOCOv11 full HUMAN mono meme format	KLF5_HUMAN.H11MO.0.A		2.37e-6	1.82e-3	6.48	34 (31.8%
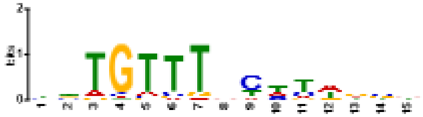	HOCOMOCOv11 full HUMAN mono meme format	FOXD3_HUMAN.H11MO.0.D		5.73e-6	4.40e-3	1.71	61 (57.0%
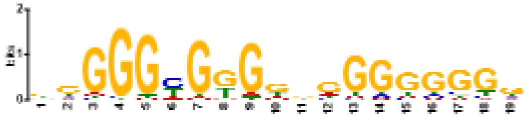	HOCOMOCOv11 full HUMAN mono meme format	KLF16_HUMAN.H11MO.0.D		9.19e-6	7.07e-3	14.26	25 (23.4%
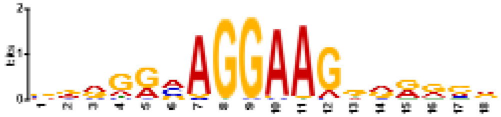	HOCOMOCOv11 full HUMAN mono meme format	FLI1_HUMAN.H11MO.0.A		9.44e-6	7.26e-3	9.07	38 (35.5%
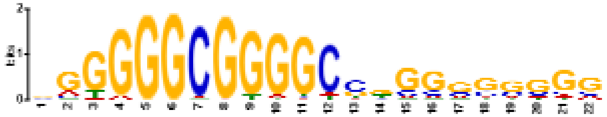	HOCOMOCOv11 full HUMAN mono meme format	SP1_HUMAN.H11MO.0.A		1.03e-5	7.94e-3	29.04	15 (14.0%
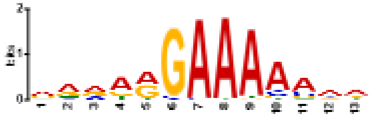	HOCOMOCOv11 full HUMAN mono meme format	PRDM6_HUMAN.H11MO.0.C		1.11e-5	8.55e-3	2.60	55 (51.4%
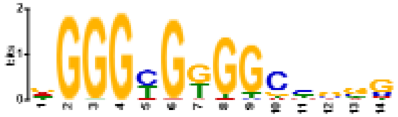	HOCOMOCOv11 full HUMAN mono meme format	KLF1_HUMAN.H11MO.0.A		1.65e-5	1.27e-2	13.18	19 (17.8%

Logo	Database ?	ID ?	Alt ID ?	*p*-value ?	*E*-value ?	TP Thresh ?	TP (%)
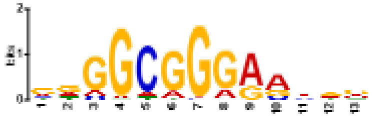	HOCOMOCOv11 full HUMAN mono meme format	E2F7_HUMAN.H11MO.0.B		1.71e-5	1.31e-2	2.28	47 (43.9%
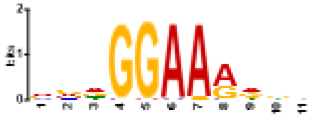	HOCOMOCOv11 full HUMAN mono meme format	NFAT5_HUMAN.H11MO.0.D		1.86e-5	1.43e-2	2.61	66 (61.7%
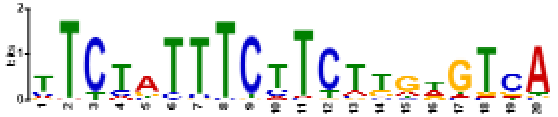	HOCOMOCOv11 full HUMAN mono meme format	ZFP28_HUMAN.H11MO.0.C		2.07e-5	1.59e-2	2.05	21 (19.6%
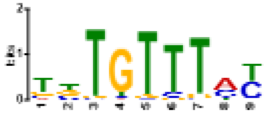	HOCOMOCOv11 full HUMAN mono meme format	FOXP1_HUMAN.H11MO.0.A		2.10e-5	1.61e-2	1.53	54 (50.5%
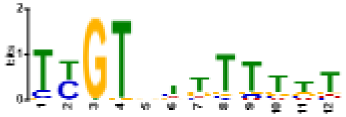	HOCOMOCOv11 full HUMAN mono meme format	FUBP1_HUMAN.H11MO.0.D		2.34e-5	1.80e-2	4.37	28 (26.2%
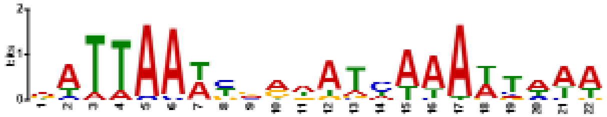	HOCOMOCOv11 full HUMAN mono meme format	ARI3A_HUMAN.H11MO.0.D		3.21e-5	2.47e-2	1.32	17 (15.9%
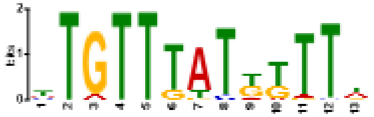	HOCOMOCOv11 full HUMAN mono meme format	FOXJ3_HUMAN.H11MO.0.A		3.36e-5	2.58e-2	3.95	17 (15.9%
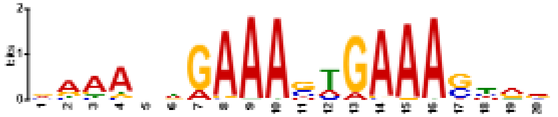	HOCOMOCOv11 full HUMAN mono meme format	IRF1_HUMAN.H11MO.0.A		3.64e-5	2.80e-2	20.19	16 (15.0%
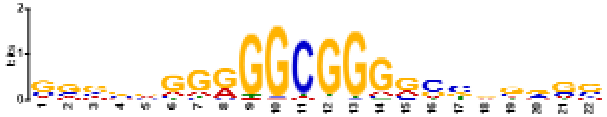	HOCOMOCOv11 full HUMAN mono meme format	SP2_HUMAN.H11MO.0.A		3.80e-5	2.92e-2	3.56	38 (35.5%
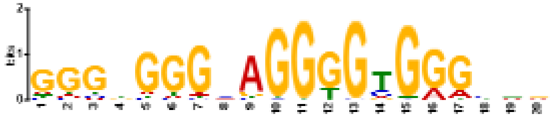	HOCOMOCOv11 full HUMAN mono meme format	TBX1_HUMAN.H11MO.0.D		4.09e-5	3.14e-2	3.07	35 (32.7%

Logo	Database	ID	Alt ID	p-value	E-value	TP Thresh	TP (%)
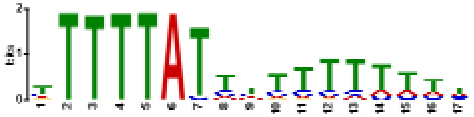	HOCOMOCOv11 full HUMAN mono meme format	CPEB1_HUMAN.H11MO.0.D		4.30e-5	3.31e-2	1.26	22 (20.6%
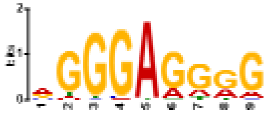	HOCOMOCOv11 full HUMAN mono meme format	PATZ1_HUMAN.H11MO.1.C		4.34e-5	3.33e-2	5.24	43 (40.2%
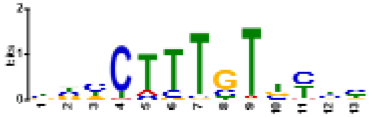	HOCOMOCOv11 full HUMAN mono meme format	SOX10_HUMAN.H11MO.1.A		4.57e-5	3.52e-2	3.44	52 (48.6%
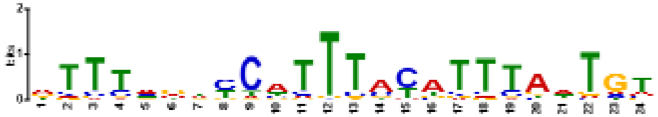	HOCOMOCOv11 full HUMAN mono meme format	Z354A_HUMAN.H11MO.0.C		4.58e-5	3.52e-2	2.62	29 (27.1%
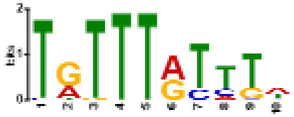	HOCOMOCOv11 full HUMAN mono meme format	FOXJ2_HUMAN.H11MO.0.C		5.68e-5	4.37e-2	1.04	33 (30.8%
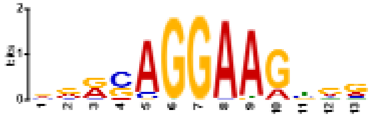	HOCOMOCOv11 full HUMAN mono meme format	ERG_HUMAN.H11MO.0.A		5.89e-5	4.53e-2	1.79	57 (53.3%
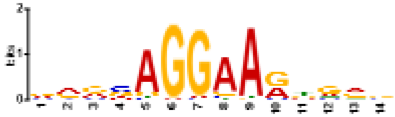	HOCOMOCOv11 full HUMAN mono meme format	ETV5_HUMAN.H11MO.0.C		5.96e-5	4.59e-2	2.91	62 (57.9%
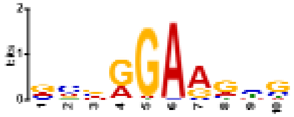	HOCOMOCOv11 full HUMAN mono meme format	FEV_HUMAN.H11MO.0.B		7.52e-5	5.78e-2	1.60	61 (57.0%
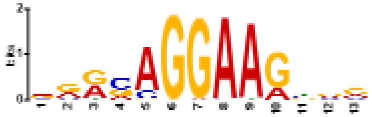	HOCOMOCOv11 full HUMAN mono meme format	FLI1_HUMAN.H11MO.1.A		9.36e-5	7.19e-2	1.87	56 (52.3%
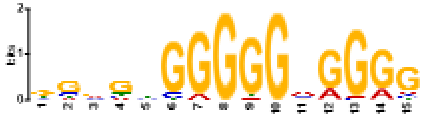	HOCOMOCOv11 full HUMAN mono meme format	ZN148_HUMAN.H11MO.0.D		1.15e-4	8.87e-2	19.62	27 (25.2%

Logo	Database	ID	Alt ID	*p*-value	*E*-value	TP Thresh	TP (%)
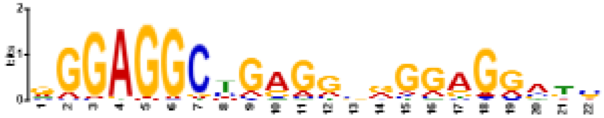	HOCOMOCOv11 full HUMAN mono meme format	ZN770_HUMAN.H11MO.0.C		1.27e-4	9.76e-2	1.13	53 (49.5%
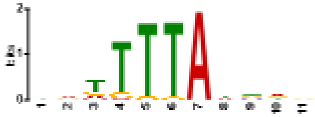	HOCOMOCOv11 full HUMAN mono meme format	HXD12_HUMAN.H11MO.0.D		1.31e-4	1.01e-1	1.59	45 (42.1%
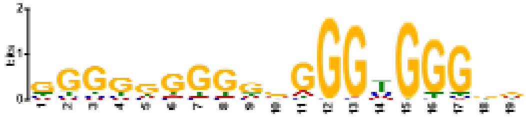	HOCOMOCOv11 full HUMAN mono meme format	TBX15_HUMAN.H11MO.0.D		1.34e-4	1.03e-1	3.09	48 (44.9%
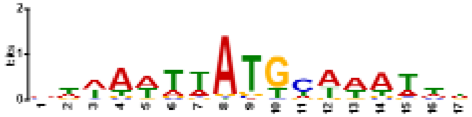	HOCOMOCOv11 full HUMAN mono meme format	PO3F3_HUMAN.H11MO.0.D		1.35e-4	1.04e-1	14.90	16 (15.0%
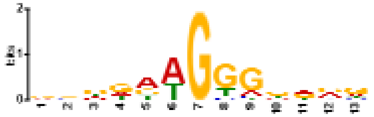	HOCOMOCOv11 full HUMAN mono meme format	SPIC_HUMAN.H11MO.0.D		1.48e-4	1.14e-1	2.81	51 (47.7%
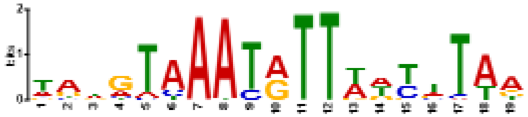	HOCOMOCOv11 full HUMAN mono meme format	FOXD2_HUMAN.H11MO.0.D		1.56e-4	1.20e-1	1.63	9 (8.4%)
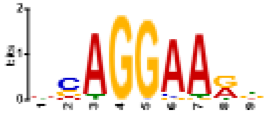	HOCOMOCOv11 full HUMAN mono meme format	ETV6_HUMAN.H11MO.0.D		2.01e-4	1.54e-1	2.86	55 (51.4%
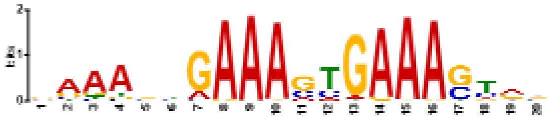	HOCOMOCOv11 full HUMAN mono meme format	IRF2_HUMAN.H11MO.0.A		2.25e-4	1.73e-1	7.92	16 (15.0%
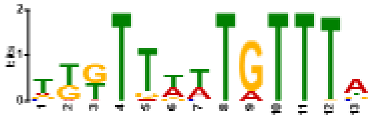	HOCOMOCOv11 full HUMAN mono meme format	FOXJ3_HUMAN.H11MO.1.B		2.47e-4	1.90e-1	1.18	16 (15.0%
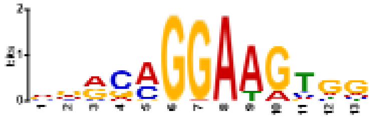	HOCOMOCOv11 full HUMAN mono meme format	ETS1_HUMAN.H11MO.0.A		3.16e-4	2.43e-1	1.91	41 (38.3%

Logo	Database ?	ID ?	Alt ID ?	*p*-value ?	*E*-value ?	TP Thresh ?	TP (%)
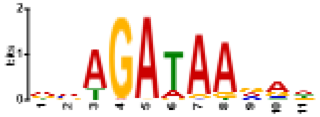	HOCOMOCOv11 full HUMAN mono meme format	GATA3_HUMAN.H11MO.0.A		4.13e-4	3.18e-1	1.11	59 (55.1%
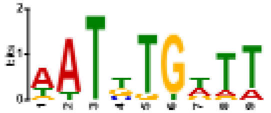	HOCOMOCOv11 full HUMAN mono meme format	FOXP3_HUMAN.H11MO.0.D		4.13e-4	3.18e-1	1.02	49 (45.8%
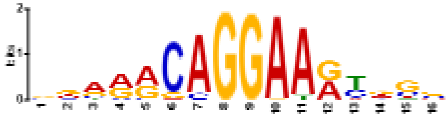	HOCOMOCOv11 full HUMAN mono meme format	ETV2_HUMAN.H11MO.0.B		5.24e-4	4.03e-1	1.23	40 (37.4%
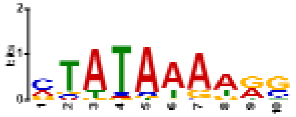	HOCOMOCOv11 full HUMAN mono meme format	TBP_HUMAN.H11MO.0.A		5.26e-4	4.04e-1	8.70	11 (10.3%
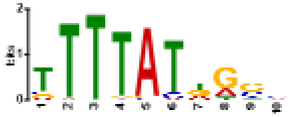	HOCOMOCOv11 full HUMAN mono meme format	HXB13_HUMAN.H11MO.0.A		5.48e-4	4.21e-1	1.27	37 (34.6%
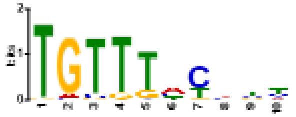	HOCOMOCOv11 full HUMAN mono meme format	FOXK1_HUMAN.H11MO.0.A		5.71e-4	4.39e-1	1.11	64 (59.8%
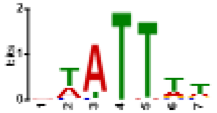	HOCOMOCOv11 full HUMAN mono meme format	HMGA1_HUMAN.H11MO.0.D		6.51e-4	5.00e-1	1.41	55 (51.4%
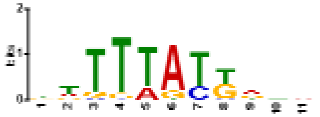	HOCOMOCOv11 full HUMAN mono meme format	HXD11_HUMAN.H11MO.0.D		6.54e-4	5.03e-1	1.06	52 (48.6%
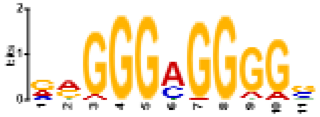	HOCOMOCOv11 full HUMAN mono meme format	MAZ_HUMAN.H11MO.1.A		6.68e-4	5.13e-1	5.74	40 (37.4%
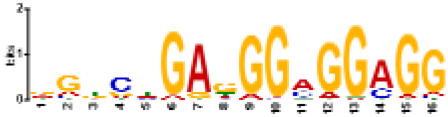	HOCOMOCOv11 full HUMAN mono meme format	ZSC22_HUMAN.H11MO.0.C		6.75e-4	5.19e-1	1.63	47 (43.9%

Logo	Database	ID	Alt ID	p-value	E-value	TP Thresh	TP (%)
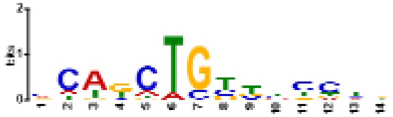	HOCOMOCOv11 full HUMAN mono meme format	LYL1_HUMAN.H11MO.0.A		6.77e-4	5.20e-1	1.86	67 (62.6%
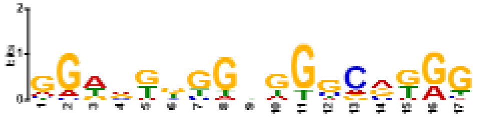	HOCOMOCOv11 full HUMAN mono meme format	PURA_HUMAN.H11MO.0.D		9.21e-4	7.08e-1	5.09	30 (28.0%
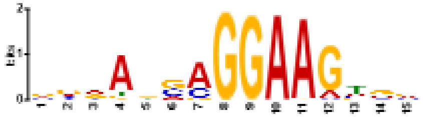	HOCOMOCOv11 full HUMAN mono meme format	ELF5_HUMAN.H11MO.0.A		9.96e-4	7.66e-1	1.16	53 (49.5%
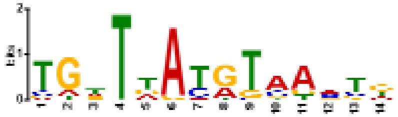	HOCOMOCOv11 full HUMAN mono meme format	TEF_HUMAN.H11MO.0.D		1.08e-3	8.32e-1	2.64	17 (15.9%
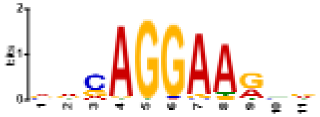	HOCOMOCOv11 full HUMAN mono meme format	ETV4_HUMAN.H11MO.0.B		1.13e-3	8.73e-1	2.84	56 (52.3%
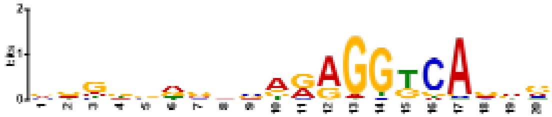	HOCOMOCOv11 full HUMAN mono meme format	RXRA_HUMAN.H11MO.0.A		1.20e-3	9.21e-1	2.11	67 (62.6%
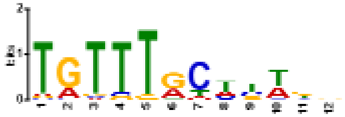	HOCOMOCOv11 full HUMAN mono meme format	FOXM1_HUMAN.H11MO.0.A		1.21e-3	9.32e-1	2.95	32 (29.9%
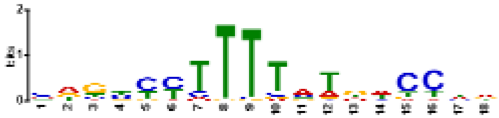	HOCOMOCOv11 full HUMAN mono meme format	ZN350_HUMAN.H11MO.0.C		1.23e-3	9.44e-1	1.25	61 (57.0%
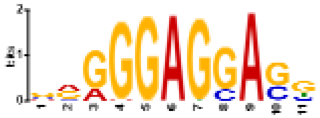	HOCOMOCOv11 full HUMAN mono meme format	ZN263_HUMAN.H11MO.1.A		1.26e-3	9.71e-1	5.52	37 (34.6%
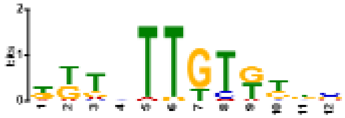	HOCOMOCOv11 full HUMAN mono meme format	BPTF_HUMAN.H11MO.0.D		1.45e-3	1.11e0	4.96	27 (25.2%

Logo	Database	ID	Alt ID	*p*-value	*E*-value	TP Thresh	TP (%)
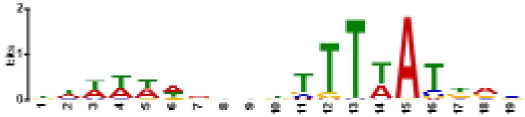	HOCOMOCOv11 full HUMAN mono meme format	HXC10_HUMAN.H11MO.0.D		1.50e-3	1.15e0	3.54	27 (25.2%
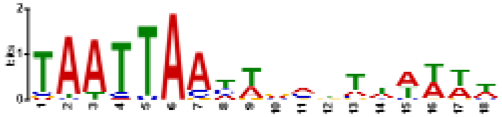	HOCOMOCOv11 full HUMAN mono meme format	LMX1B_HUMAN.H11MO.0.D		1.63e-3	1.25e0	3.53	16 (15.0%
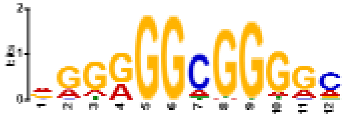	HOCOMOCOv11 full HUMAN mono meme format	SP2_HUMAN.H11MO.1.B		1.71e-3	1.31e0	2.57	40 (37.4%
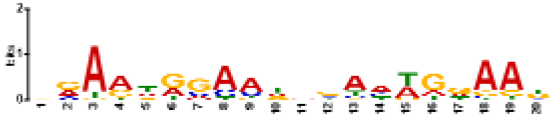	HOCOMOCOv11 full HUMAN mono meme format	ZN394_HUMAN.H11MO.0.C		2.13e-3	1.64e0	1.58	63 (58.9%
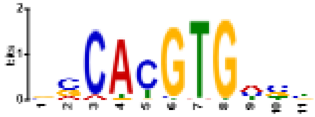	HOCOMOCOv11 full HUMAN mono meme format	BMAL1_HUMAN.H11MO.0.A		2.36e-3	1.81e0	1.39	25 (23.4%
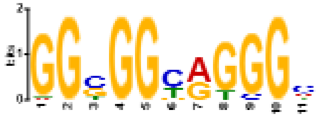	HOCOMOCOv11 full HUMAN mono meme format	EGR4_HUMAN.H11MO.0.D		2.49e-3	1.91e0	5.58	21 (19.6%
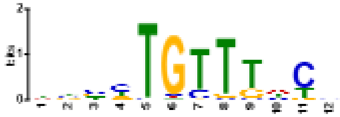	HOCOMOCOv11 full HUMAN mono meme format	FOXO1_HUMAN.H11MO.0.A		2.68e-3	2.06e0	1.39	63 (58.9%
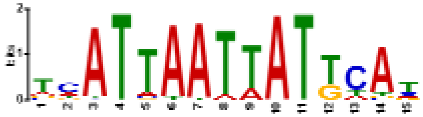	HOCOMOCOv11 full HUMAN mono meme format	PO4F3_HUMAN.H11MO.0.D		2.81e-3	2.16e0	2.79	7 (6.5%)
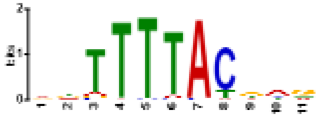	HOCOMOCOv11 full HUMAN mono meme format	HXC13_HUMAN.H11MO.0.D		3.04e-3	2.34e0	1.49	30 (28.0%
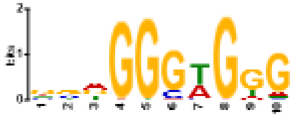	HOCOMOCOv11 full HUMAN mono meme format	SALL4_HUMAN.H11MO.0.B		3.14e-3	2.42e0	6.10	28 (26.2%

Logo	Database	ID	Alt ID	*p*-value	*E*-value	TP Thresh	TP (%)
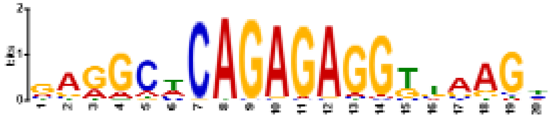	HOCOMOCOv11 full HUMAN mono meme format	ZN768_HUMAN.H11MO.0.C		3.61e-3	2.78e0	9.17	14 (13.1%
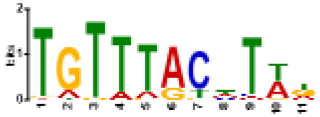	HOCOMOCOv11 full HUMAN mono meme format	FOXF2_HUMAN.H11MO.0.D		3.88e-3	2.98e0	1.37	29 (27.1%
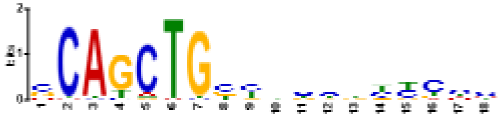	HOCOMOCOv11 full HUMAN mono meme format	PTF1A_HUMAN.H11MO.0.B		4.16e-3	3.20e0	1.25	60 (56.1%
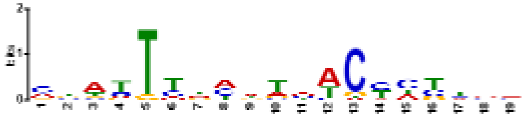	HOCOMOCOv11 full HUMAN mono meme format	MNX1_HUMAN.H11MO.0.D		4.49e-3	3.45e0	3.59	35 (32.7%
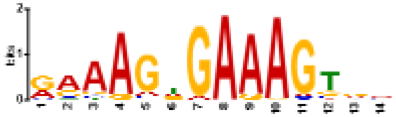	HOCOMOCOv11 full HUMAN mono meme format	PRDM1_HUMAN.H11MO.0.A		5.34e-3	4.10e0	1.32	45 (42.1%
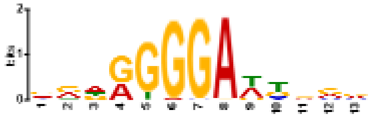	HOCOMOCOv11 full HUMAN mono meme format	MZF1_HUMAN.H11MO.0.B		5.91e-3	4.54e0	5.41	26 (24.3%
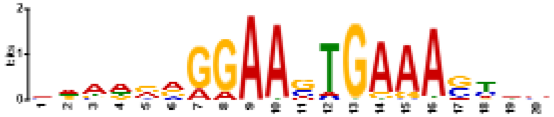	HOCOMOCOv11 full HUMAN mono meme format	IRF8_HUMAN.H11MO.0.B		6.06e-3	4.66e0	2.05	25 (23.4%
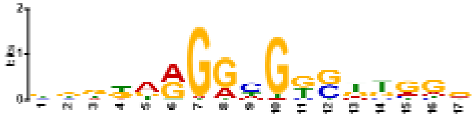	HOCOMOCOv11 full HUMAN mono meme format	PROX1_HUMAN.H11MO.0.D		6.07e-3	4.67e0	2.81	35 (32.7%
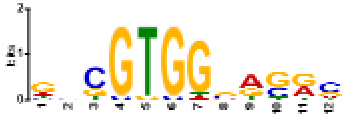	HOCOMOCOv11 full HUMAN mono meme format	ARNT2_HUMAN.H11MO.0.D		6.21e-3	4.78e0	1.02	48 (44.9%
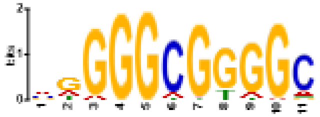	HOCOMOCOv11 full HUMAN mono meme format	KLF12_HUMAN.H11MO.0.C		6.28e-3	4.83e0	5.87	18 (16.8%

Logo	Database	ID	Alt ID	*p*-value	*E*-value	TP Thresh	TP (%)
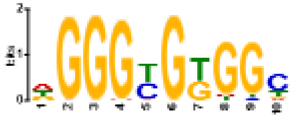	HOCOMOCOv11 full HUMAN mono meme format	KLF4_HUMAN.H11MO.0.A		6.41e-3	4.93e0	2.81	32 (29.9%
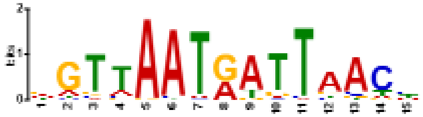	HOCOMOCOv11 full HUMAN mono meme format	HNF1B_HUMAN.H11MO.0.A		6.44e-3	4.95e0	1.46	19 (17.8%
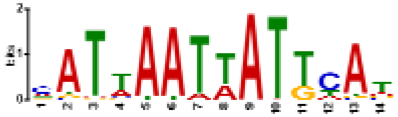	HOCOMOCOv11 full HUMAN mono meme format	PO4F1_HUMAN.H11MO.0.D		6.54e-3	5.03e0	10.25	6 (5.6%)
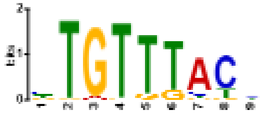	HOCOMOCOv11 full HUMAN mono meme format	FOXP2_HUMAN.H11MO.0.C		7.15e-3	5.50e0	1.58	36 (33.6%
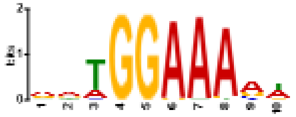	HOCOMOCOv11 full HUMAN mono meme format	NFAC1_HUMAN.H11MO.1.B		7.23e-3	5.56e0	2.96	36 (33.6%
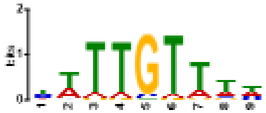	HOCOMOCOv11 full HUMAN mono meme format	SRY_HUMAN.H11MO.0.B		7.81e-3	6.00e0	1.18	53 (49.5%
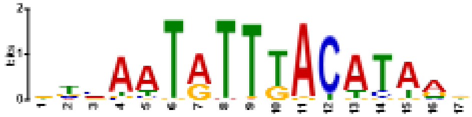	HOCOMOCOv11 full HUMAN mono meme format	FOXB1_HUMAN.H11MO.0.D		8.80e-3	6.76e0	4.49	8 (7.5%)
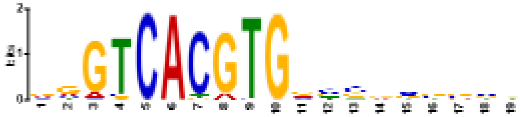	HOCOMOCOv11 full HUMAN mono meme format	USF2_HUMAN.H11MO.0.A		1.12e-2	8.60e0	6.31	7 (6.5%)
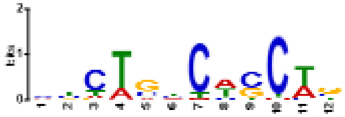	HOCOMOCOv11 full HUMAN mono meme format	SMAD3_HUMAN.H11MO.0.B		1.15e-2	8.82e0	2.76	57 (53.3%
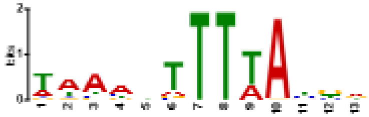	HOCOMOCOv11 full HUMAN mono meme format	HXA11_HUMAN.H11MO.0.D		1.18e-2	9.04e0	2.23	28 (26.2%

Logo	Database	ID	Alt ID	*p*-value	*E*-value	TP Thresh	TP (%)
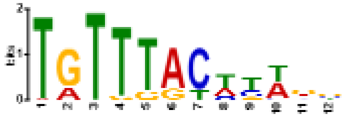	HOCOMOCOv11 full HUMAN mono meme format	FOXA1_HUMAN.H11MO.0.A		1.21e-2	9.30e0	1.04	32 (29.9%
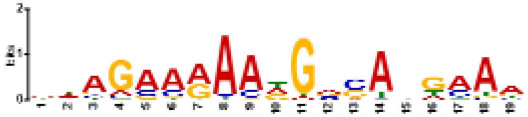	HOCOMOCOv11 full HUMAN mono meme format	ZN713_HUMAN.H11MO.0.D		1.22e-2	9.38e0	2.57	37 (34.6%
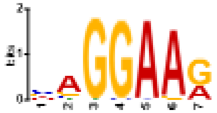	HOCOMOCOv11 full HUMAN mono meme format	ETV7_HUMAN.H11MO.0.D		1.25e-2	9.58e0	3.31	45 (42.1%
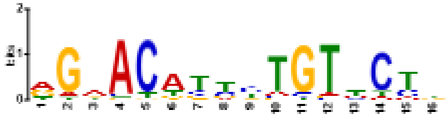	HOCOMOCOv11 full HUMAN mono meme format	PRGR_HUMAN.H11MO.0.A		1.29e-2	9.94e0	1.14	41 (38.3%
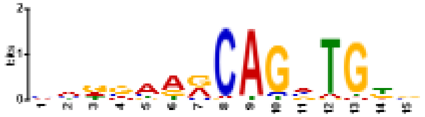	HOCOMOCOv11 full HUMAN mono meme format	TAL1_HUMAN.H11MO.1.A		1.30e-2	9.99e0	2.31	44 (41.1%

Input Files

Alphabet

Background source: built from the (primary) sequences

Background source: built from the (primary) sequences

Name	Bg.				Bg.	Name
Adenine	0.206554	A	~	T	0.206554	Thymine
Cytosine	0.293446	C	~	G	0.293446	Guanine

Name	Bg.				Bg.	Name
Adenine	0.206554	A	~	T	0.206554	Thymine
Cytosine	0.293446	C	~	G	0.293446	Guanine

Sequences

Primary Sequences	Number	Control Sequences	Number
Galaxy195-bedtools_GetFastaBed_Merged_and_Narrowed_Hyper_regions.fasta	107	primary sequences shuffled conserving 2-mer frequencies	1070

Primary Sequences	Number	Control Sequences	Number
Galaxy195-bedtools_GetFastaBed_Merged_and_Narrowed_Hyper_regions.fasta	107	primary sequences shuffled conserving 2-mer frequencies	1070

Motifs

Database	Source	Motif Count
HOCOMOCOv11 full HUMAN mono meme format	db/HUMAN/HOCOMOCOv11_full_HUMAN_mono_meme_format.meme	769
HOCOMOCOv11 full HUMAN mono meme format	db/HUMAN/HOCOMOCOv11_full_HUMAN_mono_meme_format.meme	769

AME version
5.1.15.1.1 (Release date: Wed Jan 29 15:00:42 2020 -0800Wed Jan 29 15:00:42 2020 -0800)
Copyright © Robert McLeay & Timothy Bailey meme-suite@uw.edu, 2009.

Command line summary

```
ame --verbose 1 --oc . --scoring avg --method fisher --hit-lo-fraction 0.25 --evalue-report-threshold 10.0 --control --shuffle-- --kmer 2
Galaxy195-bedtools_GetFastaBed_Merged_and_Narrowed_Hyper_regions.fasta db/HUMAN/HOCOMOCOv11_full_HUMAN_mono_meme_format.meme
```

AME
Analysis of Motif Enrichment

For further information on how to interpret these results please access http://meme-suite.org//doc/ame-output-format.html.
To get a copy of the MEME software please access http://meme-suite.org.

If you use AME in your research, please cite the following paper:
Robert McLeay and Timothy L. Bailey, "Motif Enrichment Analysis: A unified framework and method evaluation", *BMC Bioinformatics*, **11**:165, 2010, doi:10.1186/1471-2105-11-165. [full text]

ENRICHED MOTIFS | INPUT FILES | PROGRAM INFORMATION | RESULTS IN TSV FORMAT ? ? NEW | POSITIVE SEQUENCES FOR EACH MOTIF ? NEW | POSITIVE SEQUENCES FOR EACH MOTIF ? NEW

ENRICHED MOTIFS

Sequence motif score: avg_odds
Background model source: letter frequencies in (primary) sequences file (Galaxy196-bedtools_GetFastaBed_Merged_and_Narrowed_Hypo_regions.fasta)
Background model frequencies: 0.189588,0.310412,0.310412,0.189588
Total pseudocount added to a motif column: 0.1

Statistical test: Fisher's exact test (optimized over motif scores)
Labeling positives: all 42 primary sequences are labeled as 'positive'; all 1008 control sequences are labeled as 'negative'
Classifying positives: sequences with the motif scores ≥ 'TP Thresh' are classified as positive

E-value threshold for reporting results: 10
Sequence motif score: avg_odds
Background model source: letter frequencies in (primary) sequences file (Galaxy196-bedtools_GetFastaBed_Merged_and_Narrowed_Hypo_regions.fasta)
Background model frequencies: 0.189588,0.310412,0.310412,0.189588
Total pseudocount added to a motif column: 0.1

Statistical test: Fisher's exact test (optimized over motif scores)
Labeling positives: all 42 primary sequences are labeled as 'positive'; all 1008 control sequences are labeled as 'negative'
Classifying positives: sequences with the motif scores ≥ 'TP Thresh' are classified as positive

E-value threshold for reporting results: 10

Logo	Database ?	ID ?	Alt ID ?	*p*-value ?	*E*-value ?	TP Thresh ?	TP (%) ?

Logo	Database	ID	Alt ID	*p*-value	*E*-value	TP Thresh	TP (%)
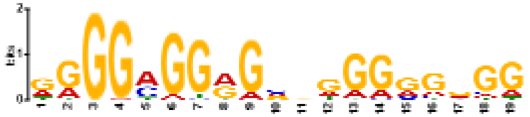	HOCOMOCOv11 full HUMAN mono meme format	KLF15_HUMAN.H11MO.0.A		2.03e-9	1.56e-6	1.69	36 (85.7%
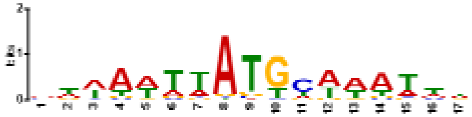	HOCOMOCOv11 full HUMAN mono meme format	PO3F3_HUMAN.H11MO.0.D		2.12e-9	1.63e-6	2.14	24 (57.1%
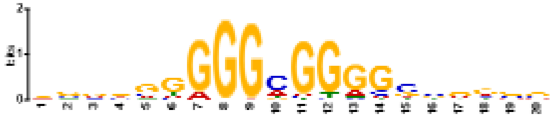	HOCOMOCOv11 full HUMAN mono meme format	SP3_HUMAN.H11MO.0.B		2.18e-9	1.68e-6	3.36	29 (69.0%
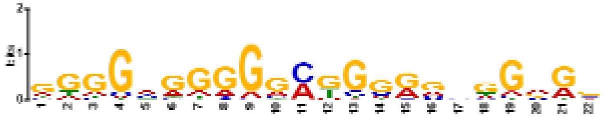	HOCOMOCOv11 full HUMAN mono meme format	PATZ1_HUMAN.H11MO.0.C		1.43e-8	1.10e-5	3.90	31 (73.8%
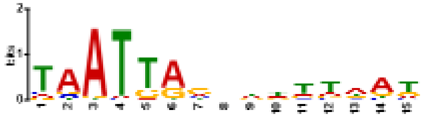	HOCOMOCOv11 full HUMAN mono meme format	GSX1_HUMAN.H11MO.0.D		1.52e-8	1.17e-5	5.47	15 (35.7%
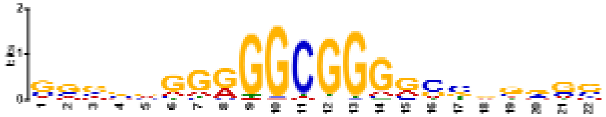	HOCOMOCOv11 full HUMAN mono meme format	SP2_HUMAN.H11MO.0.A		1.78e-8	1.37e-5	4.78	22 (52.4%
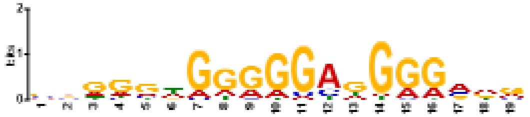	HOCOMOCOv11 full HUMAN mono meme format	ZBT17_HUMAN.H11MO.0.A		4.02e-8	3.09e-5	5.41	28 (66.7%
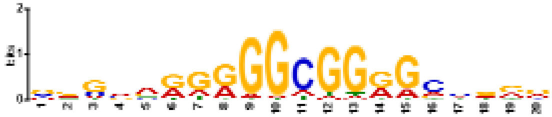	HOCOMOCOv11 full HUMAN mono meme format	SP4_HUMAN.H11MO.0.A		5.75e-8	4.42e-5	2.58	30 (71.4%
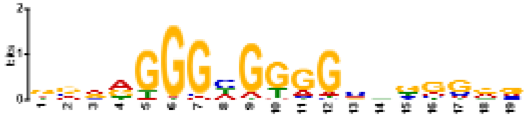	HOCOMOCOv11 full HUMAN mono meme format	KLF6_HUMAN.H11MO.0.A		5.98e-8	4.60e-5	3.20	28 (66.7%
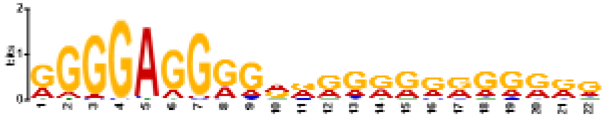	HOCOMOCOv11 full HUMAN mono meme format	ZN467_HUMAN.H11MO.0.C		6.78e-8	5.22e-5	3.87	29 (69.0%

Logo	Database	ID	Alt ID	*p*-value	*E*-value	TP Thresh	TP (%)
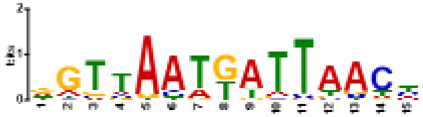	HOCOMOCOv11 full HUMAN mono meme format	HNF1A_HUMAN.H11MO.0.C		1.93e-7	1.48e-4	1.25	18 (42.9
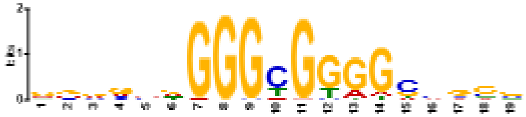	HOCOMOCOv11 full HUMAN mono meme format	KLF3_HUMAN.H11MO.0.B		2.46e-7	1.89e-4	2.97	24 (57.1
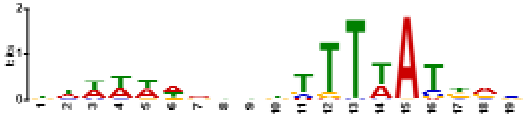	HOCOMOCOv11 full HUMAN mono meme format	HXC10_HUMAN.H11MO.0.D		2.47e-7	1.90e-4	1.11	24 (57.1
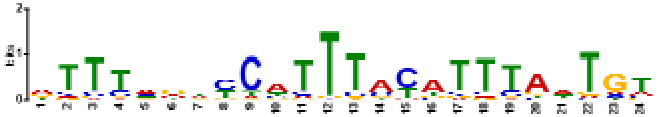	HOCOMOCOv11 full HUMAN mono meme format	Z354A_HUMAN.H11MO.0.C		3.45e-7	2.66e-4	4.21	16 (38.1
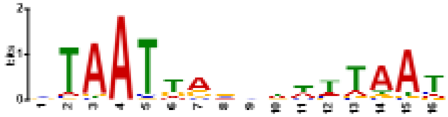	HOCOMOCOv11 full HUMAN mono meme format	GSX2_HUMAN.H11MO.0.D		4.21e-7	3.24e-4	5.49	13 (31.0
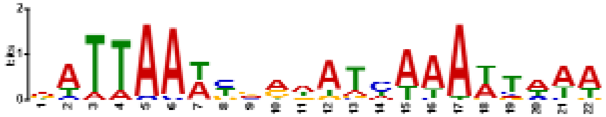	HOCOMOCOv11 full HUMAN mono meme format	ARI3A_HUMAN.H11MO.0.D		1.55e-6	1.19e-3	10.36	9 (21.4
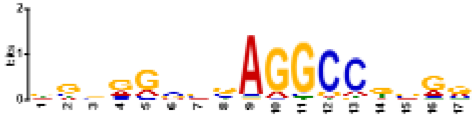	HOCOMOCOv11 full HUMAN mono meme format	ZFX_HUMAN.H11MO.1.A		2.03e-6	1.56e-3	2.17	29 (69.0
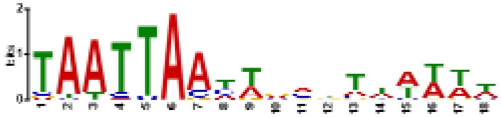	HOCOMOCOv11 full HUMAN mono meme format	LMX1B_HUMAN.H11MO.0.D		2.28e-6	1.75e-3	1.17	14 (33.3
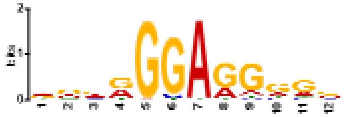	HOCOMOCOv11 full HUMAN mono meme format	VEZF1_HUMAN.H11MO.1.C		2.71e-6	2.09e-3	4.27	26 (61.9
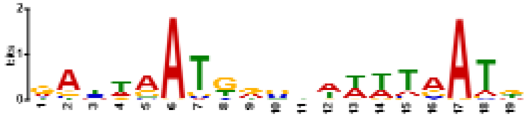	HOCOMOCOv11 full HUMAN mono meme format	NKX61_HUMAN.H11MO.0.B		2.79e-6	2.15e-3	5.66	13 (31.0

Logo	Database	ID	Alt ID	*p*-value	*E*-value	TP Thresh	TP (%)
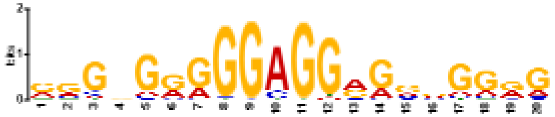	HOCOMOCOv11 full HUMAN mono meme format	WT1_HUMAN.H11MO.0.C		4.44e-6	3.42e-3	2.35	31 (73.8%
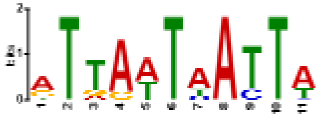	HOCOMOCOv11 full HUMAN mono meme format	ZFHX3_HUMAN.H11MO.0.D		4.56e-6	3.51e-3	2.46	9 (21.4%
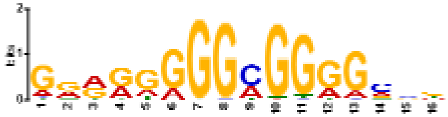	HOCOMOCOv11 full HUMAN mono meme format	SP4_HUMAN.H11MO.1.A		4.85e-6	3.73e-3	7.66	19 (45.2%
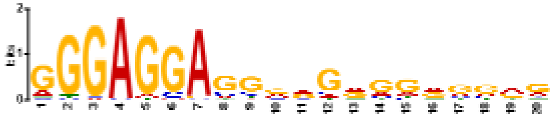	HOCOMOCOv11 full HUMAN mono meme format	ZN263_HUMAN.H11MO.0.A		5.50e-6	4.23e-3	1.70	32 (76.2%
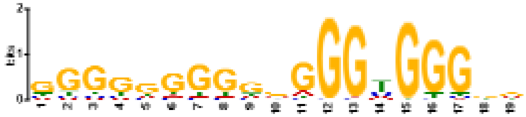	HOCOMOCOv11 full HUMAN mono meme format	TBX15_HUMAN.H11MO.0.D		5.83e-6	4.48e-3	1.72	31 (73.8%
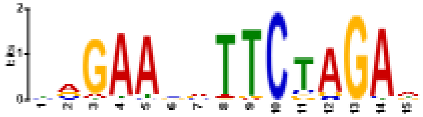	HOCOMOCOv11 full HUMAN mono meme format	HXB2_HUMAN.H11MO.0.D		8.75e-6	6.73e-3	2.86	11 (26.2%
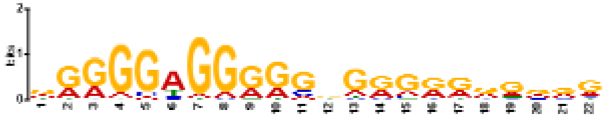	HOCOMOCOv11 full HUMAN mono meme format	MAZ_HUMAN.H11MO.0.A		1.27e-5	9.78e-3	2.96	33 (78.6%
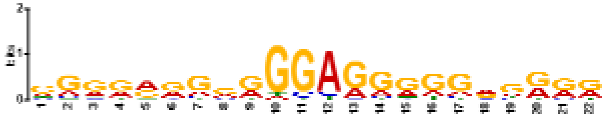	HOCOMOCOv11 full HUMAN mono meme format	VEZF1_HUMAN.H11MO.0.C		1.35e-5	1.04e-2	2.87	35 (83.3%
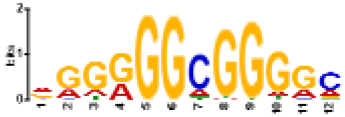	HOCOMOCOv11 full HUMAN mono meme format	SP2_HUMAN.H11MO.1.B		1.63e-5	1.25e-2	1.59	32 (76.2%
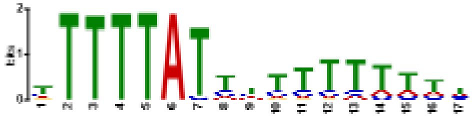	HOCOMOCOv11 full HUMAN mono meme format	CPEB1_HUMAN.H11MO.0.D		1.78e-5	1.37e-2	1.27	14 (33.3%

Logo	Database	ID	Alt ID	*p*-value	*E*-value	TP Thresh	TP (%)
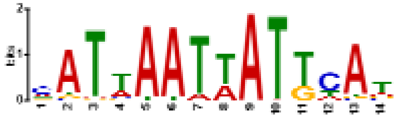	HOCOMOCOv11 full HUMAN mono meme format	PO4F1_HUMAN.H11MO.0.D		2.31e-5	1.78e-2	2.69	8 (19.0%
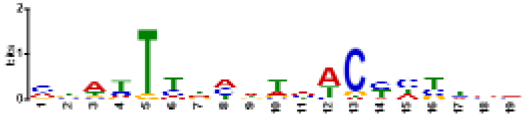	HOCOMOCOv11 full HUMAN mono meme format	MNX1_HUMAN.H11MO.0.D		2.81e-5	2.16e-2	5.30	18 (42.9%
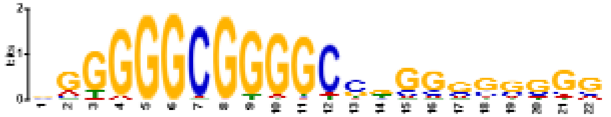	HOCOMOCOv11 full HUMAN mono meme format	SP1_HUMAN.H11MO.0.A		5.36e-5	4.12e-2	1.37	18 (42.9%
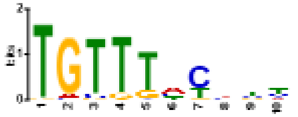	HOCOMOCOv11 full HUMAN mono meme format	FOXK1_HUMAN.H11MO.0.A		5.57e-5	4.28e-2	1.99	21 (50.0%
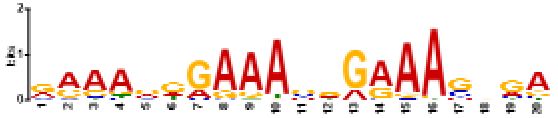	HOCOMOCOv11 full HUMAN mono meme format	IRF3_HUMAN.H11MO.0.B		5.72e-5	4.40e-2	2.86	24 (57.1%
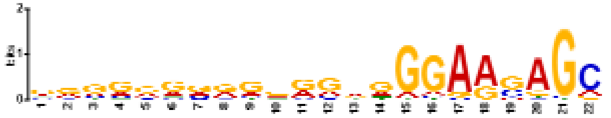	HOCOMOCOv11 full HUMAN mono meme format	ZN341_HUMAN.H11MO.0.C		6.82e-5	5.25e-2	7.97	22 (52.4%
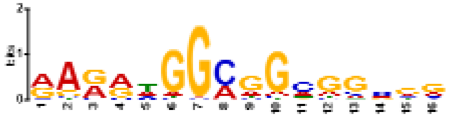	HOCOMOCOv11 full HUMAN mono meme format	TAF1_HUMAN.H11MO.0.A		6.99e-5	5.38e-2	1.78	28 (66.7%
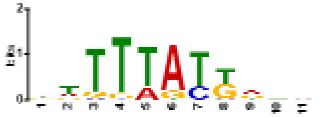	HOCOMOCOv11 full HUMAN mono meme format	HXD11_HUMAN.H11MO.0.D		7.86e-5	6.04e-2	3.36	15 (35.7%
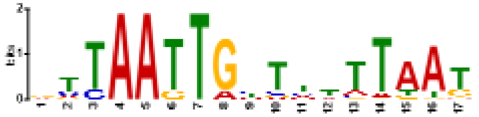	HOCOMOCOv11 full HUMAN mono meme format	HMX1_HUMAN.H11MO.0.D		9.03e-5	6.94e-2	3.52	7 (16.7%
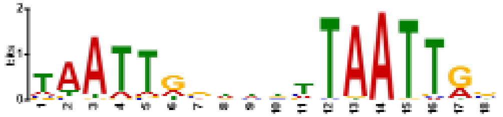	HOCOMOCOv11 full HUMAN mono meme format	MSX1_HUMAN.H11MO.0.D		9.96e-5	7.66e-2	2.54	8 (19.0%

Logo	Database	ID	Alt ID	p-value	E-value	TP Thresh	TP (%)
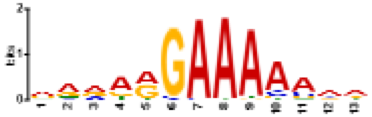	HOCOMOCOv11 full HUMAN mono meme format	PRDM6_HUMAN.H11MO.0.C		1.04e-4	8.02e-2	9.46	17 (40.5%
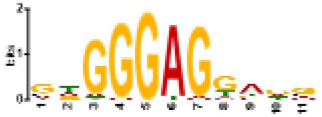	HOCOMOCOv11 full HUMAN mono meme format	WT1_HUMAN.H11MO.1.B		1.10e-4	8.42e-2	2.26	31 (73.8%
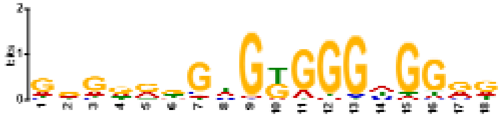	HOCOMOCOv11 full HUMAN mono meme format	EGR2_HUMAN.H11MO.0.A		1.18e-4	9.06e-2	1.12	35 (83.3%
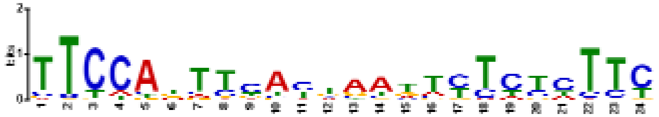	HOCOMOCOv11 full HUMAN mono meme format	ZFP82_HUMAN.H11MO.0.C		1.57e-4	1.20e-1	1.35	21 (50.0%
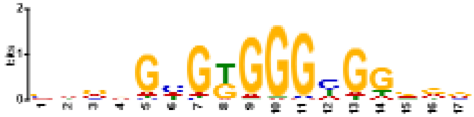	HOCOMOCOv11 full HUMAN mono meme format	EGR1_HUMAN.H11MO.0.A		1.69e-4	1.30e-1	2.05	24 (57.1%
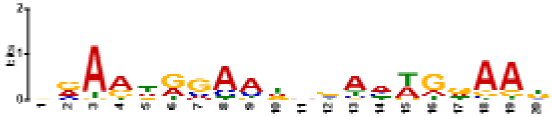	HOCOMOCOv11 full HUMAN mono meme format	ZN394_HUMAN.H11MO.0.C		1.70e-4	1.30e-1	1.57	30 (71.4%
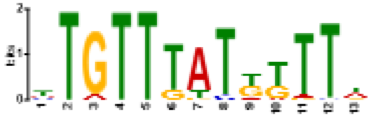	HOCOMOCOv11 full HUMAN mono meme format	FOXJ3_HUMAN.H11MO.0.A		1.88e-4	1.45e-1	1.34	10 (23.8%
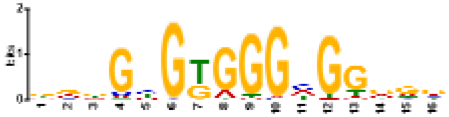	HOCOMOCOv11 full HUMAN mono meme format	EGR2_HUMAN.H11MO.1.A		2.07e-4	1.59e-1	2.44	22 (52.4%
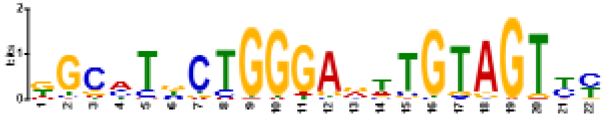	HOCOMOCOv11 full HUMAN mono meme format	THA11_HUMAN.H11MO.0.B		2.68e-4	2.06e-1	1.47	10 (23.8%
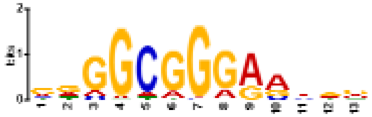	HOCOMOCOv11 full HUMAN mono meme format	E2F7_HUMAN.H11MO.0.B		2.69e-4	2.07e-1	3.00	19 (45.2%

Logo	Database	ID	Alt ID	*p*-value	*E*-value	TP Thresh	TP (%)
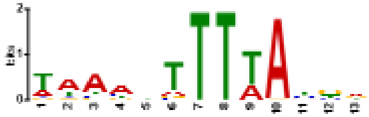	HOCOMOCOv11 full HUMAN mono meme format	HXA11_HUMAN.H11MO.0.D		2.82e-4	2.17e-1	4.05	13 (31.0%
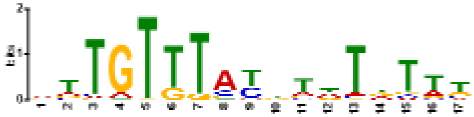	HOCOMOCOv11 full HUMAN mono meme format	FOXG1_HUMAN.H11MO.0.D		3.70e-4	2.84e-1	1.06	18 (42.9%
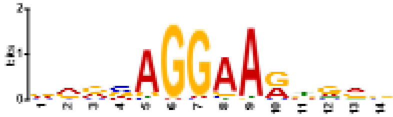	HOCOMOCOv11 full HUMAN mono meme format	ETV5_HUMAN.H11MO.0.C		3.90e-4	3.00e-1	4.21	22 (52.4%
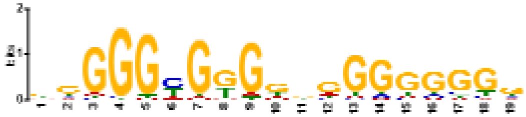	HOCOMOCOv11 full HUMAN mono meme format	KLF16_HUMAN.H11MO.0.D		4.30e-4	3.31e-1	2.38	23 (54.8%
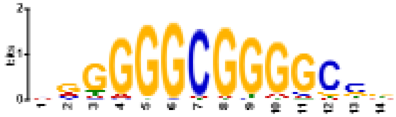	HOCOMOCOv11 full HUMAN mono meme format	SP1_HUMAN.H11MO.1.A		4.64e-4	3.57e-1	5.29	12 (28.6%
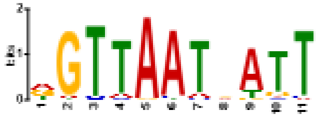	HOCOMOCOv11 full HUMAN mono meme format	HNF1B_HUMAN.H11MO.1.A		4.78e-4	3.68e-1	4.58	8 (19.0%
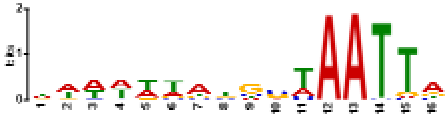	HOCOMOCOv11 full HUMAN mono meme format	DLX1_HUMAN.H11MO.0.D		5.92e-4	4.55e-1	5.38	10 (23.8%
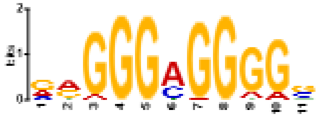	HOCOMOCOv11 full HUMAN mono meme format	MAZ_HUMAN.H11MO.1.A		6.94e-4	5.34e-1	5.07	20 (47.6%
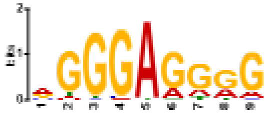	HOCOMOCOv11 full HUMAN mono meme format	PATZ1_HUMAN.H11MO.1.C		7.32e-4	5.63e-1	4.40	21 (50.0%
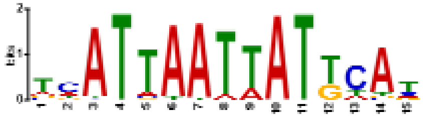	HOCOMOCOv11 full HUMAN mono meme format	PO4F3_HUMAN.H11MO.0.D		7.40e-4	5.69e-1	1.29	6 (14.3%

Logo	Database	ID	Alt ID	*p*-value	*E*-value	TP Thresh	TP (%)
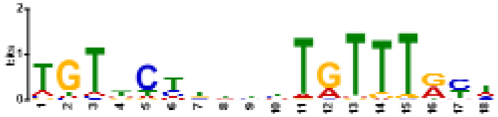	HOCOMOCOv11 full HUMAN mono meme format	ANDR_HUMAN.H11MO.0.A		7.92e-4	6.09e-1	11.78	10 (23.8%
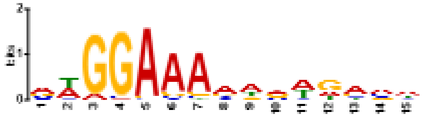	HOCOMOCOv11 full HUMAN mono meme format	NFAC1_HUMAN.H11MO.0.B		8.09e-4	6.22e-1	3.35	22 (52.4%
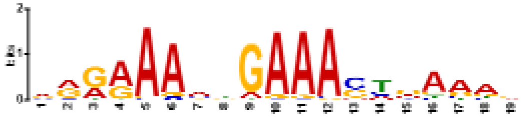	HOCOMOCOv11 full HUMAN mono meme format	STAT1_HUMAN.H11MO.1.A		8.13e-4	6.25e-1	5.31	16 (38.1%
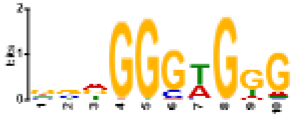	HOCOMOCOv11 full HUMAN mono meme format	SALL4_HUMAN.H11MO.0.B		8.44e-4	6.49e-1	3.74	20 (47.6%
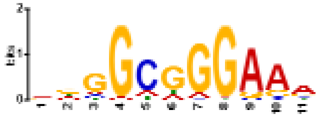	HOCOMOCOv11 full HUMAN mono meme format	E2F3_HUMAN.H11MO.0.A		9.05e-4	6.96e-1	1.79	26 (61.9%
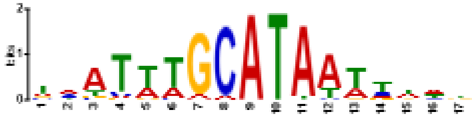	HOCOMOCOv11 full HUMAN mono meme format	PO3F4_HUMAN.H11MO.0.D		9.22e-4	7.09e-1	1.29	10 (23.8%
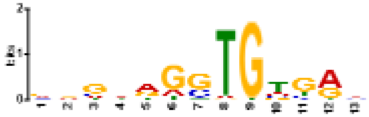	HOCOMOCOv11 full HUMAN mono meme format	TBX21_HUMAN.H11MO.0.A		9.25e-4	7.11e-1	1.11	36 (85.7%
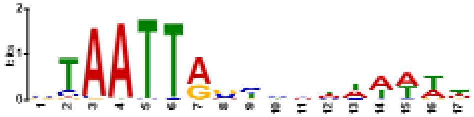	HOCOMOCOv11 full HUMAN mono meme format	GBX2_HUMAN.H11MO.0.D		9.37e-4	7.20e-1	3.19	11 (26.2%
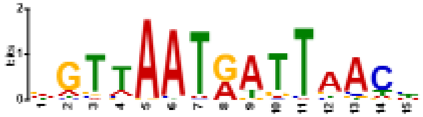	HOCOMOCOv11 full HUMAN mono meme format	HNF1B_HUMAN.H11MO.0.A		1.00e-3	7.71e-1	1.40	10 (23.8%
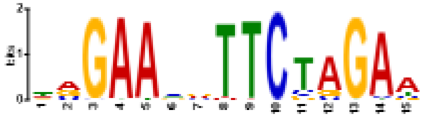	HOCOMOCOv11 full HUMAN mono meme format	HSF4_HUMAN.H11MO.0.D		1.13e-3	8.70e-1	40.70	4 (9.5%

Logo	Database	ID	Alt ID	*p*-value	*E*-value	TP Thresh	TP (%)
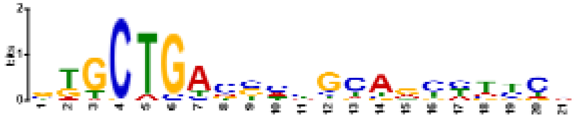	HOCOMOCOv11 full HUMAN mono meme format	MAFA_HUMAN.H11MO.0.D		1.16e-3	8.91e-1	1.21	23 (54.8%
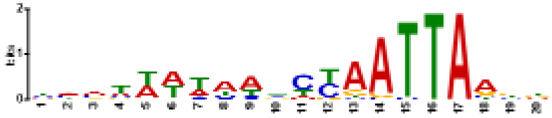	HOCOMOCOv11 full HUMAN mono meme format	DMBX1_HUMAN.H11MO.0.D		1.26e-3	9.70e-1	2.26	10 (23.8%
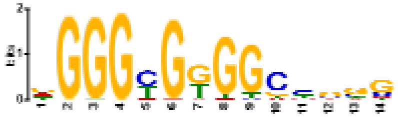	HOCOMOCOv11 full HUMAN mono meme format	KLF1_HUMAN.H11MO.0.A		1.39e-3	1.07e0	1.57	25 (59.5%
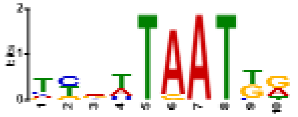	HOCOMOCOv11 full HUMAN mono meme format	BARX2_HUMAN.H11MO.0.D		1.43e-3	1.10e0	4.77	10 (23.8%
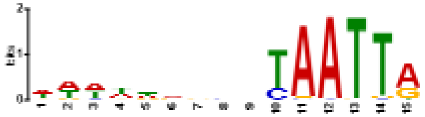	HOCOMOCOv11 full HUMAN mono meme format	DLX6_HUMAN.H11MO.0.D		1.50e-3	1.16e0	10.84	8 (19.0%
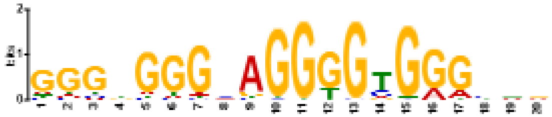	HOCOMOCOv11 full HUMAN mono meme format	TBX1_HUMAN.H11MO.0.D		1.70e-3	1.31e0	1.10	23 (54.8%
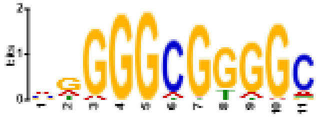	HOCOMOCOv11 full HUMAN mono meme format	KLF12_HUMAN.H11MO.0.C		1.72e-3	1.32e0	1.88	20 (47.6%
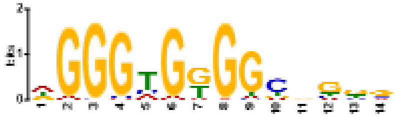	HOCOMOCOv11 full HUMAN mono meme format	KLF5_HUMAN.H11MO.0.A		1.73e-3	1.33e0	2.59	22 (52.4%
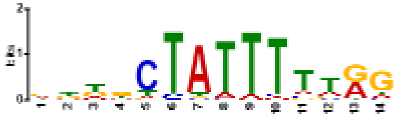	HOCOMOCOv11 full HUMAN mono meme format	MEF2B_HUMAN.H11MO.0.A		2.13e-3	1.64e0	43.81	5 (11.9%
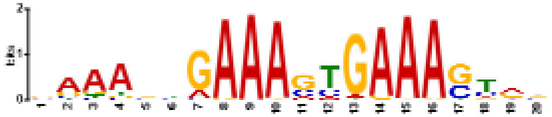	HOCOMOCOv11 full HUMAN mono meme format	IRF2_HUMAN.H11MO.0.A		2.42e-3	1.86e0	2.48	11 (26.2%

Logo	Database	ID	Alt ID	*p*-value	*E*-value	TP Thresh	TP (%)
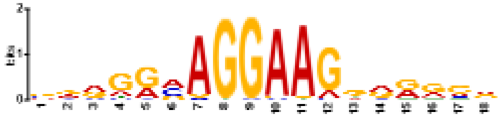	HOCOMOCOv11 full HUMAN mono meme format	FLI1_HUMAN.H11MO.0.A		2.42e-3	1.86e0	1.89	27 (64.3%
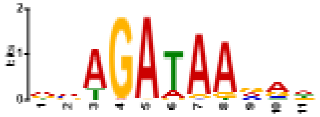	HOCOMOCOv11 full HUMAN mono meme format	GATA3_HUMAN.H11MO.0.A		2.52e-3	1.94e0	2.39	20 (47.6%
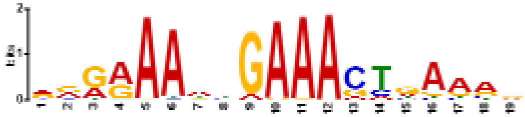	HOCOMOCOv11 full HUMAN mono meme format	STAT2_HUMAN.H11MO.0.A		2.55e-3	1.96e0	1.12	16 (38.1%
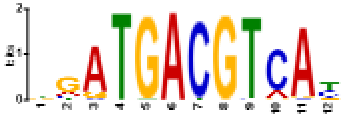	HOCOMOCOv11 full HUMAN mono meme format	JDP2_HUMAN.H11MO.0.D		2.66e-3	2.04e0	1.61	4 (9.5%
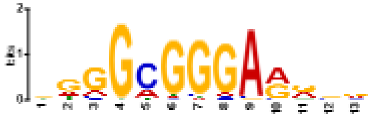	HOCOMOCOv11 full HUMAN mono meme format	E2F6_HUMAN.H11MO.0.A		3.01e-3	2.31e0	4.09	17 (40.5%
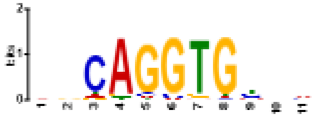	HOCOMOCOv11 full HUMAN mono meme format	ID4_HUMAN.H11MO.0.D		3.31e-3	2.54e0	1.76	21 (50.0%
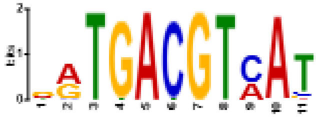	HOCOMOCOv11 full HUMAN mono meme format	CREB5_HUMAN.H11MO.0.D		3.38e-3	2.60e0	2.03	3 (7.1%
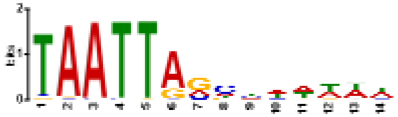	HOCOMOCOv11 full HUMAN mono meme format	VSX1_HUMAN.H11MO.0.D		3.55e-3	2.73e0	1.16	13 (31.0%
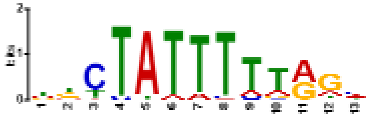	HOCOMOCOv11 full HUMAN mono meme format	MEF2A_HUMAN.H11MO.0.A		3.86e-3	2.97e0	1.36	16 (38.1%
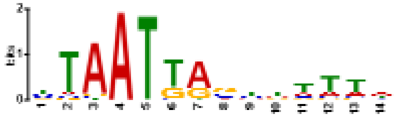	HOCOMOCOv11 full HUMAN mono meme format	HXB3_HUMAN.H11MO.0.D		3.87e-3	2.98e0	11.21	7 (16.7%

Logo	Database	ID	Alt ID	p-value	E-value	TP Thresh	TP (%)
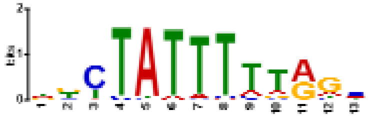	HOCOMOCOv11 full HUMAN mono meme format	MEF2C_HUMAN.H11MO.0.A		4.18e-3	3.21e0	1.34	16 (38.1%
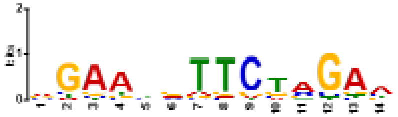	HOCOMOCOv11 full HUMAN mono meme format	HSF2_HUMAN.H11MO.0.A		4.24e-3	3.26e0	4.29	10 (23.8%
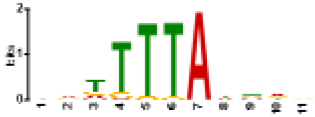	HOCOMOCOv11 full HUMAN mono meme format	HXD12_HUMAN.H11MO.0.D		4.29e-3	3.30e0	1.39	23 (54.8%
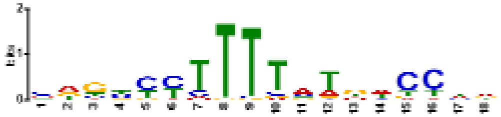	HOCOMOCOv11 full HUMAN mono meme format	ZN350_HUMAN.H11MO.0.C		4.31e-3	3.32e0	26.21	8 (19.0%
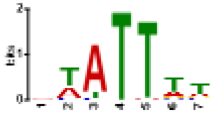	HOCOMOCOv11 full HUMAN mono meme format	HMGA1_HUMAN.H11MO.0.D		4.35e-3	3.34e0	1.36	26 (61.9%
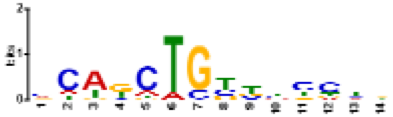	HOCOMOCOv11 full HUMAN mono meme format	LYL1_HUMAN.H11MO.0.A		4.40e-3	3.39e0	1.23	38 (90.5%
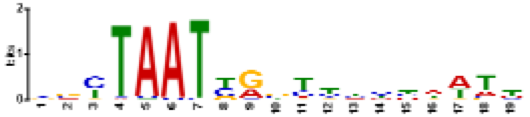	HOCOMOCOv11 full HUMAN mono meme format	VENTX_HUMAN.H11MO.0.D		4.81e-3	3.70e0	1.37	14 (33.3%
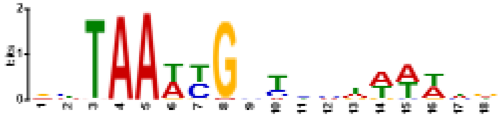	HOCOMOCOv11 full HUMAN mono meme format	BARH2_HUMAN.H11MO.0.D		4.91e-3	3.78e0	5.33	7 (16.7%
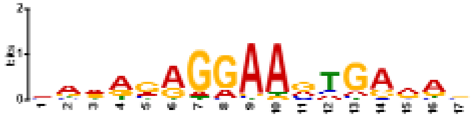	HOCOMOCOv11 full HUMAN mono meme format	BC11A_HUMAN.H11MO.0.A		5.17e-3	3.98e0	5.40	18 (42.9%
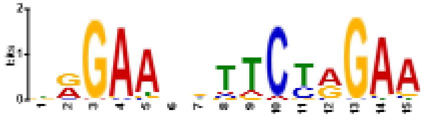	HOCOMOCOv11 full HUMAN mono meme format	HSF1_HUMAN.H11MO.0.A		5.28e-3	4.06e0	31.05	6 (14.3%

Logo	Database	ID	Alt ID	*p*-value	*E*-value	TP Thresh	TP (%)
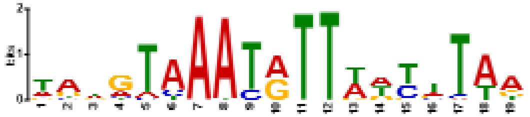	HOCOMOCOv11 full HUMAN mono meme format	FOXD2_HUMAN.H11MO.0.D		5.46e-3	4.20e0	1.51	4 (9.5%)
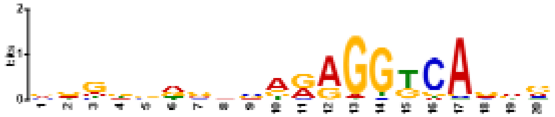	HOCOMOCOv11 full HUMAN mono meme format	RXRA_HUMAN.H11MO.0.A		6.18e-3	4.75e0	1.36	35 (83.3%
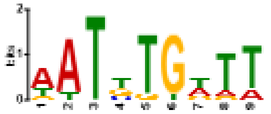	HOCOMOCOv11 full HUMAN mono meme format	FOXP3_HUMAN.H11MO.0.D		6.19e-3	4.76e0	2.67	15 (35.7%
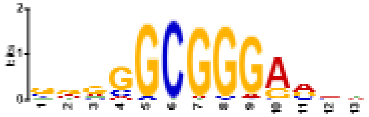	HOCOMOCOv11 full HUMAN mono meme format	E2F4_HUMAN.H11MO.0.A		6.98e-3	5.37e0	1.75	18 (42.9%
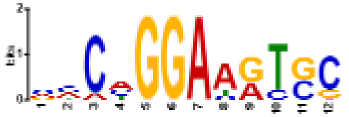	HOCOMOCOv11 full HUMAN mono meme format	ELK3_HUMAN.H11MO.0.D		7.41e-3	5.70e0	1.11	18 (42.9%
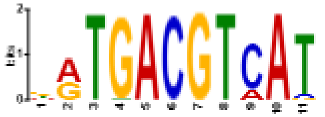	HOCOMOCOv11 full HUMAN mono meme format	ATF7_HUMAN.H11MO.0.D		7.65e-3	5.88e0	1.54	3 (7.1%)
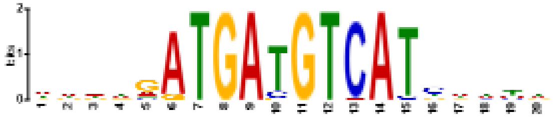	HOCOMOCOv11 full HUMAN mono meme format	ATF2_HUMAN.H11MO.1.B		7.65e-3	5.88e0	1.77	3 (7.1%)
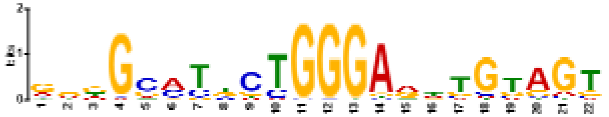	HOCOMOCOv11 full HUMAN mono meme format	ZNF76_HUMAN.H11MO.0.C		7.71e-3	5.93e0	5.74	8 (19.0%
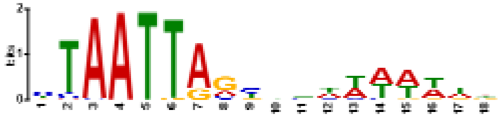	HOCOMOCOv11 full HUMAN mono meme format	LHX9_HUMAN.H11MO.0.D		8.11e-3	6.24e0	1.04	10 (23.8%
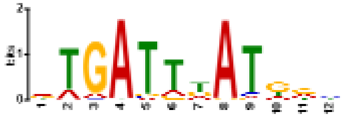	HOCOMOCOv11 full HUMAN mono meme format	MEIS1_HUMAN.H11MO.0.A		8.19e-3	6.30e0	1.64	14 (33.3%

Logo	Database	ID	Alt ID	*p*-value	*E*-value	TP Thresh	TP (%)
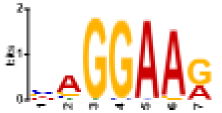	HOCOMOCOv11 full HUMAN mono meme format	ETV7_HUMAN.H11MO.0.D		8.35e-3	6.42e0	2.95	21 (50.0%
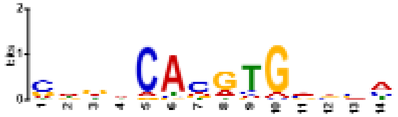	HOCOMOCOv11 full HUMAN mono meme format	CLOCK_HUMAN.H11MO.0.C		8.56e-3	6.58e0	3.91	6 (14.3%
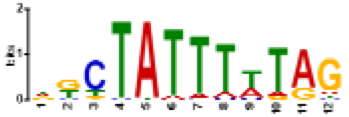	HOCOMOCOv11 full HUMAN mono meme format	MEF2D_HUMAN.H11MO.0.A		8.96e-3	6.89e0	2.15	11 (26.2%
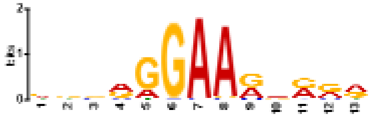	HOCOMOCOv11 full HUMAN mono meme format	ETS2_HUMAN.H11MO.0.B		9.88e-3	7.60e0	7.82	14 (33.3%
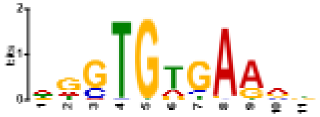	HOCOMOCOv11 full HUMAN mono meme format	BRAC_HUMAN.H11MO.1.B		9.94e-3	7.64e0	1.11	28 (66.7%
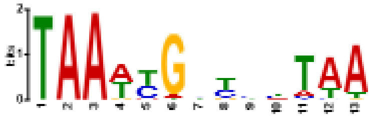	HOCOMOCOv11 full HUMAN mono meme format	BARH1_HUMAN.H11MO.0.D		1.04e-2	8.03e0	14.03	5 (11.9%
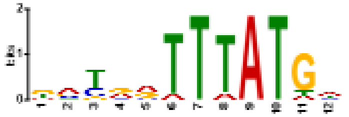	HOCOMOCOv11 full HUMAN mono meme format	HXA10_HUMAN.H11MO.0.C		1.08e-2	8.32e0	1.16	14 (33.3%
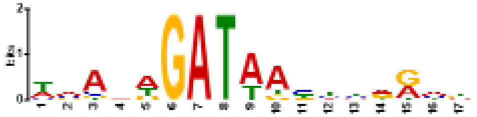	HOCOMOCOv11 full HUMAN mono meme format	GATA5_HUMAN.H11MO.0.D		1.15e-2	8.81e0	1.36	15 (35.7%
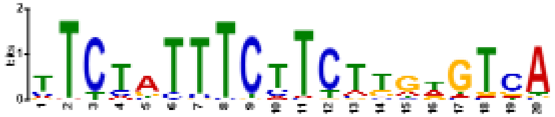	HOCOMOCOv11 full HUMAN mono meme format	ZFP28_HUMAN.H11MO.0.C		1.16e-2	8.93e0	5.44	8 (19.0%
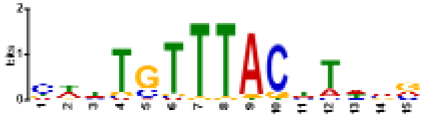	HOCOMOCOv11 full HUMAN mono meme format	FOXD1_HUMAN.H11MO.0.D		1.20e-2	9.23e0	1.44	13 (31.0%

Input Files

Alphabet

Background source: built from the (primary) sequences

Background source: built from the (primary) sequences

Name	Bg.				Bg.	Name
Adenine	0.189588	A	~	T	0.189588	Thymine
Cytosine	0.310412	C	~	G	0.310412	Guanine

Name	Bg.				Bg.	Name
Adenine	0.189588	A	~	T	0.189588	Thymine
Cytosine	0.310412	C	~	G	0.310412	Guanine

Sequences

Primary Sequences	Number	Control Sequences	Number
Galaxy196-bedtools_GetFastaBed_Merged_and_Narrowed_Hypo_regions.fasta	42	primary sequences shuffled conserving 2-mer frequencies	1008

Primary Sequences	Number	Control Sequences	Number
Galaxy196-bedtools_GetFastaBed_Merged_and_Narrowed_Hypo_regions.fasta	42	primary sequences shuffled conserving 2-mer frequencies	1008

Motifs

Database	Source	Motif Count
HOCOMOCOv11 full HUMAN mono meme format	db/HUMAN/HOCOMOCOv11_full_HUMAN_mono_meme_format.meme	769
HOCOMOCOv11 full HUMAN mono meme format	db/HUMAN/HOCOMOCOv11_full_HUMAN_mono_meme_format.meme	769

AME version
5.1.15.1.1 (Release date: Wed Jan 29 15:00:42 2020 -0800Wed Jan 29 15:00:42 2020 -0800)
Copyright © Robert McLeay & Timothy Bailey meme-suite@uw.edu, 2009.

Command line summary

```
ame --verbose 1 --oc . --scoring avg --method fisher --hit-lo-fraction 0.25 --evalue-report-threshold 10.0 --control --shuffle-- --kmer 2
Galaxy196-bedtools_GetFastaBed_Merged_and_Narrowed_Hypo_regions.fasta db/HUMAN/HOCOMOCOv11_full_HUMAN_mono_meme_format.meme
```
