# Supplementary material for: Multi-omics integration identifies key upstream regulators of pathomechanisms in hypertrophic cardiomyopathy due to truncating MYBPC3 mutations
Source: Clin Epigenetics. 2021 Mar 23;13:61. doi: 10.1186/s13148-021-01043-3 (PMC7989210; doi:10.1186/s13148-021-01043-3)
Supplement: Supplementary file 5 — Additional file 5: Figure S5 The acetylation and mRNA levels of the wildtype and mutant MYBPC3 alleles in all samples [file 13148_2021_1043_MOESM5_ESM.pdf]

Supplementary Figure 5

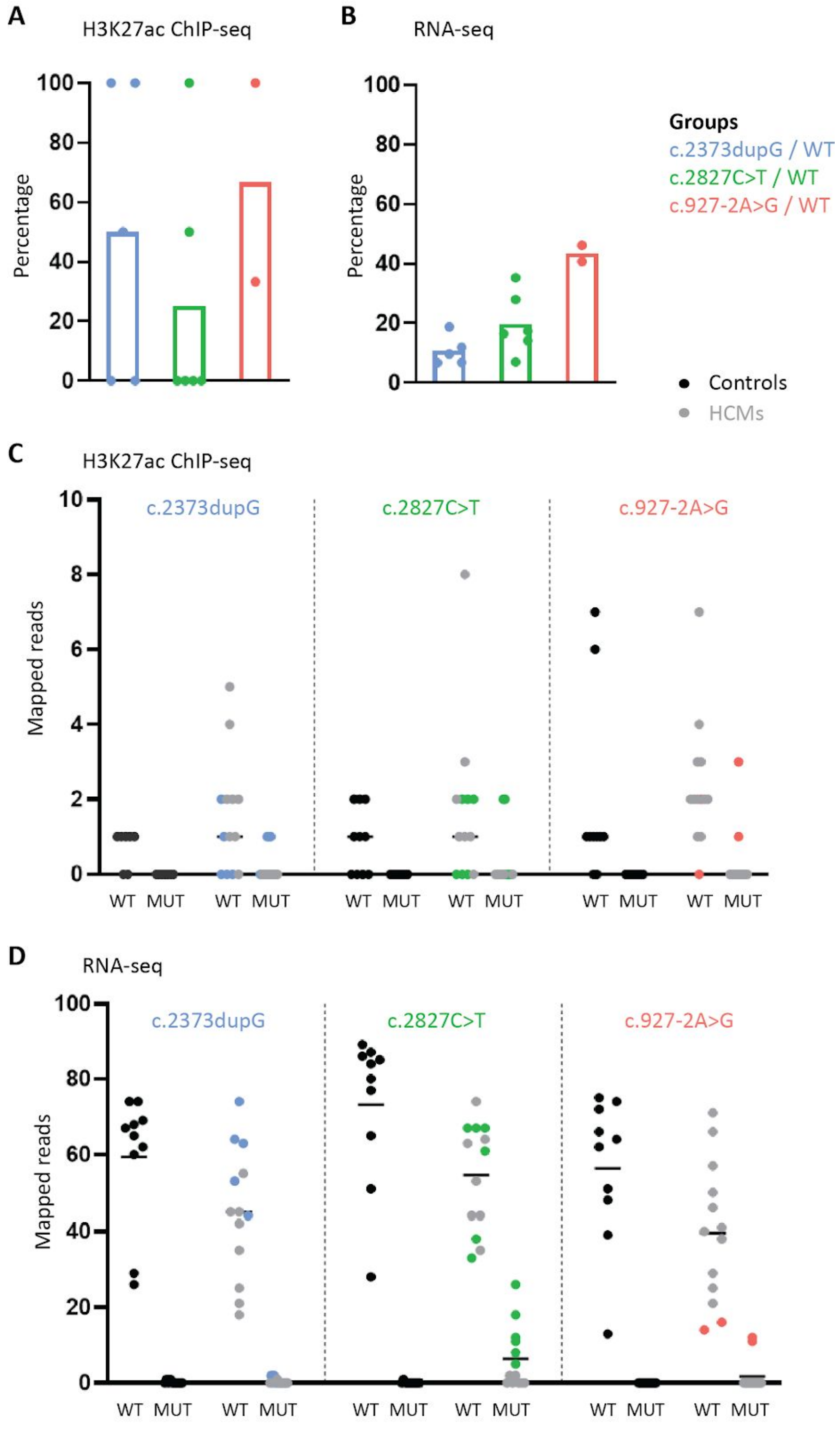

Allele-specific expression of the three truncating *MYBPC3* mutations in HCM samples. **(A)** The ratio between mapped H3K27ac ChIP-seq reads in the mutation allele and in the wildtype (WT) allele. Dots represent the ratio for each heterozygous patient. Dots represent the ratio for each heterozygous patient. **(B)** The ratio between mapped RNA-seq reads in the mutation allele and in the WT allele. **(C)** H3K27ac

ChIP-seq reads that were mapped to c.2373dupG, c.2827C>T, and c.927-2A>G mutation sites in all samples. For each mutation site, control samples (derived from donors with wildtype/wildtype alleles) are shown in black, and HCM samples derived from patients with heterozygous mutation (mutated/wildtype alleles) are highlighted in a mutation-specific color (blue=samples from 5 HCM patients with c.2373dupG, green=samples from 6 HCM patients with c.2827C>T, orange=samples from 2 HCM patients with c.927-2A>G), whereas the remaining HCM samples (with no mutations in the pre-defined sites) are shown in grey. **(D)** RNA-seq reads that were mapped to c.2373dupG, c.2827C>T, and c.927-2A>G mutation sites in all samples. In each mutation site, control samples (derived from donors with wildtype/wildtype alleles) are shown in black, and HCM samples with the examined heterozygous mutation are highlighted in a mutation-specific color (blue=c.2373dupG, green=c.2827C>T, orange=c.927-2A>G), whereas the remaining HCM samples are shown in grey.
